# Global burden of schizophrenia in 204 countries and regions from 1990 to 2021 and machine learning-based projections to 2036: an analysis of the 2021 global burden of disease study

**DOI:** 10.3389/fpsyt.2025.1684168

**Published:** 2026-01-27

**Authors:** Zhenyu Feng, Xuesong Shan, Baowen Fan, Ling Yang, Fang Gong

**Affiliations:** 1 Department of General Practice, The Affiliated Yongchuan Hospital of Chongqing Medical University, Chongqing Medical University, Chongqing, China; 2 Department of Neurosurgery, The Second Affiliated Hospital, Jiangxi Medical College, Nanchang University, Nanchang, Jiangxi, China; 3 Department of Neurology, The Affiliated Yongchuan Hospital of Chongqing Medical University, Chongqing Medical University, Chongqing, China; 4 The Affiliated Yongchuan Hospital of Chongqing Medical University, Chongqing Medical University, Chongqing, China

**Keywords:** global burden of disease, schizophrenia, incidence, disability-adjusted life years, prediction

## Abstract

**Background:**

Schizophrenia is a chronic mental disorder and one of the greatest contributors to the global burden of disease. This study aimed to analyze the global burden of schizophrenia from 1990 to 2021 and predict its future trends.

**Methods:**

Data from the 2021 Global Burden of Disease (GBD) study were used. Trend analysis was conducted using the estimated annual percentage change (EAPC), Joinpoint regression, and age-period-cohort analysis. Future projections were generated using the Bayesian age-period-cohort (BAPC) model and time-series machine learning (ML) models.

**Results:**

2021, schizophrenia was estimated to affect approximately 23.6 million individuals worldwide, with 1.22 million new cases reported globally. This disorder accounted for 14.82 million disability-adjusted life years (DALYs) lost. From 1990 to 2021, the age-standardized incidence rate (ASIR) exhibited a declining trend with an EAPC of −0.04% (95% UI: −0.04% to −0.03%), whereas the age-standardized prevalence rate (ASPR) and age-standardized disability-adjusted life years rate (ASDR) demonstrated upward trajectories, showing EAPCs of 0.03% (95% UI: 0.02% to 0.04%) and 0.04%(95% UI: −0.03% to −0.05%), respectively. The peak age of onset was 20–24 years, while prevalence and DALYs peaked at 30–34 years, with males exhibiting a higher disease burden. The ARIMA, PROPHET, and Elastic Net models demonstrated superior predictive performance for forecasting future trends of global ASIR, ASPR, and ASDR. Projections suggest a continued global decline in ASIR, while ASPR and ASDR are expected to rise in the future.

**Conclusion:**

Our research indicates that the ASIR, ASPR, and ASDR of schizophrenia exhibit significant correlations with gender, socio-demographic index (SDI) and regions. From 2022 to 2036, while the global ASIR of schizophrenia may decline, both the ASPR and ASDR are projected to rise. The escalating disease burden of schizophrenia poses a significant challenge for countries across all development levels.

## Introduction

Schizophrenia is a chronic psychiatric disorder characterized by three core clinical features: positive symptoms (e.g., hallucinations, delusions), negative symptoms (e.g., diminished motivation), and cognitive deficits (1). The disorder imposes profound functional impairments, as persistent cognitive dysfunction renders approximately 90% of patients unable to maintain employment or independent living (2). Furthermore, schizophrenia is associated with a life expectancy reduction of approximately 20 years relative to the general population, alongside elevated risks of comorbidities such as cardiovascular diseases, endocrine disorders, and infections (3, 4). These challenges collectively exacerbate the socioeconomic burden on individuals, healthcare systems, and societies. Globally, schizophrenia contributes to 14.8 million DALYs (5).

The prevalence of schizophrenia continues to rise persistently, with males exhibiting a 1.42-fold higher relative risk compared to females (6, 7). The disease burden demonstrates a distinct age-related pattern, peaking around 30 years in both sexes (8). The disparities in disease burden between countries and regions may be linked to their levels of economic development. The disease burden in high-income regions is approximately twice that of low-income regions (5). For instance, the annual societal cost per capita reaches 94, 587 in Norway but drops to 819 in Nigeria. Productivity losses attributable to schizophrenia account for 32%–83% of total societal costs across nations, while direct healthcare expenditures range from 11% to 87% (9) These stark contrasts underscore significant inequities in the quality and availability of care across regions.

Previous GBD studies have documented trends in schizophrenia from 1990 to 2019 (7). Recent GBD research has also used GBD 2021 data to analyze the developmental trajectory of the global and regional (e.g., China) burden of schizophrenia, providing projections up to 2050 through traditional statistical models such as the BAPC framework (10–12). However, relying on a single prediction method may have limitations in terms of forecasting accuracy. Therefore, we applied statistical models and machine learning models to predict the disease trajectory from 2022 to 2036. This approach can capture certain nuances that may be overlooked by traditional forecasting methods, thereby improving prediction accuracy (13). By integrating these temporal analyses and projections, this work aims to elucidate key challenges in schizophrenia management and provide targeted strategies to mitigate its global burden.

## Methods

### Materials

The schizophrenia data in this study were derived from the GBD 2021 study(https://ghdx.healthdata.org/gbd-2021/sources). As a comprehensive epidemiological investigation, the GBD 2021 study systematically estimates disease burden by analyzing 371 diseases/injuries and 88 risk factors across 204 countries and territories from 1990 to 2021 (5). This dataset was compiled by the Institute for Health Metrics and Evaluation (IHME) at the University of Washington. The institutional review board of the University of Washington granted an exemption for informed consent (https://www.healthdata.org/research-analysis/gbd) (14).

The GBD study categorizes locations into 21 regions based on sociodemographic similarity and geographic proximity to compare disease burdens across nations and territories. Development status is quantified using the socio-demographic index (SDI), a composite metric scaled from 0 (theoretical minimum development) to 100 (theoretical maximum development) (15). Countries are stratified into five SDI quintiles: low, low-middle, middle, high-middle, and high.

### Statistical analysis

Key metrics, including counts and age-standardized rate (ASR) of prevalence, incidence, and disability-adjusted life years (DALYs), were calculated at the global, regional, national, and sex-specific levels. ASR was used to eliminate the influence of population age structure differences. The global distribution of age-standardized incidence rate (ASIR), age-standardized prevalence rate (ASPR), and age-standardized disability-adjusted life years rate (ASDR) for schizophrenia across 204 countries was visualized using choropleth maps. To quantify temporal trends, the estimated annual percentage change (EAPC) was computed for ASIR, ASPR, and ASDR through linear regression model. The EAPC is a widely used measure to summarize trends in age-standardized rates over a specified time interval, reflecting their temporal dynamics (16). Joinpoint regression analysis was employed to identify significant inflection points in trends, calculating the Annual Percentage Change (APC) and Average Annual Percentage Change (AAPC) to distinguish true temporal shifts from random fluctuations (17). Our study employed age-period-cohort analysis to disentangle the effects of age, temporal period, and birth cohort on temporal trends in disease burden (18). Decomposition analysis further explored drivers of schizophrenia burden changes, such as population growth, aging, and epidemiology (19). Frontier analysis compared current burden levels with projected benchmarks to evaluate gaps between observed and expected outcomes (20). Health inequality analysis of incidence rate, prevalence rate, and DALYs was conducted to assess the relationship between health status, healthcare resources, and the SDI across different countries or regions.

In forecasting the future trends of ASPR, ASIR, and DALY for schizophrenia, two distinct predictive approaches were employed: Bayesian age-period-cohort (BAPC) modeling and time series analysis integrating statistical models and machine learning models. The BAPC model, widely utilized in GBD database analyses, operates within a Bayesian framework built upon traditional Generalized Linear Models. This model dynamically integrates age, period, and cohort effects while demonstrating exceptional capability in handling complex, high-dimensional, and sparse datasets (21). Its flexibility and robustness in processing time-series data make it particularly suitable for long-term disease burden projections. For time series predictions, six algorithms were implemented to forecast schizophrenia-related metrics from 2022 to 2036: Autoregressive Integrated Moving Average (ARIMA), Prophet, Multivariate Adaptive Regression Splines (MARS), Elastic Net, Random Forest, and eXtreme Gradient Boosting (XGBoost). ARIMA is a traditional statistical model, while the other five models belong to machine learning models. However, some of these models also incorporate elements of traditional statistical methods (e.g., Prophet).The ARIMA model combines autoregressive and moving average components with differencing to enhance time series prediction accuracy (22). Developed by Facebook, Prophet specializes in automated time series forecasting through trend decomposition (23). MARS, a nonparametric regression method introduced by Friedman, effectively captures nonlinear relationships in large-scale time series data (24). Elastic Net generates sparse models with strong predictive performance, particularly advantageous for multivariate linear combinations and big data scenarios (25). Random Forest enhances accuracy and generalization through ensemble decision trees (26), while XGBoost optimizes predictive efficiency through gradient boosting with enhanced flexibility and portability (27). Then, we performed rolling cross-validation on these six models and selected the optimal model with high R² and low errors using six evaluation metrics: Mean Squared Error (MSE), Root Mean Squared Error (RMSE), Mean Absolute Error (MAE), Mean Absolute Percentage Error (MAPE), Mean Absolute Scaled Error (MASE), and R-squared (R²). The selected optimal model and its evaluation metrics are presented in **Supplementary Table 1**.

All statistical analyses, except Joinpoint regression (version 5.3.0), were conducted using R software (version 4.4.1). A two-sided p-value <0.05 was considered statistically significant. BAPC modeling was performed using the R package “BAPC”. For predictive modeling, we employed a diverse set of techniques. These methods included random forests (via the R package “randomForest”), Prophet (via the R package “forecast”), MARS (via the R package “earth”), Elastic Net (via the R package “glmnet”) and XGBoost (via the R package “xgboost”).

## Results

### Schizophrenia varies across different genders, SDI and countries

In 2021, there were approximately 1, 223, 221 (95% UI:1, 008, 219 to 1, 473, 083) incident cases of schizophrenia globally, representing a 38.5% increase compared to 1990 (883, 493 cases; 95% UI: 721, 489 to 1, 065, 740). However, the ASIR showed a slight decline, decreasing from 15.64 (95% UI: 13.04 to 18.62) per 100, 000 population in 1990 to 15.43 (95% UI: 12.74 to 18.62) in 2021. This trend corresponded to an EAPC of −0.04% (95% UI: −0.04% to −0.03%), indicating a marginal reduction in global incidence over the past three decades. The most pronounced decline occurred in the middle SDI quintile (EAPC: −0.11%; 95% UI: −0.12% to −0.10%), followed by smaller reductions in low SDI quintiles (EAPC: −0.01%; 95% UI: −0.02% to 0.01%) and low-middle SDI quintiles (EAPC: −0.02%; 95% UI: −0.02% to −0.01%). In contrast, these regions demonstrated rising ASIR trends. The high-middle SDI quintile showed the steepest increase (EAPC: 14.57%; 95% UI: 12.13% to 17.41%), while the high SDI quintile experienced a minimal upward trend (EAPC: 0.04%; 95% UI: 0 to 0.08%) (**Table 1**). The global number of prevalent schizophrenia cases increased from 13, 621, 402 (95% UI: 11, 333, 557 to 16, 166, 722) in 1990 to 23182109 (95% UI:19203759 to 27423876). Concurrently, the ASPR rose from 261.98 (95% UI: 218.13 to 308.72) to 275.78 (95% UI: 229.94 to 324.02) per 100, 000 population, with an EAPC of 0.03% (95% UI: 0.02% to 0.04%). While the high SDI quintile showed no significant trend (EAPC: 0%; 95% UI: −0.04% to 0.03%), all other SDI groups exhibited increasing ASPR, most notably the high-middle SDI quintile (EAPC: 0.22%; 95% UI: 0.20% to 0.24%) (**Table 1**). DALYs attributable to schizophrenia surged from 8, 762, 312 (95% UI: 6, 477, 261 to 11, 263, 750) in 1990 to 14, 816, 611 (95% UI: 10, 926, 460 to 19, 095, 362) in 2021. The ASDR showed minimal change, increasing marginally from 176.61 (95% UI: 130.85 to 226.02) to 177.75 (95% UI: 131.51 to 228.80) per 100, 000 population (EAPC: 0.04%; 95% UI: 0.03% to 0.05%). Notably, the high SDI quintile was the only group with declining ASDR (EAPC: −0.01%, 95% UI: −0.05% to 0.03%), whereas the high-middle SDI quintile demonstrated the largest increase (ASDR: 171.35 to 185.37 per 100, 000; EAPC: 0.24%; 95% UI: 0.22% to 0.26%) (**Table 1**).

**Table 1:** Counts and age-standardized incidence, prevalence, and DALYs rates of schizophrenia in 2021, and their EAPC in ASR form 1990 to 2021, by sex, SDI quintile, and GBD region.

Group	Incidence (95% UI)			Prevalence (95% UI)			DALYs (95% UI)		
	2021 counts	2021 ASR per 100, 000 people	EAPC in ASR, 1990–2021	2021 counts	2021 ASR per 100, 000 people	EAPC in ASR, 1990–2021	2021 counts	2021 ASR per 100, 000 people	EAPC in ASR, 1990–2021
**Global**	1223221 (1008219 to 1473083)	15.43 (12.74 to 18.62)	−0.04 (−0.04 to −0.03)	23182109 (19203759 to 27423876)	277.71 (229.77 to 329.06)	0.03 (0.02 to 0.04)	14816611 (10926460 to 19095362)	177.75 (131.51 to 228.8)	0.04 (0.03 to 0.05)
Sex									
Female	563873 (463688 to 680981)	14.42 (11.85 to 17.4)	−0.03 (−0.03 to −0.02)	11028999 (9134327 to 13015639)	263.62 (217.73 to 311.9)	0.02 (0.01 to 0.03)	6945825 (5104631 to 8940779)	166.4 (122.8 to 213.92)	0.03 (0.02 to 0.04)
Male	659348 (544531 to 791479)	16.42 (13.6 to 19.75)	−0.04 (−0.05 to −0.04)	12153110 (10069432 to 14367637)	291.61 (241.64 to 345)	0.04 (0.03 to 0.05)	7870786 (5820451 to 10164783)	188.96 (139.92 to 243.74)	0.05 (0.04 to 0.06)
SDI quintiles									
High SDI	152490 (126714 to 183180)	15.69 (12.95 to 18.76)	0.04 (0 to 0.08)	3837953 (3233343 to 4488357)	296.45 (247.61 to 349.3)	0 (−0.04 to 0.03)	2418425 (1798079 to 3069349)	188.61 (140.84 to 241.79)	−0.01 (−0.05 to 0.03)
High-middle SDI	201511 (172551 to 236757)	16.53 (14.15 to 19.33)	0.14 (0.12 to 0.15)	4484412 (3871242 to 5119820)	286.65 (246.57 to 329.25)	0.22 (0.2 to 0.24)	2879223 (2139705 to 3609614)	185.37 (138.35 to 233.14)	0.24 (0.22 to 0.26)
Middle SDI	391071 (321494 to 471393)	15.74 (13.01 to 18.96)	−0.11 (−0.12 to −0.1)	7591053 (6294522 to 8991069)	278.26 (230.98 to 330.63)	0.02 (0 to 0.03)	4875340 (3592613 to 6250839)	178.99 (132.63 to 229.93)	0.02 (0.01 to 0.03)
Low-middle SDI	313593 (251894 to 386296)	15.04 (12.21 to 18.29)	−0.01 (−0.02 to 0.01)	5127385 (4146444 to 6190285)	273.43 (224.02 to 328.22)	0.05 (0.03 to 0.07)	3273932 (2394047 to 4314385)	173.74 (127.97 to 227.77)	0.07 (0.05 to 0.09)
Low SDI	163692 (127545 to 203659)	14.54 (11.76 to 17.87)	−0.02 (−0.02 to −0.01)	2124992 (1699838 to 2616104)	243.92 (197.83 to 297.13)	0.03 (0.01 to 0.04)	1359274 (980588 to 1803203)	154.51 (112.4 to 202.64)	0.06 (0.04 to 0.07)
GBD regions									
Andean Latin America	9505 (7302 to 12140)	13.36 (10.31 to 16.96)	0.01 (0 to 0.02)	162743 (127620 to 205737)	239.92 (188.89 to 300.78)	0.05 (0.04 to 0.06)	104570 (73440 to 143715)	153.79 (107.21 to 210.87)	0.06 (0.05 to 0.07)
Australasia	5551 (4849 to 6305)	20.19 (17.87 to 22.74)	0.02 (0.01 to 0.02)	134948 (123734 to 146498)	387.08 (355.39 to 420.53)	0.01 (0.01 to 0.01)	85419 (64460 to 103992)	246.81 (186.31 to 300.92)	0.01 (0.01 to 0.02)
Caribbean	5992 (4651 to 7578)	12.36 (9.55 to 15.61)	−0.02 (−0.04 to −0.01)	112862 (89834 to 140947)	222.19 (176.38 to 278.19)	−0.01 (−0.02 to 0)	71833 (51417 to 97074)	141.64 (101.17 to 191.39)	−0.01 (−0.02 to 0)
Central Asia	12222 (9356 to 15540)	12.43 (9.55 to 15.74)	0 (−0.01 to 0)	216294 (167406 to 273703)	215.61 (167.53 to 271.96)	0.05 (0.03 to 0.07)	139158 (98003 to 188925)	138.46 (98.16 to 187.81)	0.06 (0.04 to 0.09)
Central Europe	12540 (10078 to 15505)	12.6 (9.97 to 15.82)	0.03 (0.02 to 0.03)	312508 (255244 to 382643)	224.46 (179 to 276.4)	0.08 (0.07 to 0.08)	197737 (140250 to 261296)	143.83 (102.58 to 192.2)	0.1 (0.09 to 0.11)
Central Latin America	36521 (29190 to 45125)	13.61 (10.88 to 16.81)	−0.02 (−0.03 to −0.01)	653292 (528794 to 797930)	243.71 (197.48 to 297.44)	0.01 (0 to 0.02)	416368 (300517 to 546017)	155.23 (112.09 to 203.52)	0.01 (0 to 0.02)
Central Sub-Saharan Africa	18469 (14120 to 23688)	13.79 (10.85 to 17.57)	−0.02 (−0.03 to −0.01)	223796 (169957 to 287507)	212.73 (165.35 to 266.97)	−0.03 (−0.06 to 0)	142343 (101461 to 194609)	134.01 (94.98 to 180.41)	0.02 (−0.01 to 0.05)
East Asia	244885 (209048 to 286292)	18.32 (15.77 to 21.17)	−0.04 (−0.07 to −0.01)	5498241 (4783147 to 6253122)	311.41 (270.11 to 356.3)	0.06 (0.04 to 0.09)	3559797 (2653372 to 4460257)	203.25 (151.83 to 255.72)	0.08 (0.06 to 0.11)
Eastern Europe	21245 (17519 to 25696)	11.73 (9.65 to 14.21)	0.23 (0.18 to 0.27)	513419 (429003 to 604368)	205.8 (169.71 to 245.81)	0.26 (0.2 to 0.31)	322427 (234985 to 411203)	130.6 (96.54 to 169.32)	0.28 (0.22 to 0.34)
Eastern Sub-Saharan Africa	60847 (47225 to 76143)	14.16 (11.46 to 17.53)	−0.02 (−0.03 to −0.02)	710915 (560238 to 886944)	216.9 (175.26 to 266.07)	0.05 (0.05 to 0.06)	455960 (328390 to 611301)	137.72 (99.56 to 182.66)	0.09 (0.08 to 0.11)
High-income Asia Pacific	22804 (18712 to 27723)	15.14 (12.25 to 18.55)	0.14 (0.06 to 0.22)	603909 (500972 to 713805)	271.65 (221.53 to 328.49)	0.05 (−0.02 to 0.12)	386429 (283591 to 497873)	176.16 (129.13 to 228.66)	0.06 (−0.01 to 0.13)
High-income North America	54817 (45915 to 65191)	16.68 (13.81 to 19.82)	−0.09 (−0.14 to −0.04)	1458582 (1252650 to 1685611)	347.65 (296.76 to 402.86)	−0.09 (−0.14 to −0.04)	904417 (677980 to 1130831)	217.61 (163.91 to 273.31)	−0.12 (−0.18 to −0.07)
North Africa and Middle East	93042 (73553 to 116456)	14.07 (11.05 to 17.66)	−0.04 (−0.04 to −0.03)	1574968 (1251997 to 1957684)	244.94 (195.26 to 301.81)	0.02 (0.02 to 0.03)	1005508 (723247 to 1352121)	155.75 (113.05 to 208.94)	0.02 (0.02 to 0.02)
Oceania	2473 (1912 to 3184)	17.05 (13.41 to 21.67)	0 (−0.02 to 0.01)	34436 (26450 to 44207)	274.77 (213.32 to 347.47)	−0.01 (−0.03 to 0)	22230 (15860 to 30696)	176 (124.92 to 242.09)	−0.01 (−0.02 to 0.01)
South Asia	314673 (253849 to 382452)	15.26 (12.45 to 18.51)	0.02 (0 to 0.03)	5536958 (4558070 to 6588275)	294.36 (243.49 to 348.6)	0.09 (0.07 to 0.11)	3525715 (2621574 to 4589423)	186.57 (138.37 to 241.76)	0.11 (0.09 to 0.13)
Southeast Asia	127997 (104674 to 155999)	17.34 (14.18 to 21.06)	0.07 (0.05 to 0.09)	2209595 (1791364 to 2672002)	290.41 (235.84 to 351.11)	0.16 (0.13 to 0.19)	1434796 (1038572 to 1879530)	188.39 (136.54 to 246.7)	0.18 (0.16 to 0.21)
Southern Latin America	10585 (8110 to 13526)	15.25 (11.64 to 19.37)	−0.01 (−0.02 to 0)	209688 (163480 to 263331)	278.76 (217.86 to 351.6)	0.01 (0 to 0.02)	133110 (93083 to 180219)	177.4 (123.82 to 240.29)	0.01 (0 to 0.02)
Southern Sub-Saharan Africa	12040 (9762 to 14602)	13.83 (11.29 to 16.73)	−0.01 (−0.02 to −0.01)	181544 (147510 to 220322)	224.7 (184.62 to 271.2)	0.03 (0.03 to 0.04)	113605 (84193 to 148079)	139.89 (103.86 to 182.73)	−0.02 (−0.03 to 0)
Tropical Latin America	32547 (26914 to 39419)	13.75 (11.35 to 16.6)	0 (−0.01 to 0.01)	624741 (517173 to 743686)	243.34 (201.46 to 290.42)	0.04 (0.03 to 0.05)	393258 (286664 to 504323)	153.38 (112.43 to 197.4)	0.05 (0.04 to 0.07)
Western Europe	53702 (45055 to 64511)	14.08 (11.57 to 16.97)	−0.02 (−0.04 to −0.01)	1346135 (1137843 to 1579942)	256.54 (213.26 to 306.09)	−0.03 (−0.05 to −0.01)	847987 (619730 to 1085060)	163.48 (121.07 to 212.63)	−0.02 (−0.05 to 0)
Western Sub-Saharan Africa	70761 (55513 to 87820)	14.95 (12.13 to 18.41)	−0.01 (−0.02 to −0.01)	862535 (686973 to 1070551)	237.21 (192.63 to 289.33)	0.08 (0.07 to 0.09)	553945 (396420 to 734735)	150.91 (108.56 to 198.24)	0.11 (0.1 to 0.12)

Data in parentheses are 95% uncertainty interval.

ASR, age-standardized rate; EAPC, estimated average percentage change; DALYs, disability-adjusted life years; SDI, Socio-Demographic Index; UI, uncertainty intervaL.

**Figure 1:**
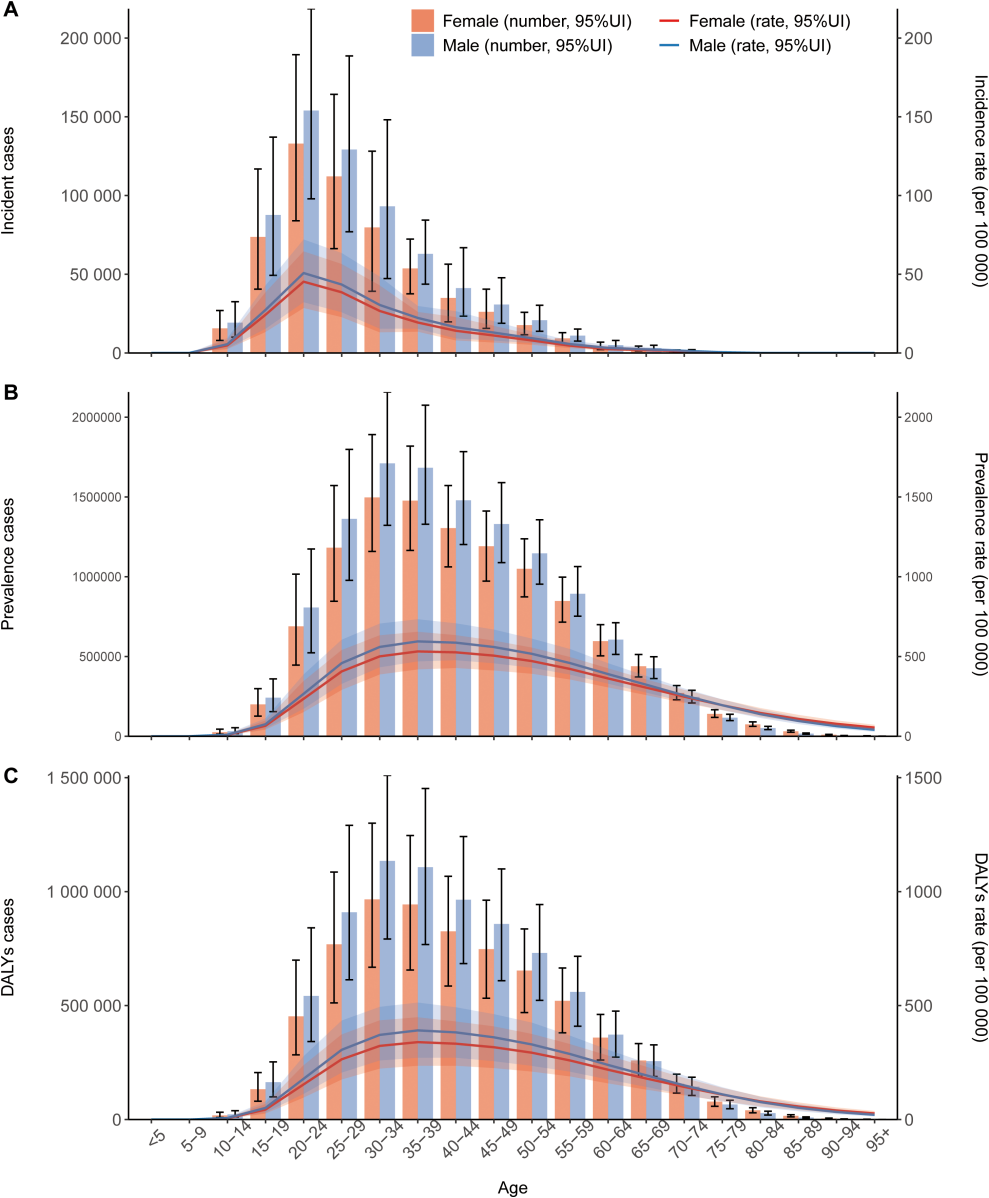
Global trends in counts and age-standardized rates of incidence **(A)**, prevalence **(B)**, and disability-adjusted life years (DALYs) **(C)** for schizophrenia by sex, 1990–2021. Error bars indicate the 95% uncertainty interval (UI) for counts. Shaded areas indicate the 95% UI for age-standardized rates.

**Figure 1** presents the age-stratified distribution of schizophrenia incident cases, prevalent cases, and DALYs by sex in 2021. The incidence rate of schizophrenia peaked in the 20–24 age group, with males consistently exhibiting higher rates than females across all age groups (**Figure 1A**). The number of prevalent cases of schizophrenia reached its maximum in the 30–34 age group, and males consistently showed a higher prevalence compared to females (**Figure 1B**). Similarly, DALYs attributed to schizophrenia peaked in the 30–34 age group, with males demonstrating consistently higher DALYs rate than females within the same age brackets (**Figure 1C**). Schizophrenia is characterized by a higher incidence among young adults and an elevated disease risk in males. Concurrently, the disease burden associated with schizophrenia is most pronounced in this young adult demographic.

**Figure 2:**
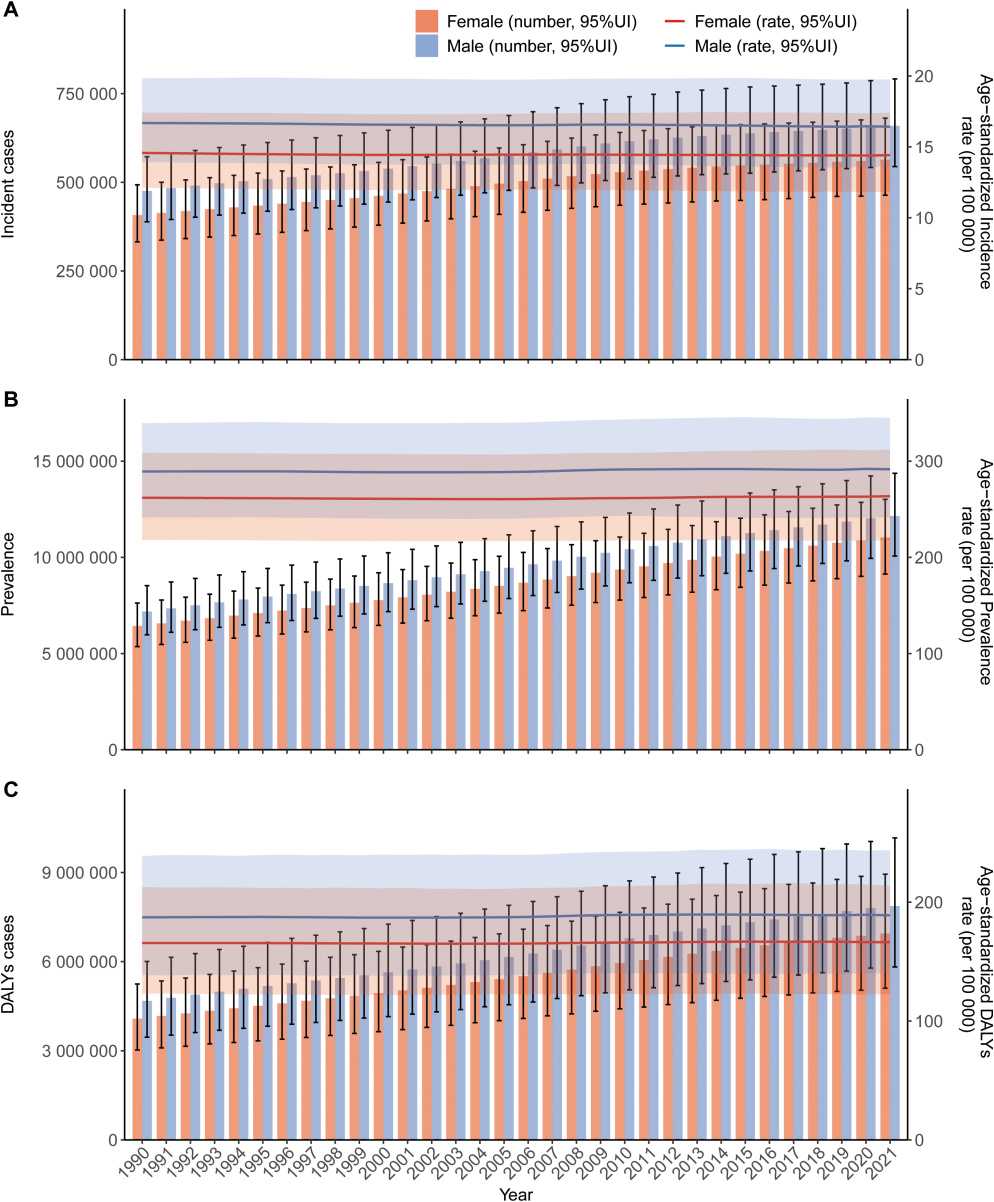
Age-specific trends in counts and rates of incidence **(A)**, prevalence **(B)**, and DALYs **(C)** for schizophrenia globally, 2021. Error bars indicate the 95% UI for counts. Shaded areas indicate the 95% UI for age-specific rates.

**Figure 2** illustrates the sex-stratified counts and rates of schizophrenia incidence, prevalence, and DALYs from 1990 to 2021. Data indicate that males consistently exhibited higher values than females across all metrics: incident cases, prevalent cases, DALY counts, and age-standardized incidence, prevalence, and DALY rates. In 2021, the ASIR, ASPR, and ASDR for males were 16.42 (13.6 to 19.75), 291.61 (241.64 to 345), and 188.96 (139.92 to 243.74) per 100, 000 population, respectively, all higher than the corresponding rates for females, which were 14.42 (11.85 to 17.4), 263.62 (217.73 to 311.9), and 166.4 (122.8 to 213.92) per 100, 000 population(**Table 1**). While the age-standardized rates of incidence, prevalence, and DALYs remained relatively stable over the study period, the absolute counts of incident cases, prevalent cases, and DALYs increased progressively year by year, a trend likely attributable to global population growth.

**Figure 3:**
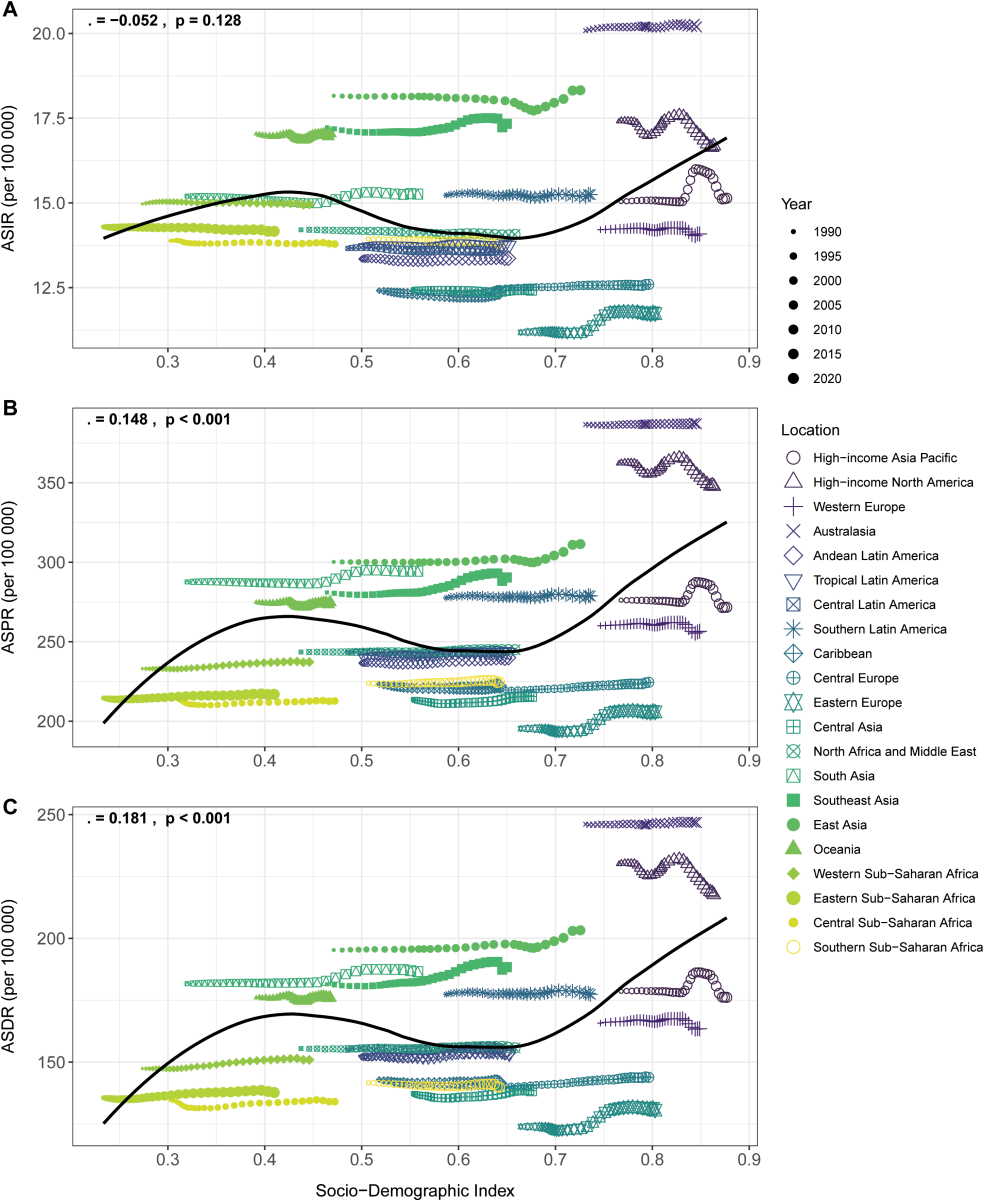
Trends in age-standardized incidence **(A)**, prevalence **(B)**, and DALYs **(C)** rates per 100, 000 population for schizophrenia in relation to Socio-demographic Index (SDI) and temporal variations across 21 GBD regions, 1990–2021.

In 2021, ASIR, ASPR, ASDR of schizophrenia tended to be higher in regions within higher SDI quintiles, while the inverse pattern was observed in lower SDI quintiles and the high-income Asia Pacific region (**Table 1**; **Figure 3**). From 1990 to 2021, the ASR of schizophrenia incidence, DALYs, and prevalence displayed stable trends at the lower of the SDI spectrum, whereas the largest increases and decreases occurred at the higher of the SDI spectrum. Among the 21 GBD regions in 2021, Australia exhibited the highest ASIR [20.19 (95% UI:17.87 to 22.74)], ASPR [387.08 (95% UI:355.39 to 420.53)]and DALY rate [246.81 (95% UI: 186.31 to 300.92], whereas Eastern Europe recorded the lowest values for these metrics [11.73 (9.65 to 14.21)], [205.8 (95% UI:169.71 to 245.81)] and[130.6 (95% UI:96.54 to 169.32.)]. From 1990 to 2021, Eastern Europe stood out with significant increases in ASIR, ASPR, and ASDR, with EAPC of 0.23 (95% UI:0.18 to 0.27), 0.26 (95% UI:0.2 to 0.31), and 0.28 (95% UI:0.22 to 0.34), respectively.

**Figure 4:**
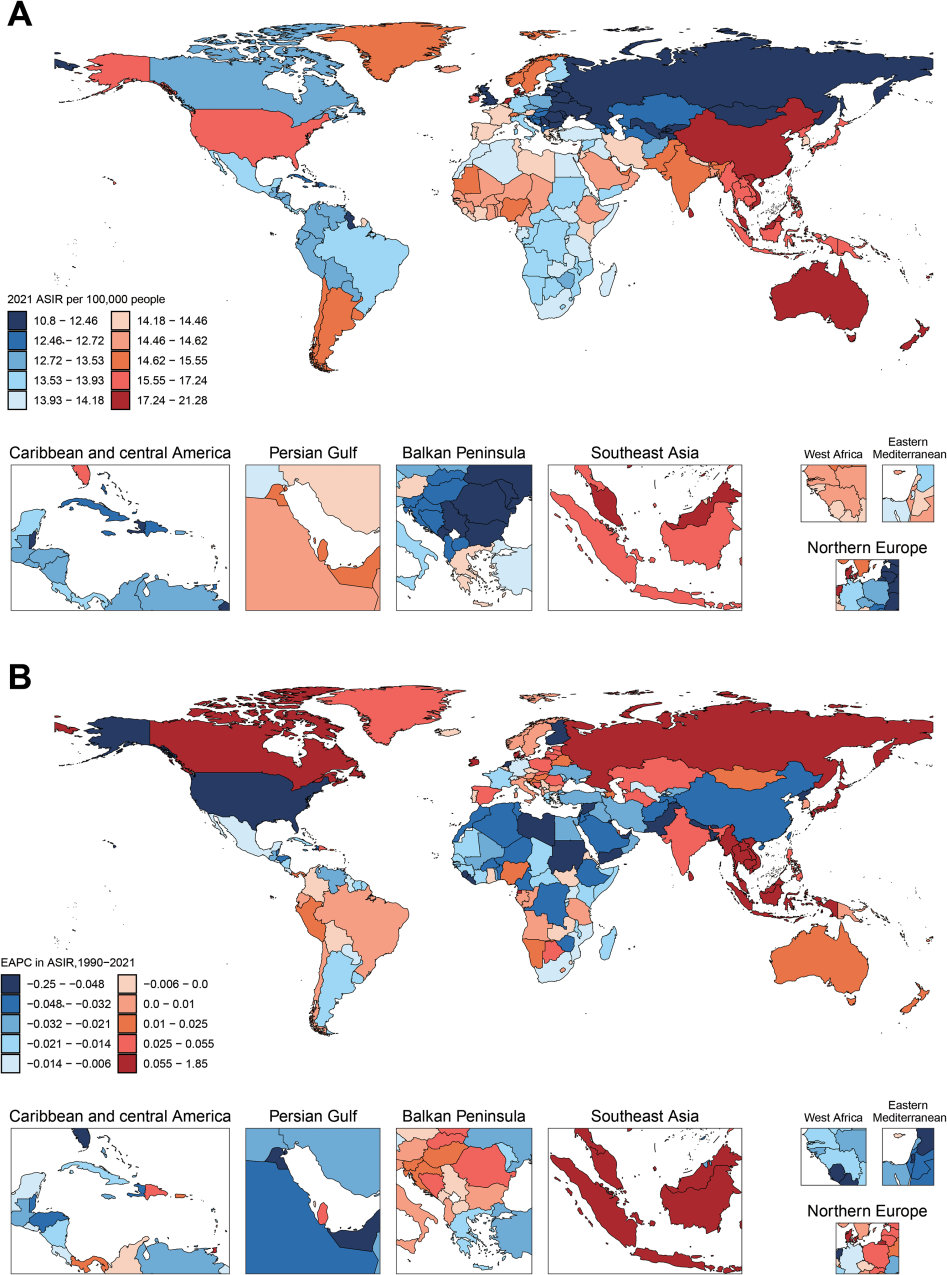
Global distribution in ASIR and trends in estimated annual percentage change (EAPC) of schizophrenia across 204 countries and territories for both sexes. **(A)** The ASIR per 100, 000 in 2021. **(B)** The EAPC of ASIR from 1990 to 2021.

In 2021, the ASR for schizophrenia exhibited clear geospatial disparities, with the highest ASIR, ASPR, and ASDR all found in developed countries and the lowest rates consistently located in less developed regions. **Figure 4A** displays the global distribution of ASIR in 2021. Regions with higher disease incidence, such as East Asia, are closer to red, while regions with lower incidence, such as Eastern Europe, are closer to blue Among all countries, Denmark had the highest age-standardized incidence rate in 2021 [21.28 (95% UI:19.39 to 22.28], whereas Suriname recorded the lowest ASIR of 11.7 (95% UI:8.78, 14.84) (**Supplementary Table S2**). In **Supplementary Figure S1A**, the global distribution of ASPR is illustrated. Somalia exhibited the lowest ASPR globally [196.06 (95% UI:152.11 to 248.66)], while Australia showed the highest ASPR [388.26 (95% UI:358.09 to 419.41)] (**Supplementary Table S3**). **Supplementary Figure S2A** presents the global ASDR distribution, which aligns with ASPR trends: Somalia and Australia displayed the lowest and highest ASDR values, respectively, at [95% UI:124.77 (88.99 to 168.39] and [95% UI:247.58 (186.2 to 300.2)] (**Supplementary Table S4**).

The 1990–2021 trends for schizophrenia also showed distinct geographical disparities. **Figures 4B**, **Supplementary Figures S1B**, and **S2B** map the EAPC of global ASIR, ASPR, and ASDR from 1990 to 2021. Cooler tones indicate pronounced downward trends, while warmer tones reflect increasing trends. Regional variations in color intensity highlight divergent epidemiological patterns. Across all three metrics, the United Kingdom showed the most significant decline: ASIR EAPC [−0.3352 (95% UI: −0.4728 to −0.1974)], ASPR EAPC: [−0.3275 (95% UI: −0.4629 to −0.1919)], and ASDR EAPC: [−1.772 (95% UI: −2.0963 to −1.4487)]. Conversely, Denmark exhibited the strongest upward trends: [ASIR EAPC: 1.772 (95% UI:1.4487 to 2.0963)], [ASPR EAPC: 1.7412 (95% UI:1.4207 to 2.0626)], and [ASDR EAPC: −0.3352 (95% UI: −0.4728 to −0.1974)].

### Joinpoint analysis

**Figure 5:**
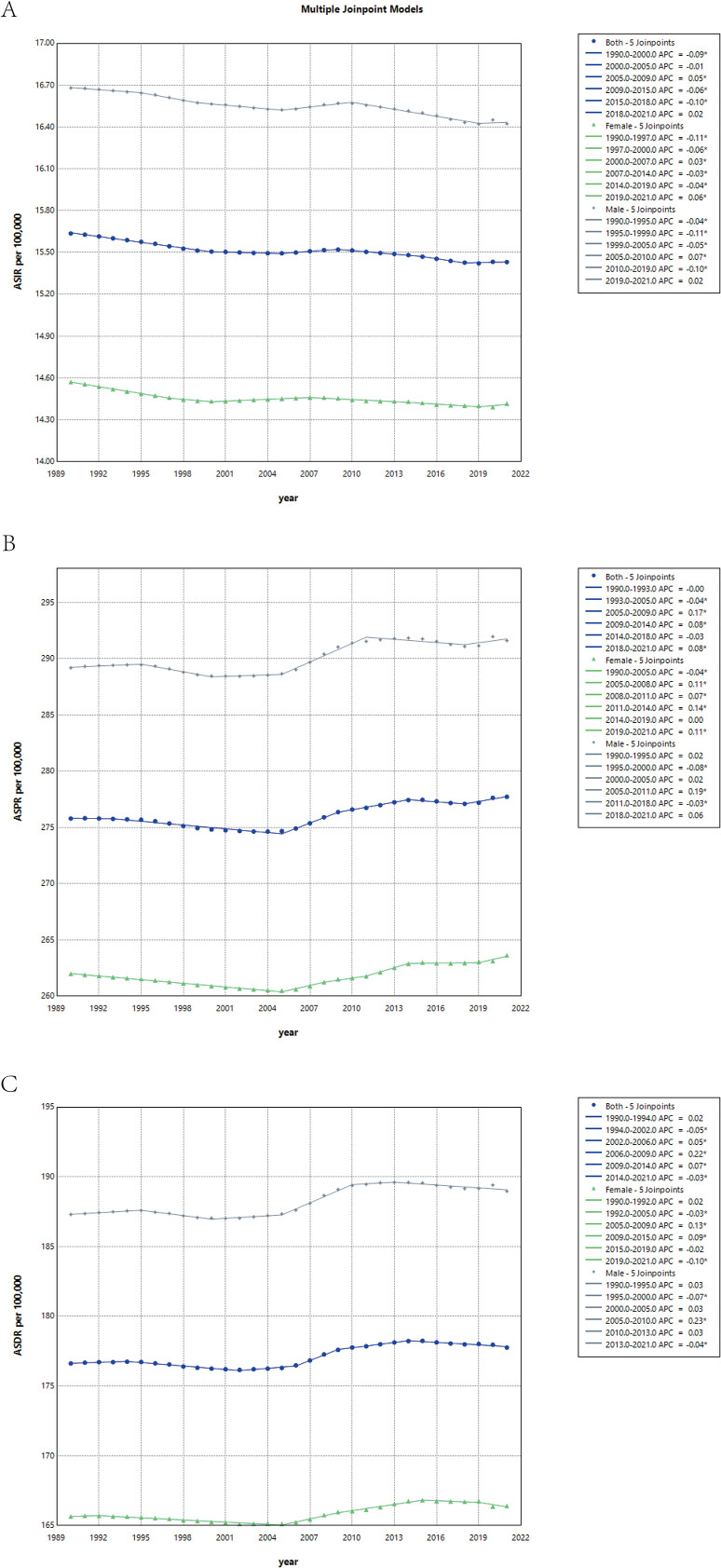
Joinpoint regression analysis of sex-specific age-standardized incidence **(A)**, prevalence **(B)** and DALYs **(C)** rates for schizophrenia in both sexes, 1990–2021.

For the ASIR, **Figure 5A** indicates an overall declining trend with intermittent fluctuations throughout the study period. Regarding the ASPR, **Figure 5B** illustrates a steady decrease from 1990 to 2004, followed by a gradual upward trajectory that stabilized after 2013.ASDR, as depicted in **Figure 5C**, exhibited a minor initial rise followed by a decline between 1990 and 2004, transitioning to a sustained upward trend before stabilizing into a gradual decline post 2014.

Notably, the temporal trends of ASIR, ASPR, and ASDR were broadly consistent across both sexes. However, males consistently demonstrated higher values across all three indicators, reflecting a greater disease burden of schizophrenia in male populations compared to females.

The detailed data is shown in **Supplementary Table S5**.

### Age, period, and cohort effects

**Supplementary Figure S3** demonstrates a significant age effect on disease incidence, with the highest rates observed among younger populations. The period effect indicates a gradual decline in risk over time, peaking in 1995. Birth cohort variations showed minimal differences, suggesting relatively stable cohort effects. Regarding disease prevalence, **Supplementary Figure S4** similarly exhibits notable age effects, with prevalence rates predominantly concentrated in middle-aged groups. The period effect reveals that relative risks remained relatively stable between 1995 and 2005, followed by a gradual increase in subsequent years. Cohort effects demonstrate a steady rise in risk for birth cohorts from 1940 to 1990, which subsequently plateaued. **Supplementary Figure S5** displays age, period, and cohort effects for DALYs that broadly align with those observed for prevalence patterns.

**Supplementary Figure S6** illustrates the relative risks (RRs) of age, period, and birth cohort effects on schizophrenia incidence, prevalence, and DALYs, calculated using the intrinsic estimator (IE) model. The analysis indicates that the relative risk for age effects initially increased and then decreased with advancing age. Period effects remained relatively stable, with minor fluctuations in incidence rates and a gradual rise in DALYs. Cohort effects revealed a significant upward trend in relative risks for incidence prior to the 1967 birth cohort, followed by a sudden decline and subsequent plateau in later cohorts. For prevalence, slight variations in relative risks were observed across birth cohorts. The relative risks for DALYs showed a slow increase before the 1967 birth cohort, stabilizing thereafter.

### Decomposition analysis

**Supplementary Figure S7A** highlight that the observed increase in schizophrenia incidence within the general population over the past three decades is primarily attributable to population growth and epidemiological changes, with demographic expansion playing a more pronounced role. **Supplementary Figures S7B, C** further emphasize that population growth served as the predominant driver of rising prevalence and DALYs, with consistent patterns observed across both male and female subgroups.

### Frontier analysis

**Supplementary Figure S8A** displays the ASR of **s**chizophrenia across countries from 1990 to 2021 plotted against the SDI. The frontier line represents the SDI-based expected ASR, serving as a comparative benchmark. As SDI increases, the ASR generally rises and exhibits greater dispersion. Notably, once SDI exceeds 0.5, the frontier trends become markedly more scattered, indicating heightened variability in ASR values among countries above this threshold.

**Supplementary Figure S8B** highlights the gap between countries observed disease burden (based on its SDI) and its potential achievable disease burden. Our analysis reveals that the largest effective disparities predominantly occur in high-SDI countries such as Australia, New Zealand, and Denmark, where observed values deviate substantially from the frontier line. Conversely, countries like Somalia and the Central African Republic demonstrate minimal effective disparities. This pattern underscores that high-SDI countries continue to face significant public health challenges despite their advanced socioeconomic development. However, the low effective disparity observed in low-SDI regions may also stem from insufficient disease awareness and a lack of diagnostic tools.

### Health inequality analysis

Between 1990 and 2021, the global disparity in DALYs due to schizophrenia showed a narrowing trend. However, health inequalities driven by economic development disparities persist. The Slope Index of Inequality (SII), quantifying absolute health inequality, revealed that the gap in DALYs between the highest- and lowest-income countries decreased from 85.77(95% UI:74.83 to 96.71) DALYs per 100, 000 population in 1990 to −21.5 (95% UI: −38.01 to −5.00) in DALYs per 100, 000 population 2021. It shows that global health inequalities have decreased. But this reversal in SII values indicates a shift in disease burden patterns: from increasing with rising economies to decreasing with rising economies. (**Supplementary Figure S9A**). The Relative Concentration Index (RCI), assessing the socioeconomic concentration of health inequality, increased from 0.002 in 1990 to 0.168 in 2021. This upward trend highlights a growing concentration of health advantages among high socioeconomic groups over time (**Supplementary Figure S9B**).

### Prediction from 2022 to 2036

**Figure 6:**
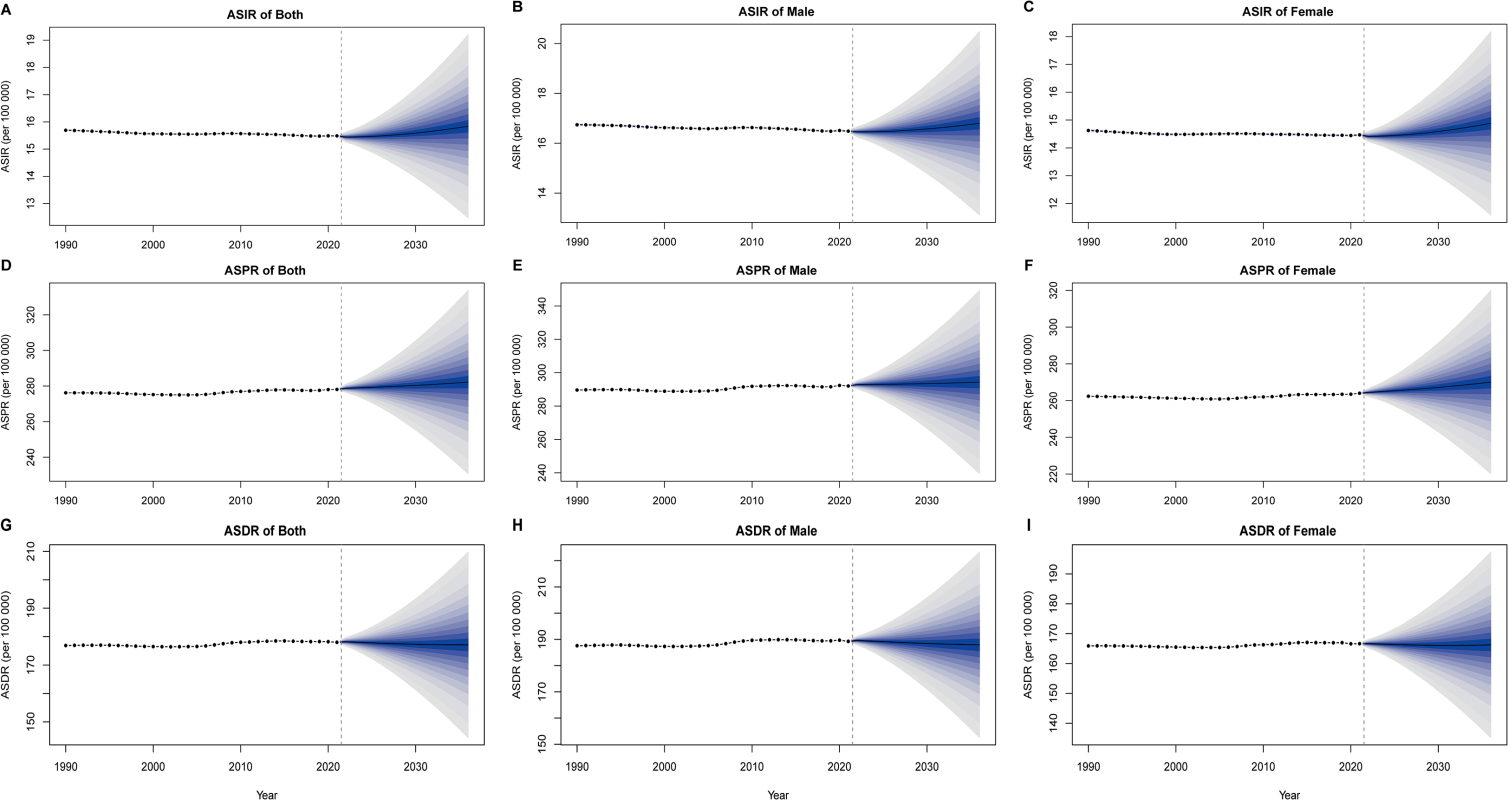
Bayesian age-period-cohort (BAPC) model sex-specific predictions of ASIR, ASPR, and ASDR. **(A)** ASIR of both. **(B)** ASIR of male. **(C)** ASIR of female **(D)** ASPR of both. **(E)** ASPR of male. **(F)** ASPR of female **(G)** ASDR of both. **(H)** ASDR of male. **(I)** ASDR of female. The shaded areas represent the 95% confidence intervals.

**Figure 6** presents BAPC model projections of ASIR, ASPR, and ASDR for male, female, and total populations from 2022 to 2036. The results highlight two distinct patterns: While the ASPR trend continues its upward trajectory accompanied by expanding variability in future estimates, both ASDR and ASPR demonstrate relative stability up to 2020 followed by progressively increasing uncertainty in subsequent projections.

**Figure 7:**
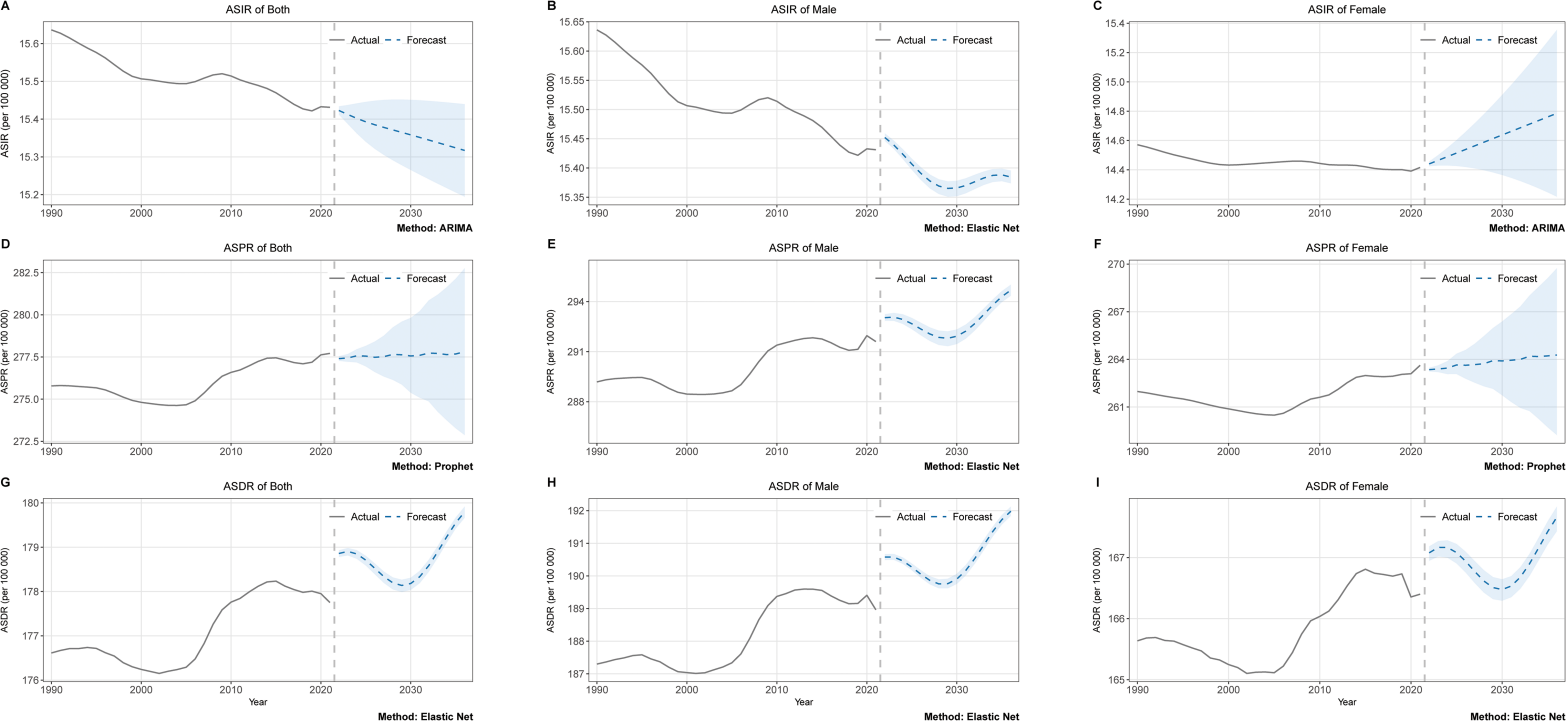
Machine learning sex-specific predictions of ASIR, ASPR, and ASDR. **(A)** ASIR of both. **(B)** ASIR of male. **(C)** ASIR of female **(D)** ASPR of both. **(E)** ASPR of male. **(F)** ASPR of female **(G)** ASDR of both. **(H)** ASDR of male. **(I)** ASDR of female. The shaded areas represent the 95% confidence intervals.

**Figure 7** shows the statistical models and machine learning models that has the best performance after being selected through rolling cross-validation. For ASIR of the general population, the ARIMA model demonstrated optimal performance. Projections based on this model suggest a continued global decline in schizophrenia ASIR over the next 15 years (**Figure 7A**). The Elastic Net model achieved superior predictive accuracy for male ASIR, indicating a similar downward trajectory that gradually stabilizes in later years (**Figure 7B**). Conversely, the ARIMA model emerged as the best performer for female population ASIR, forecasting an upward trend contrary to the global pattern (**Figure 7C**). Regarding ASPR, the Prophet model exhibited the strongest predictive capability for the global population, projecting a slight increasing trend over the forecast period (**Figure 7D**). For male ASPR, the Elastic Net model showed optimal performance, predicting a fluctuating pattern characterized by initial decline followed by sustained growth (**Figure 7E**). Similarly, the Prophet model proved most effective for female ASPR prediction, also indicating a progressive upward trend (**Figure 7F**). In ASDR predictions across genders, the Elastic Net model consistently outperformed other approaches (**Figure 7G**). Both male and female populations displayed comparable forecasting trajectories featuring transitional declines followed by gradual increases, ultimately maintaining an overall upward trend (**Figures 7H, I**).

## Discussion

Schizophrenia remains a significant global health burden. In 2021, there were over 20 million individuals living with schizophrenia worldwide, including over 1.2 million new cases, contributing to approximately 14, 811, 611 DALYs. Over the past 31 years, the ASIR of schizophrenia has shown a declining trend, with an EAPC of 0.04%. In contrast, the ASPR and ASDR exhibited notable increases, with EAPCs of 0.03% and 0.04%, respectively. These trends highlight the prolonged disease course of schizophrenia; despite reduced incidence, the growing patient population and associated disease burden underscore the urgent need for enhanced healthcare interventions and preventive strategies. Merely controlling incidence rates is insufficient to alleviate the societal impact of this disorder. Public health strategies need to transition from a sole focus on prevention to integrating it with long-term patient management, so as to effectively reduce the societal impact of schizophrenia.

Consistent with findings from the GBD 2019, our analysis revealed significantly higher ASIR, ASPR, and ASDR among males compared to females (7). Previous studies suggest that males face a higher likelihood of developing schizophrenia and experience poorer intermediate-term prognoses, potentially linked to higher rates of substance abuse and sex-based differences in brain morphology (28, 29). These findings emphasize the necessity of prioritizing prevention and treatment strategies for male patients. Additionally, prior research has established that schizophrenia typically manifests in late adolescence or early adulthood (around age 25), with slightly later onset in females. Our age-specific analysis aligns with these observations. This critical period of brain development during late adolescence may heighten susceptibility to psychopathological disturbances (30, 31). Notably, while the peak incidence occurs in late adolescence, the highest prevalence and DALY rates are observed in middle-aged populations, reflecting the chronic nature of schizophrenia. Consequently, healthcare systems must prioritize long-term care and support for middle-aged individuals to mitigate the enduring burden of this disease. Although the age-standardized incidence, prevalence, and DALY rates across different genders remain relatively stable, the absolute numbers of incident cases, prevalent cases, and DALYs have been increasing year by year. The primary reason for this trend is global population growth. This also signals the significant challenges that global population growth poses to public health worldwide.

Compared to 1990, the ASIR of schizophrenia exhibited a declining trend globally in 2021, whereas the ASPR and ASDR showed increases. This indicates that despite reduced relative incidence, the overall disease burden of schizophrenia remains unmitigated worldwide. In the analysis stratified by SDI regions, trends in ASIR, ASPR, and ASDR aligned with prior findings (32, 33). The distribution of ASIR across SDI quintiles: the highest rates observed in middle and high-middle SDI regions, while high and low SDI regions reported relatively lower rates. This suggests that schizophrenia incidence is not linearly correlated with economic development. Similarly, trends in ASIR, with the most pronounced increases observed in high-middle SDI quintiles, modest growth in high SDI quintiles, and declines in other quintiles. Contributing factors of trends may include rapid urbanization and heightened social stress in high-middle and middle SDI quintiles, as well as higher rates of substance abuse in high SDI quintiles, a known risk factor for schizophrenia (12, 34). In contrast, ASPR and ASDR demonstrated a clear positive association with SDI levels, increasing with higher SDI. However, temporal trends diverged: high SDI quintiles exhibited declining ASPR and ASDR, while other quintiles showed rising burdens. This disparity may reflect the greater availability of healthcare resources in higher SDI quintiles, which prolong survival but are unable to achieve complete cure, thereby maintaining elevated prevalence and disability rates. Nevertheless, with sufficient healthcare resources and advanced treatment protocols that have proven effective, high-SDI quintiles are the only ones where both prevalence rates and disease burden have demonstrated a downward trend. Therefore, promoting the global adoption of management and treatment strategies from developed nations should be the next priority. The demonstrated success in high-SDI regions provides a viable roadmap. The global priority must be to adapt and promote the adoption of these proven management and treatment strategies.

In the analysis of specific countries and regions, the highest ASIR were observed in Australasia and East Asia, primarily driven by contributions from China and Australia. These countries share a notable characteristic of relatively rapid population growth (35). Therefore, the increase in ASIR may be attributed to population growth. In contrast, Eastern Europe exhibited the lowest ASIR values, with countries such as Moldova, Belarus, Ukraine, and neighboring Russia ranking among the bottom ten globally. A The majority of these countries have experienced negative population growth, which may be an important factor contributing to the decline in ASIR. In the comparison of countries, Denmark exhibited the highest ASIR with marked growth, possibly associated with environmental factors or substance abuse (36). The United Kingdom and the United States displayed significant declines in ASIR, likely attributable to sustained investments in health infrastructure (37, 38). Regarding ASPR and ASDR, the top three regions were Australasia, high-income North America, and East Asia, predominantly influenced by nations such as Australia, New Zealand, the United States, and China. These countries’robust economic development and well-established healthcare infrastructure facilitate extended lifespans for schizophrenia patients, thereby elevating ASPR and ASDR values. Concurrently, These regions’ healthcare systems contribute to stabilizing or reducing trends in ASIR and ASDR. Conversely, low ASPR and ASDR in Eastern Europe and parts of Africa (e.g., Moldova, Suriname, Central African Republic) likely stem from underdeveloped healthcare systems hindered by poverty and conflict, leading to underdiagnosis (39–41). Despite this, rising trends in these regions may reflect gradual healthcare improvements or international aid. Notably, in addition to its high ASIR, Denmark is experiencing persistent increases in both ASPR and ASDR. This trend collectively signals fundamental shortcomings in the country’s approach to disease prevention, management, and treatment, underscoring an urgent need for targeted health policy reforms.

Our BAPC prediction models indicate that the global burden of schizophrenia will maintain a stable trajectory through 2036. Specifically, the ASPR and ASDR are projected to remain largely unchanged from current levels. This stability aligns with other BAPC prediction results, which also suggest that the ASPR and ASDR of the disease will remain relatively stable worldwide (11). Furthermore, our models project that the ASIR will also maintain relative stability at the global aggregate level. Collectively, the BAPC findings suggest that the overall incidence and disease burden of schizophrenia are unlikely to undergo significant changes by 2036.However, time series forecasting models reveal distinct directional patterns. The ASIR of schizophrenia demonstrates an overall decline, though notable gender disparities emerge: male ASIR trends downward, while female ASIR exhibits an upward trajectory. This divergence not only indicates female-dominated trends in epidemiological changes but also underscores the imperative to prioritize preventive measures targeting schizophrenia risk factors in women. Both the ASPR and ASDR exhibit fluctuating upward trends. This pattern was also observed in previous predictions using conventional ARIMA models, and our application of multiple machine learning algorithms further substantiates its likelihood (10). The projected continued growth in the disease burden of schizophrenia indicates that its treatment and management systems face severe challenges. Our predictions also have certain limitations. Firstly, the forecasts primarily rely on historical data, making it difficult to predict emerging risk factors or policy changes, while long-term predictions entail greater uncertainty. Secondly, although machine learning models often achieve high accuracy, their decision-making processes remain opaque, and it is challenging to explain the relationship between predictions and specific influencing factors. Nevertheless, these predictions can still outline potential future trends, provide references for policy formulation, and serve as a baseline for assessing the impact of unexpected events.

Decomposition analysis identifies population growth and aging demographics as primary drivers of schizophrenia-related disease burden. With global population expansion and accelerated aging projected to intensify, these findings necessitate worldwide preparedness for escalating healthcare demands and the establishment of more comprehensive medical systems. The economic implications of schizophrenia burden manifest across both developed and underdeveloped regions. Health inequality analyses reveal a growing concentration of health advantages in high-SDI regions, exposing systemic deficiencies in healthcare infrastructure within economically disadvantaged areas. These regions urgently require enhanced schizophrenia treatment and management frameworks. Frontier analysis further indicates that many developed nations underperform relative to projected disease burden, highlighting persistent gaps in preventive and therapeutic interventions.

This study systematically analyzed the global counts and ASR of incidence, prevalence, and DALYs of schizophrenia in 2021, evaluated trends over the past 32 years, and projected trajectories for the next 15 years. These findings provide critical evidence to inform public health strategies and policies for addressing the anticipated growth in schizophrenia-related disease burden. However, several limitations should be acknowledged. First, the primary data were derived from the GBD 2021 database, which may contain incomplete statistical coverage, particularly in remote regions with limited sample sizes, potentially compromising the reliability of results. Second, while machine learning models were employed for trend prediction, key socioeconomic, political, and environmental determinants were not incorporated into the forecasting framework, which may affect prediction accuracy. Third, the comparative analysis of different projection methodologies lacks a unified reference standard, necessitating cautious interpretation.

## Conclusion

Our research demonstrates that schizophrenia’s ASIR, ASPR, and ASDR exhibit significant correlations with gender, SDI, and regions. Despite the observed downward trend in ASIR over the past 32 years and projected continuation through 2036, ASPR and ASDR maintain upward trajectories. The escalating disease burden of schizophrenia poses substantial challenges to both economically developed and underdeveloped regions. Proactive governmental interventions are imperative: high-income regions should prioritize the development of sophisticated prevention and management systems, while economically disadvantaged areas require enhanced disease awareness campaigns and strengthened basic treatment infrastructure. These stratified strategies highlight the need for context-specific solutions to mitigate the growing global impact of schizophrenia.

## Data Availability

Publicly available datasets were analyzed in this study. This data can be found here: Relevant data can be retrieved through the available website (https://vizhub.healthdata.org/gbd-results/).

## Supplementary material

Supplementary Figure 1 Global distribution in ASPR and trends in EAPC of schizophrenia across 204 countries and territories for both sexes. **(A)** The ASPR per 100, 000 in 2021. **(B)** The EAPC of ASPR from 1990 to 2021.

Supplementary Figure 2 Global distribution in ASDR and trends in EAPC of schizophrenia across 204 countries and territories for both sexes. **(A)** The ASDR per 100, 000 in 2021. **(B)** The EAPC of ASDR from 1990 to 2021.

Supplementary Figure 3 Age, period and cohort effects on incidence of schizophrenia.

Supplementary Figure 4 Age, period and cohort effects on prevalence of schizophrenia.

Supplementary Figure 5 Age, period and cohort effects on DALYs of schizophrenia.

Supplementary Figure 6 The age, period, and birth cohort effects on the relative risks (RRs) for incidence, prevalence, and DALYs of schizophrenia. **(A)** incidence. **(B)** prevalence. C, DALYs.

Supplementary Figure 7 Decomposition analysis stratified by sex of incidence, prevalence, and DALYs of schizophrenia. **(A)** incidence. **(B)** prevalence. **(C)** DALYs.

Supplementary Figure 8 Frontier analysis across 204 countries and territories based on the Socio-demographic Index (SDI) and the age-standardized disability-adjusted life years (DALYs) rate of schizophrenia in 2021.

Supplementary Figure 9 Health inequality analysis of incidence, prevalence, and DALY rates from 1990 to 2021. **(A)** slope index analysis of inequality on DALY rates. **(B)** concentration index analysis on DALY rates.

Supplementary Table 1 Performance evaluation results of the optimal statistical models and machine learning models selected through rolling cross-validation.

Supplementary Table 2 The cases of schizophrenia and ASIR of schizophrenia in 1990 and 2021, and its temporal trends from 1990–2021 among 204 countries and territories.

Supplementary Table 3 The cases of prevalence and ASPR of schizophrenia in 1990 and 2021, and its temporal trends from 1990–2021 among 204 countries and territories.

Supplementary Table 4 The cases of DALYs and ASDR of schizophrenia in 1990 and 2021, and its temporal trends from 1990–2021 among 204 countries and territories.

Supplementary Table 5 Joinpoint regression analysis: trends in age-standardized incidence, prevalence, DALYs (per 100, 000 persons) among both sexes, males, and females, 1990–2021.

## Glossary

GBD: global burden of disease
EAPC: estimated annual percentage change
UI: uncertainty interval
BAPC: Bayesian age-period-cohort
DALYs: disability-adjusted life-years
ASIR: age-standardized incidence rate
ASPR: age-standardized prevalence rate
ASDR: age-standardized disability-adjusted life years rate
SDI: socio-demographic index
ASR: age-standardized rate
AAPC: average annual percent change
APC: annual percentage change
ARIMA: autoregressive integrated moving average
GLMNET: elastic net generalized linear model
XGBoost: extreme gradient boosting
MAE: Mean Absolute Error
MAPE: Mean Absolute Percentage Error
MARS: multivariate adaptive regression splines
MASE: Mean Absolute Scaled Error
MSE: Mean Squared Error
RMSE: Root Mean Squared Error
R²: R-squared

## References

[1] van Os J Kapur S . Schizophrenia. Lancet. (2009) 374:635–45. doi: 10.1016/S0140-6736(09)60995-8, PMID: 19700006

[2] Owen MJ Sawa A Mortensen PB . Schizophrenia. Lancet. (2016) 388:86–97. doi: 10.1016/S0140-6736(15)01121-6, PMID: 26777917 PMC4940219

[3] Laursen TM Nordentoft M Mortensen PB . Excess early mortality in schizophrenia. Annu Rev Clin Psychol. (2014) 10:425–48. doi: 10.1146/annurev-clinpsy-032813-153657, PMID: 24313570

[4] Janoutová J Janácková P Serý O Zeman T Ambroz P Kovalová M . Epidemiology and risk factors of schizophrenia. Neuro Endocrinol Lett. (2016) 37:1–8. 26994378

[5] GBD 2021 Diseases and Injuries Collaborators . Global incidence, prevalence, years lived with disability (YLDs), disability-adjusted life-years (DALYs), and healthy life expectancy (HALE) for 371 diseases and injuries in 204 countries and territories and 811 subnational locations, 1990-2021: a systematic analysis for the Global Burden of Disease Study 2021. Lancet. (2024) 403:2133–61. doi: 10.1016/S0140-6736(24)00757-8, PMID: 38642570 PMC11122111

[6] McCutcheon RA Reis Marques T Howes OD . Schizophrenia-an overview. JAMA Psychiatry. (2020) 77:201–10. doi: 10.1001/jamapsychiatry.2019.3360, PMID: 31664453

[7] Solmi M Seitidis G Mavridis D Correll CU Dragioti E Guimond S . Incidence, prevalence, and global burden of schizophrenia - data, with critical appraisal, from the Global Burden of Disease (GBD) 2019. Mol Psychiatry. (2023) 28:5319–27. doi: 10.1038/s41380-023-02138-4, PMID: 37500825

[8] Häfner H an der Heiden W . Epidemiology of schizophrenia. Can J Psychiatry. (1997) 42:139–51. doi: 10.1177/070674379704200204, PMID: 9067063

[9] Lin C Zhang X Jin H . The societal cost of schizophrenia: an updated systematic review of cost-of-illness studies. Pharmacoeconomics. (2023) 41:139–53. doi: 10.1007/s40273-022-01217-8, PMID: 36404364

[10] Zhan Z Wang J Shen T . Results of the Global Burden of Disease study for schizophrenia: trends from 1990 to 2021 and projections to 2050. Front Psychiatry. (2025) 16:1629032. doi: 10.3389/fpsyt.2025.1629032, PMID: 40980052 PMC12447577

[11] Huo J Li R Ren X Zhu S Hu X Tan Q . Trends in incidence, prevalence, and disability-adjusted life years of schizophrenia in China from 1990 to 2021, with projections for 2022-2050. Front Psychiatry. (2025) 16:1651350. doi: 10.3389/fpsyt.2025.1651350, PMID: 40980046 PMC12443762

[12] Zhang S Qi X Wang Y Fang K . Global burden of drug use disorders by region and country, 1990-2021. Front Public Health. (2024) 12:1470809. doi: 10.3389/fpubh.2024.1470809, PMID: 39534741 PMC11554507

[13] Li W Law K . Deep learning models for time series forecasting: A review. IEEE Access. (2024) 12:1–1. doi: 10.1109/ACCESS.2024.3422528

[14] Chen S Chen M Wu X Lin S Tao C Cao H . Global, regional and national burden of low back pain 1990-2019: A systematic analysis of the Global Burden of Disease study 2019. J Orthop Translat. (2022) 32:49–58. doi: 10.1016/j.jot.2021.07.005, PMID: 34934626 PMC8639804

[15] DeSantis C Siegel R Bandi P Jemal A . Breast cancer statistics, 2011. CA Cancer J Clin. (2011) 61:409–18. doi: 10.3322/caac.20134, PMID: 21969133

[16] Cao G Liu J Liu M . Global, regional, and national incidence and mortality of neonatal preterm birth, 1990-2019. JAMA Pediatr. (2022) 176:787–96. doi: 10.1001/jamapediatrics.2022.1622, PMID: 35639401 PMC9157382

[17] Wang F Ma B Ma Q Liu X . Global, regional, and national burden of inguinal, femoral, and abdominal hernias: a systematic analysis of prevalence, incidence, deaths, and DALYs with projections to 2030. Int J Surg. (2024) 110:1951–67. doi: 10.1097/JS9.0000000000001071, PMID: 38265437 PMC11020045

[18] Li Y Ning Y Shen B Shi Y Song N Fang Y . Temporal trends in prevalence and mortality for chronic kidney disease in China from 1990 to 2019: an analysis of the Global Burden of Disease Study 2019. Clin Kidney J. (2023) 16:312–21. doi: 10.1093/ckj/sfac218, PMID: 36755850 PMC9900593

[19] Lei S Chen L Ji P Li K Li Q Huang C . Global burdens of nasopharyngeal carcinoma in children and young adults and predictions to 2040. Oral Oncol. (2024) 155:106891. doi: 10.1016/j.oraloncology.2024.106891, PMID: 38878356

[20] Xie Y Bowe B Mokdad AH Xian H Yan Y Li T . Analysis of the Global Burden of Disease study highlights the global, regional, and national trends of chronic kidney disease epidemiology from 1990 to 2016. Kidney Int. (2018) 94:567–81. doi: 10.1016/j.kint.2018.04.011, PMID: 30078514

[21] Knoll M Furkel J Debus J Abdollahi A Karch A Stock C . An R package for an integrated evaluation of statistical approaches to cancer incidence projection. BMC Med Res Methodol. (2020) 20:257. doi: 10.1186/s12874-020-01133-5, PMID: 33059585 PMC7559591

[22] Zhai M Jiang Q Liu S Long J Zhang D Ren C . DALY trend and predictive analysis of COPD in China and its provinces: Findings from the global burden of disease study. Front Public Health. (2022) 10:1046773. doi: 10.3389/fpubh.2022.1046773, PMID: 36620296 PMC9816410

[23] Taylor SJ Letham B . Forecasting at scale. Am Statistician. (2018) 72:37–45. doi: 10.1080/00031305.2017.1380080

[24] Jerome HF . Multivariate adaptive regression splines. Ann Stat. (1991) 19:1–67. doi: 10.1214/aos/1176347963

[25] Esmaeili A Karamoozian A Bahrampour A . Mortality prediction in patients with breast cancer by artificial neural network model and elastic net regression. J Res Health Sci. (2025) 25:e00638. doi: 10.34172/jrhs.2025, PMID: 39996347 PMC11833499

[26] Gregory GA Robinson TIG Linklater SE Wang F Colagiuri S de Beaufort C . Global incidence, prevalence, and mortality of type 1 diabetes in 2021 with projection to 2040: a modelling study. Lancet Diabetes Endocrinol. (2022) 10:741–60. doi: 10.1016/S2213-8587(22)00218-2, PMID: 36113507

[27] Xia F Li Q Luo X Wu J . Machine learning model for depression based on heavy metals among aging people: A study with National Health and Nutrition Examination Survey 2017-2018. Front Public Health. (2022) 10:939758. doi: 10.3389/fpubh.2022.939758, PMID: 35991018 PMC9386350

[28] McGrath J Saha S Chant D Welham J . Schizophrenia: a concise overview of incidence, prevalence, and mortality. Epidemiol Rev. (2008) 30:67–76. doi: 10.1093/epirev/mxn001, PMID: 18480098

[29] Abel KM Drake R Goldstein JM . Sex differences in schizophrenia. Int Rev Psychiatry. (2010) 22:417–28. doi: 10.3109/09540261.2010.515205, PMID: 21047156

[30] Welham J Isohanni M Jones P McGrath J . The antecedents of schizophrenia: a review of birth cohort studies. Schizophr Bull. (2009) 35:603–23. doi: 10.1093/schbul/sbn084, PMID: 18658128 PMC2669575

[31] Gogtay N Vyas NS Testa R Wood SJ Pantelis C . Age of onset of schizophrenia: perspectives from structural neuroimaging studies. Schizophr Bull. (2011) 37:504–13. doi: 10.1093/schbul/sbr030, PMID: 21505117 PMC3080674

[32] He H Liu Q Li N Guo L Gao F Bai L . Trends in the incidence and DALYs of schizophrenia at the global, regional and national levels: results from the Global Burden of Disease Study 2017. Epidemiol Psychiatr Sci. (2020) 29:e91. doi: 10.1017/S2045796019000891, PMID: 31928566 PMC7214712

[33] Li N Chen S Wu Z Dong J Wang J Lei Y . Secular trends in the prevalence of schizophrenia among different age, period and cohort groups between 1990 and 2019. Asian J Psychiatr. (2024) 101:104192. doi: 10.1016/j.ajp.2024.104192, PMID: 39232389

[34] Colodro-Conde L Couvy-Duchesne B Zhu G Coventry WL Byrne EM Gordon S . A direct test of the diathesis-stress model for depression. Mol Psychiatry. (2018) 23:1590–6. doi: 10.1038/mp.2017.130, PMID: 28696435 PMC5764823

[35] Vollset SE Goren E Yuan CW Cao J Smith AE Hsiao T . Fertility, mortality, migration, and population scenarios for 195 countries and territories from 2017 to 2100: a forecasting analysis for the Global Burden of Disease Study. Lancet. (2020) 396:1285–306. doi: 10.1016/S0140-6736(20)30677-2, PMID: 32679112 PMC7561721

[36] Hjorthøj C Posselt CM Nordentoft M . Development over time of the population-attributable risk fraction for cannabis use disorder in schizophrenia in Denmark. JAMA Psychiatry. (2021) 78:1013–9. doi: 10.1001/jamapsychiatry.2021.1471, PMID: 34287621 PMC8295899

[37] Becker T Kilian R . Psychiatric services for people with severe mental illness across western Europe: what can be generalized from current knowledge about differences in provision, costs and outcomes of mental health care? Acta Psychiatr Scand Suppl. (2006) 12:9–16. doi: 10.1111/j.1600-0447.2005.00711.x, PMID: 16445476

[38] Stone WS Cai B Liu X Grivel MM Yu G Xu Y . Association between the duration of untreated psychosis and selective cognitive performance in community-dwelling individuals with chronic untreated schizophrenia in rural China. JAMA Psychiatry. (2020) 77:1116–26. doi: 10.1001/jamapsychiatry.2020.1619, PMID: 32639517 PMC7344798

[39] Chidarikire S Cross M Skinner I Cleary M . Treatments for people living with schizophrenia in Sub-Saharan Africa: an adapted realist review. Int Nurs Rev. (2018) 65:78–92. doi: 10.1111/inr.12391, PMID: 28543089

[40] Ayenew W Asmamaw G Bitew T . Antipsychotic polypharmacy among patients with schizophrenia in Africa: A systematic review and meta-analysis. Int J Neuropsychopharmacol. (2021) 24:956–64. doi: 10.1093/ijnp/pyab046, PMID: 34245271 PMC8653871

[41] Stevović LI Repišti S Radojičić T Sartorius N Tomori S Džubur Kulenović A . Non-pharmacological treatments for schizophrenia in Southeast Europe: An expert survey. Int J Soc Psychiatry. (2022) 68:1141–50. doi: 10.1177/00207640211023072, PMID: 34392727 PMC9310140

