# Supplementary material for: Global burden of schizophrenia in 204 countries and regions from 1990 to 2021 and machine learning-based projections to 2036: an analysis of the 2021 global burden of disease study
Source: Front Psychiatry. 2026 Jan 27;16:1684168. doi: 10.3389/fpsyt.2025.1684168 (PMC12887702; doi:10.3389/fpsyt.2025.1684168)
Supplement: Supplementary Figure 1 — Global distribution in ASPR and trends in EAPC of schizophrenia across 204 countries and territories for both sexes. (A) The ASPR per 100, 000 in 2021. (B) The EAPC of ASPR from 1990 to 2021. [file SupplementaryFile1.docx]

***Global burden, trends and forecast analysis of schizophrenia based on GBD 2021***

***Global burden, trends and forecast analysis of schizophrenia based on GBD 2021***

**Supplementary figures**

**Supplementary figure S1** Global distribution in ASPR and trends in EAPC of schizophrenia across 204 countries and territories for both sexes. (A) The ASPR per 100,000 in 2021. (B) The EAPC of ASPR from 1990 to 2021.

**Supplementary figure S2** Global distribution in ASDR and trends in EAPC of schizophrenia across 204 countries and territories for both sexes. (A) The ASDR per 100,000 in 2021. (B) The EAPC of ASDR from 1990 to 2021.

**Supplementary figure S3** Age, period and cohort effects on incidence of schizophrenia.

**Supplementary figure S4** Age, period and cohort effects on prevalence of schizophrenia.

**Supplementary figure S5** Age, period and cohort effects on DALYs of schizophrenia.

**Supplementary figure S6** The age, period, and birth cohort effects on the relative risks (RRs) for incidence, prevalence, and DALYs of schizophrenia. A, incidence. B, prevalence. C, DALYs.

**Supplementary figure S7** Decomposition analysis stratified by sex of incidence, prevalence, and DALYs of schizophrenia. A, incidence. B, prevalence. C, DALYs.

**Supplementary figure S8** Frontier analysis across 204 countries and territories based on the Socio-demographic Index (SDI) and the age-standardized disability-adjusted life years (DALYs) rate of schizophrenia in 2021.

**Supplementary figure S9** Health inequality analysis of incidence, prevalence, and DALY rates from 1990 to 2021. A, slope index analysis of inequality on DALY rates. B, concentration index analysis on DALY rates.

**Supplementary Tables**

**Supplementary Table S1** Performance evaluation results of the optimal statistical models and machine learning models selected through rolling cross-validation

**Supplementary Table S2** The cases of schizophrenia and ASIR of schizophrenia in 1990 and 2021, and its temporal trends from 1990-2021 among 204 countries and territories.

**Supplementary Table S3** The cases of prevalence and ASPR of schizophrenia in 1990 and 2021, and its temporal trends from 1990-2021 among 204 countries and territories.

**Supplementary Table S4** The cases of DALYs and ASDR of schizophrenia in 1990 and 2021, and its temporal trends from 1990-2021 among 204 countries and territories.

**Supplementary Table S5** Joinpoint regression analysis: trends in age-standardized incidence, prevalence, DALYs (per 100,000 persons) among both sexes, males, and females, 1990–2021


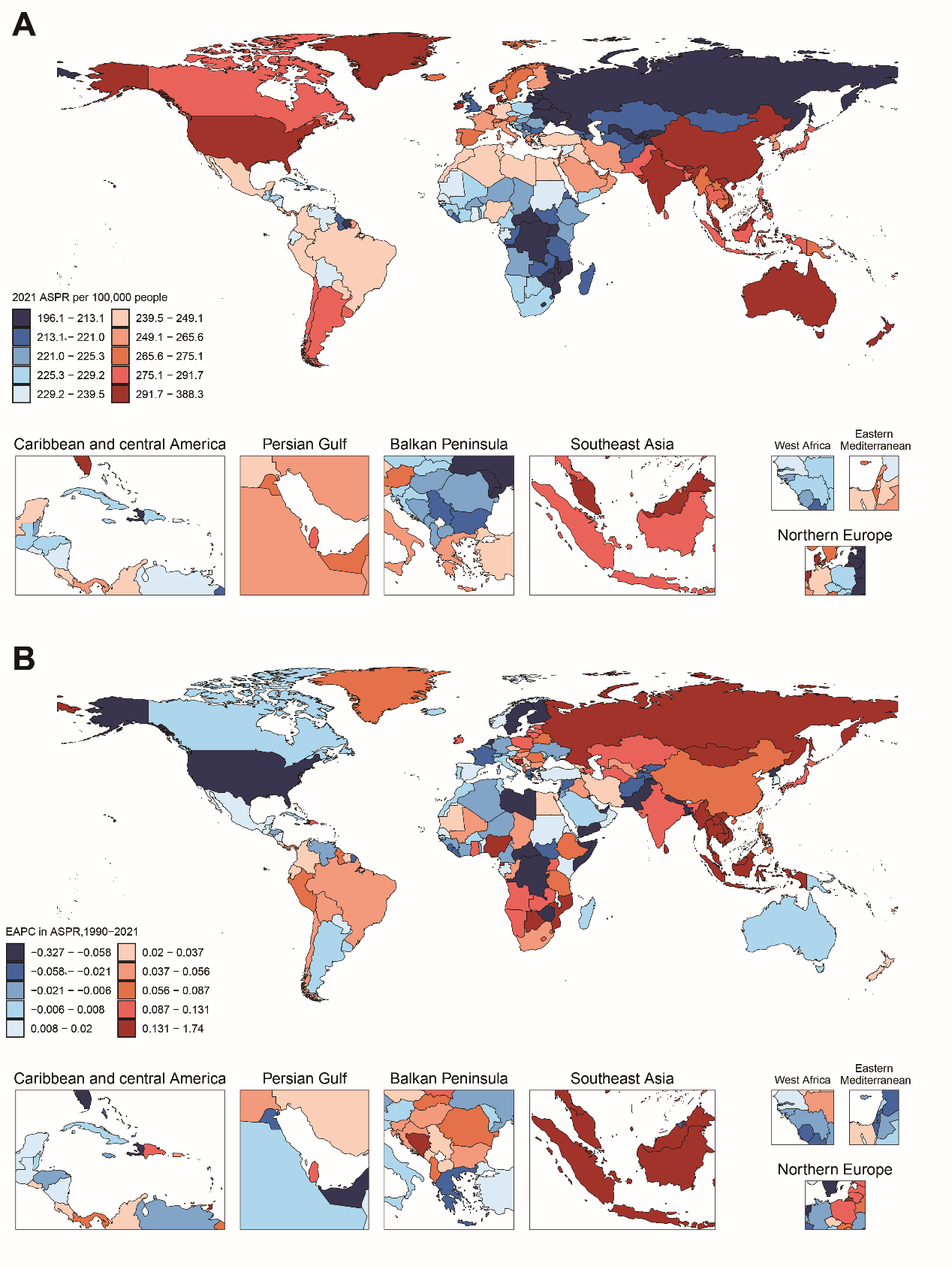


**Supplementary figure S1** Global distribution in ASPR and trends in EAPC of schizophrenia across 204 countries and territories for both sexes. (A) The ASPR per 100,000 in 2021. (B) The EAPC of ASPR from 1990 to 2021.


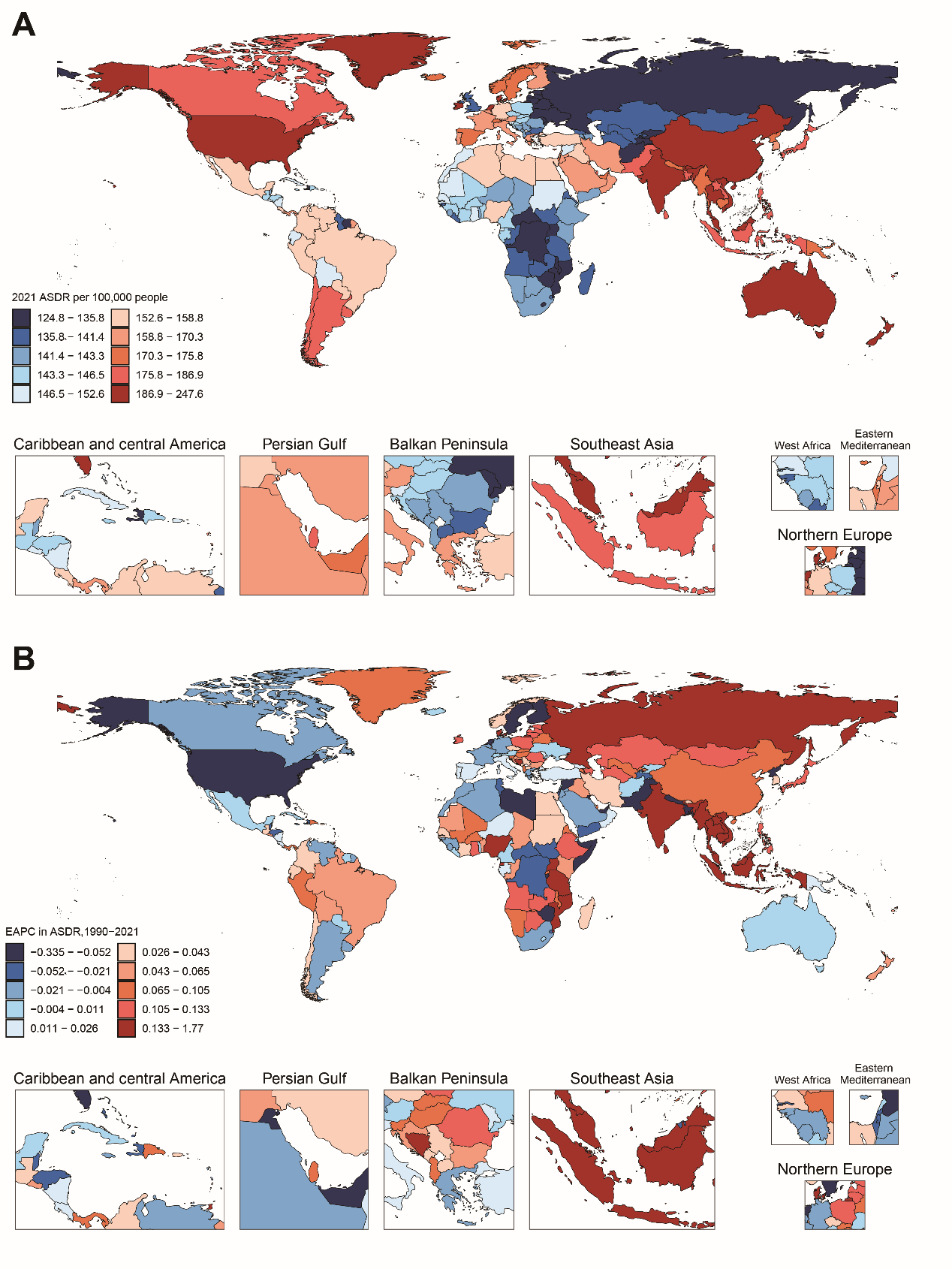


**Supplementary figure S2** Global distribution in ASDR and trends in EAPC of schizophrenia across 204 countries and territories for both sexes. (A) The ASDR per 100,000 in 2021. (B) The EAPC of ASDR from 1990 to 2021.


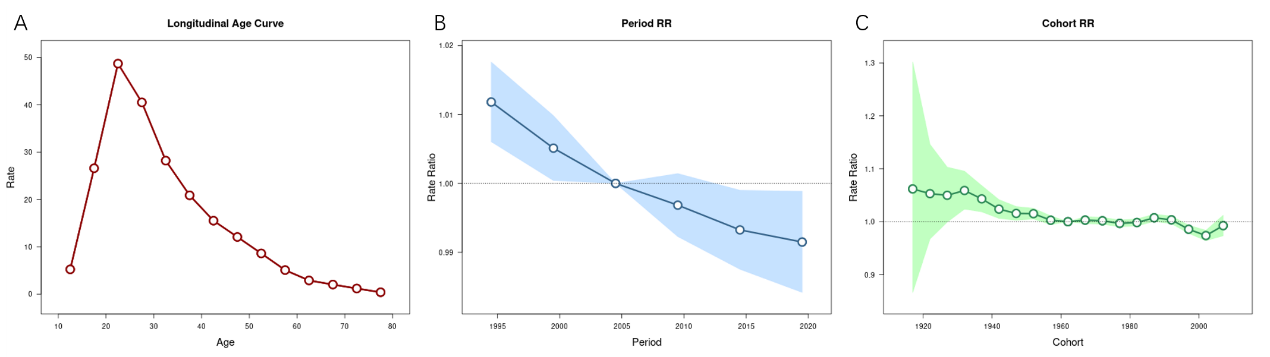


**Supplementary figure S3** Age, period and cohort effects on incidence of schizophrenia.


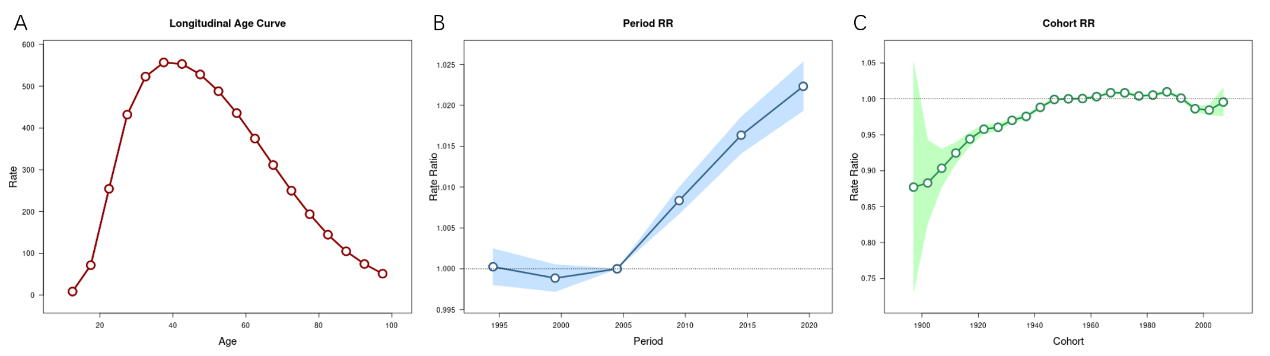


**Supplementary figure S4** Age, period and cohort effects on prevalence of schizophrenia.


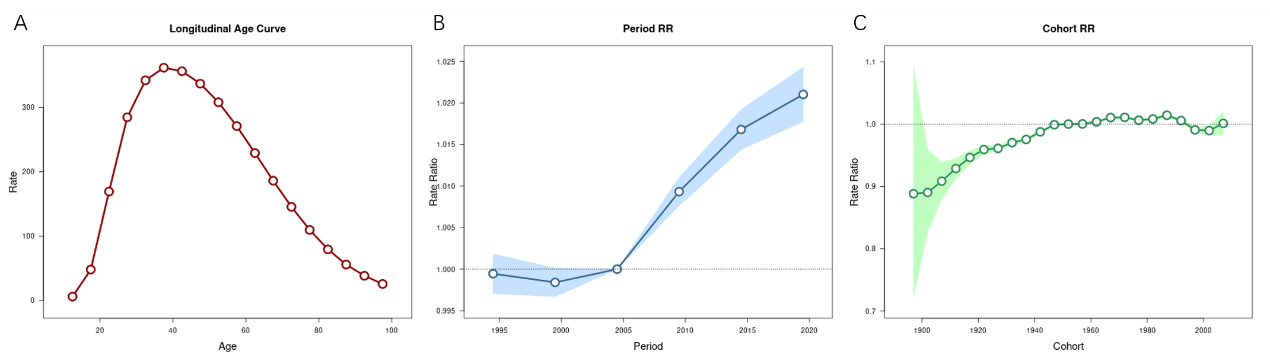


**Supplementary figure S5** Age, period and cohort effects on DALYs of schizophrenia.


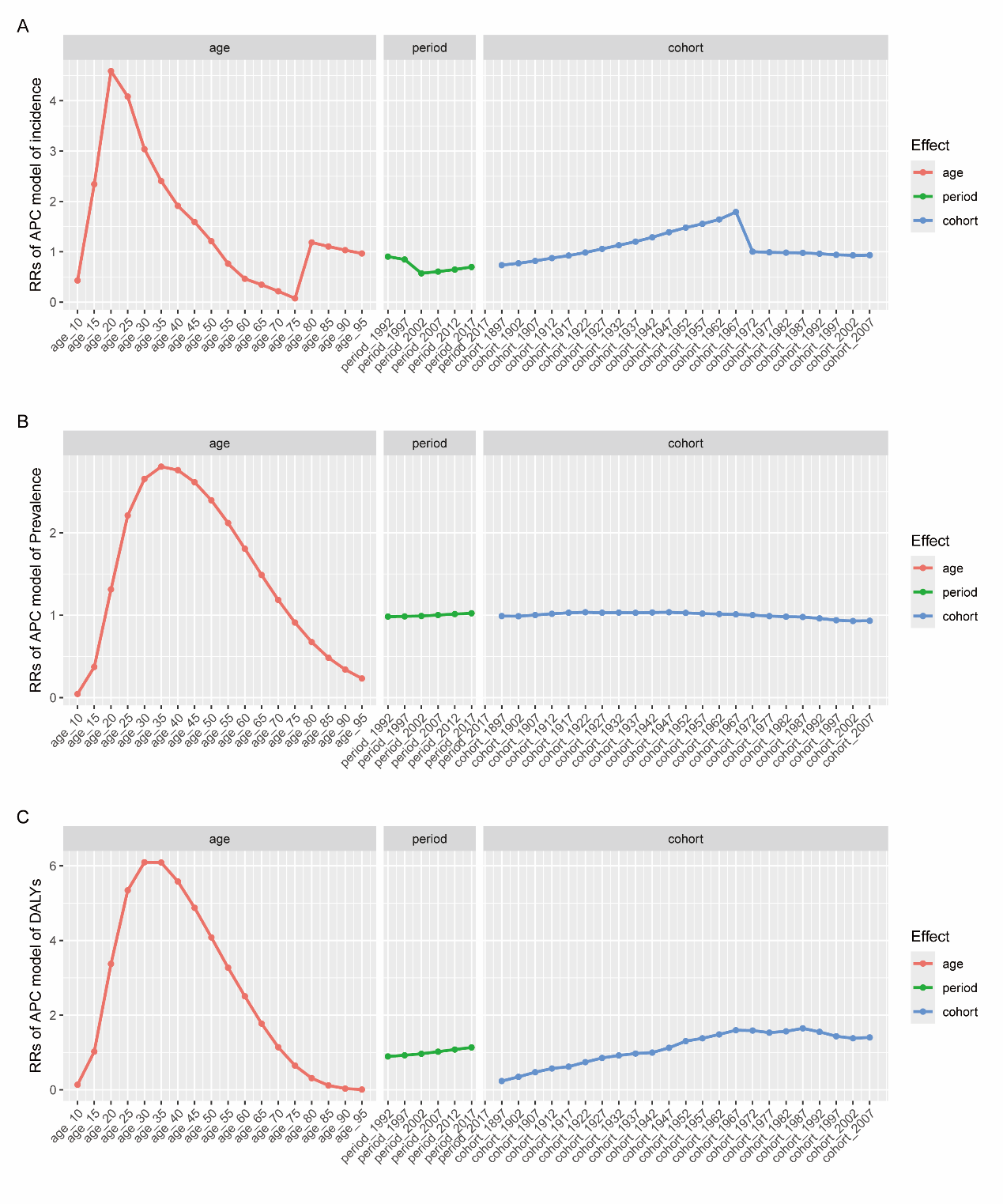


**Supplementary figure S6** The age, period, and birth cohort effects on the relative risks (RRs) for incidence, prevalence, and DALYs of schizophrenia. A, incidence. B, prevalence. C, DALYs.


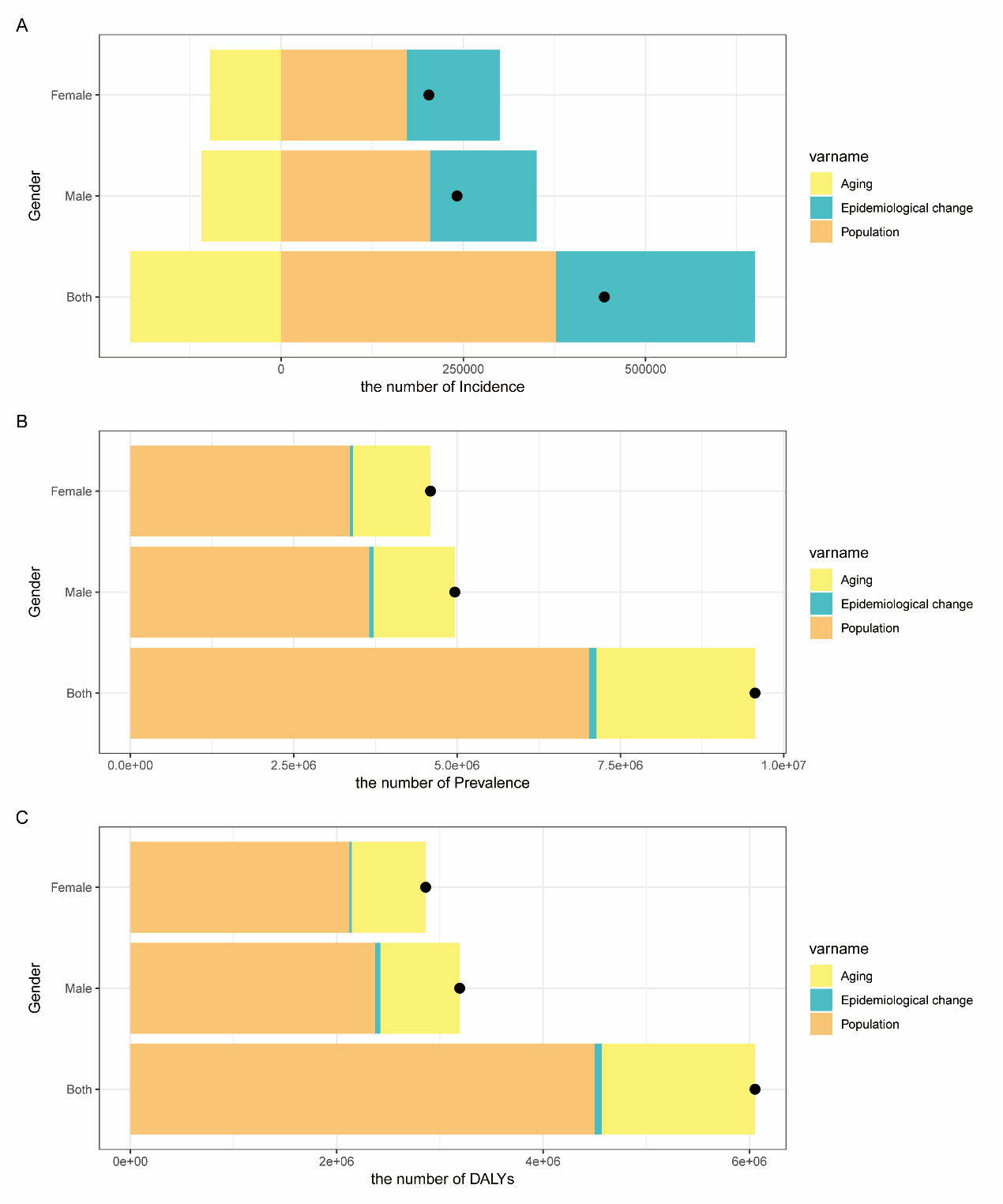


**Supplementary figure S7** Decomposition analysis stratified by sex of incidence, prevalence, and DALYs of schizophrenia. A, incidence. B, prevalence. C, DALYs.


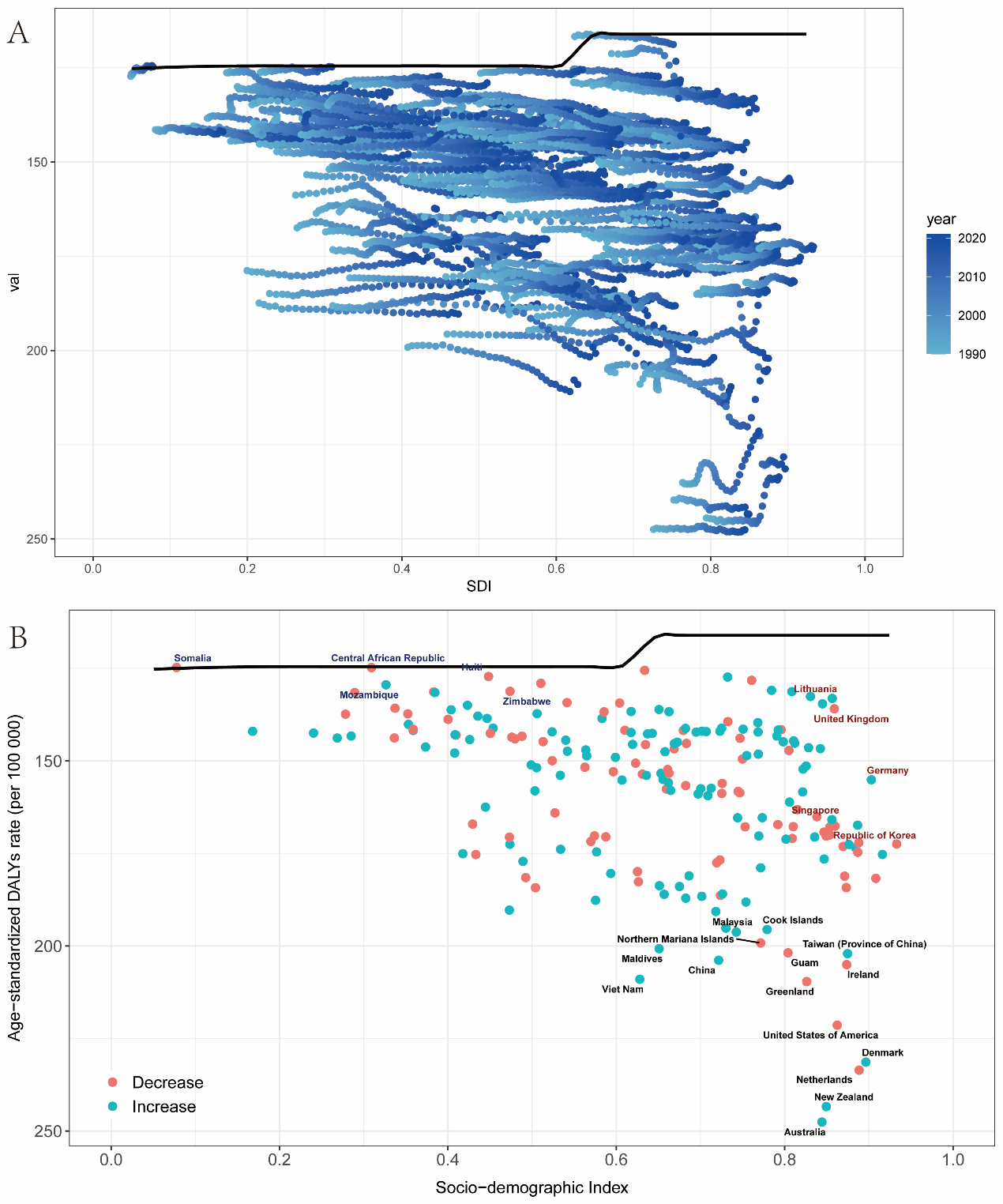


**Supplementary figure S8** Frontier analysis across 204 countries and territories based on the Socio-demographic Index (SDI) and the age-standardized disability-adjusted life years (DALYs) rate of schizophrenia in 2021.


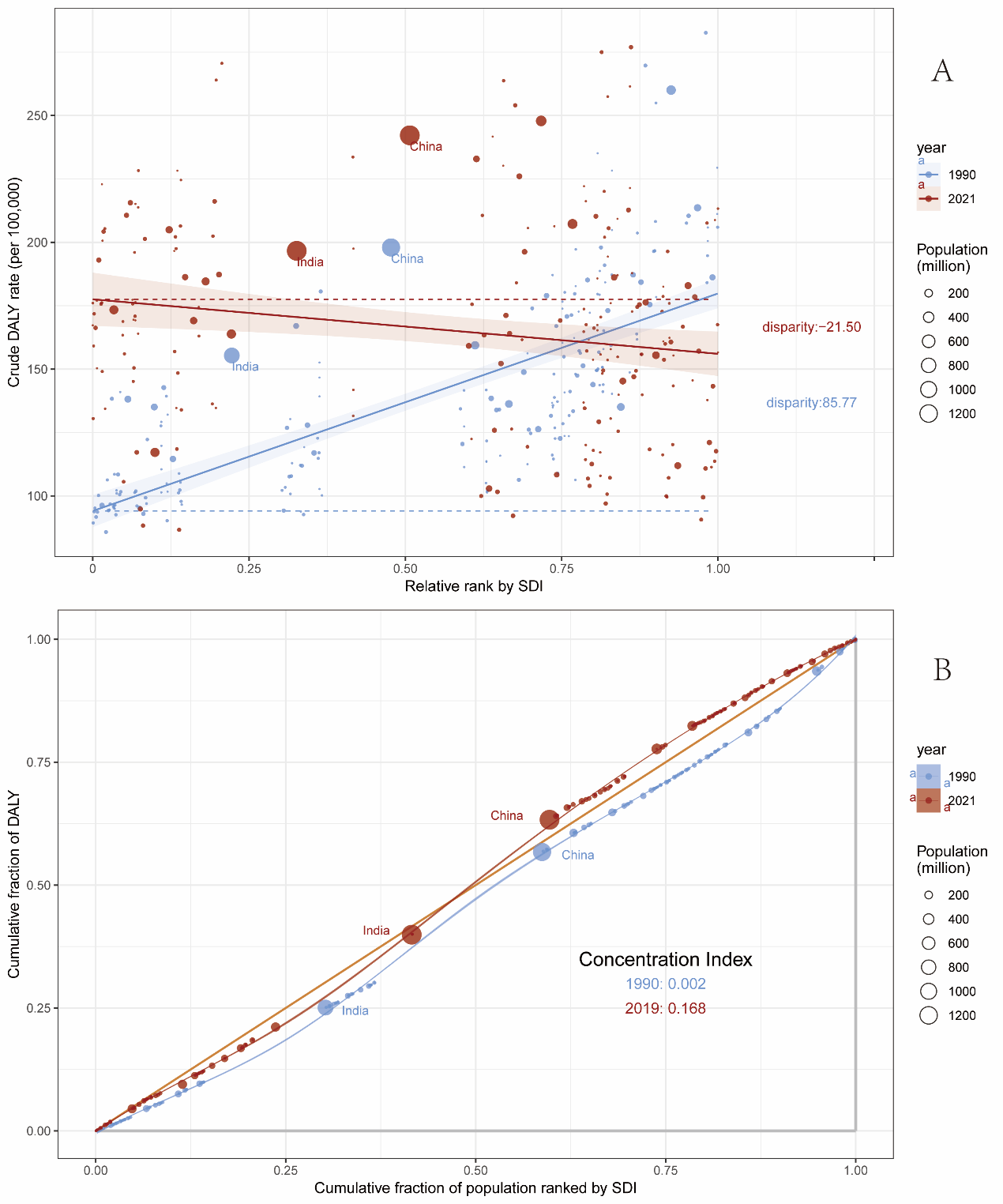


**Supplementary figure S9** Health inequality analysis of incidence, prevalence, and DALY rates from 1990 to 2021. A, slope index analysis of inequality on DALY rates. B, concentration index analysis on DALY rates.

**Supplementary Table S1** Performance evaluation results of the optimal statistical models and machine learning models selected through rolling cross-validation

|  | Sex | Model | Metric | | | | | |
| --- | --- | --- | --- | --- | --- | --- | --- | --- |
|  |  |  | MSE | RMSE | MAE | MAPE | MASE | R2 |
| ASIR | Both | ARIMA | 0.000276 | 0.01607906 | 0.013454 | 0.086924 | 1.473647 | -6.2303 |
| ASIR | Male | Elastic Net | 0.000609 | 0.023926 | 0.02163 | 0.131021 | 1.99359 | -1.55804 |
| ASIR | Female | ARIMA | 0.000173 | 0.012441 | 0.009806 | 0.068006 | 1.132426 | -5.17871 |
| ASPR | Both | Prophet | 0.558612 | 0.678884 | 0.568664 | 0.205604 | 5.080351 | -9.76185 |
| ASPR | Male | Elastic Net | 0.410589 | 0.491871 | 0.399083 | 0.137095 | 2.987977 | -1.09819 |
| ASPR | Female | Prophet | 0.304472 | 0.470266 | 0.429652 | 0.163881 | 3.529559 | -20.2801 |
| ASDR | Both | Elastic Net | 0.125929 | 0.325917 | 0.300519 | 0.1691 | 3.22974 | -4.36563 |
| ASDR | Male | Elastic Net | 0.134292 | 0.288644 | 0.239199 | 0.126577 | 2.306808 | -0.5627 |
| ASDR | Female | Elastic Net | 0.186282 | 0.425538 | 0.395794 | 0.237885 | 4.040088 | -62.6598 |

| **Supplementary Table S2** The cases of schizophrenia and ASIR of schizophrenia in 1990 and 2021, and its temporal trends from 1990-2021 among 204 countries and territories. |
| --- |

| Characteristics | 1990 | | 2021 | | 1990-2021  EAPC (95%CI) |
| --- | --- | --- | --- | --- | --- |
|  | **Cases of incidence (95%UI)** | **ASIR per 100,000 (95%UI)** | **Cases of incidence (95%UI)** | **ASIR per 100,000 (95%UI)** |  |
| Afghanistan | 1015 (766 to 1328) | 12.54 (9.67 to 16.13) | 1411 (1090 to 1808) | 12.6 (9.64 to 15.97) | -0.0443 (-0.0581 to -0.0306) |
| Albania | 932 (703 to 1204) | 14.14 (11 to 17.93) | 1959 (1499 to 2494) | 14.02 (11.05 to 17.75) | 0.0072 (0.0019 to 0.0125) |
| Algeria | 759 (579 to 961) | 14.66 (11.45 to 18.39) | 1840 (1387 to 2317) | 14.53 (11.38 to 18.09) | -0.0466 (-0.0504 to -0.0429) |
| American Samoa | 59 (46 to 75) | 14.58 (11.26 to 18.44) | 92 (73 to 115) | 14.62 (11.26 to 18.49) | -0.0296 (-0.0417 to -0.0175) |
| Andorra | 3321 (2498 to 4326) | 14.08 (10.96 to 17.81) | 8118 (6229 to 10305) | 14.01 (11 to 17.66) | -0.0155 (-0.0227 to -0.0084) |
| Angola | 627 (467 to 819) | 14.57 (11.25 to 18.6) | 1120 (861 to 1416) | 14.19 (10.83 to 18.02) | 0.006 (-0.0006 to 0.0127) |
| Antigua and Barbuda | 517 (403 to 659) | 11.55 (8.95 to 14.8) | 392 (308 to 496) | 11.54 (8.85 to 14.69) | 0.004 (-0.0101 to 0.0181) |
| Argentina | 14 (10 to 19) | 12.5 (9.5 to 15.8) | 14 (11 to 18) | 12.43 (9.7 to 15.83) | -0.0164 (-0.0237 to -0.0091) |
| Armenia | 261 (197 to 337) | 12.44 (9.43 to 16.06) | 266 (207 to 336) | 12.47 (9.61 to 15.94) | -0.0071 (-0.0152 to 0.001) |
| Australia | 706 (542 to 901) | 12.57 (9.68 to 16.08) | 393 (307 to 494) | 12.53 (9.5 to 15.9) | 0.0168 (0.01 to 0.0235) |
| Austria | 14 (10 to 18) | 17.45 (13.42 to 22.01) | 19 (14 to 23) | 17.68 (13.64 to 22.24) | 0.0024 (-0.0026 to 0.0075) |
| Azerbaijan | 11282 (9188 to 13572) | 17.12 (14.35 to 20.29) | 21175 (17465 to 25293) | 17.14 (14.31 to 20.45) | 0.0244 (0.0124 to 0.0364) |
| Bahamas | 579 (471 to 701) | 15.96 (13 to 19.45) | 705 (552 to 863) | 16.08 (12.39 to 20.2) | -0.0288 (-0.0323 to -0.0253) |
| Bahrain | 10 (8 to 14) | 12.51 (9.83 to 15.94) | 14 (11 to 18) | 12.59 (9.74 to 15.93) | -0.0016 (-0.0061 to 0.003) |
| Bangladesh | 304 (228 to 394) | 14.68 (11.27 to 18.68) | 832 (635 to 1086) | 14.61 (11.2 to 18.69) | -0.1113 (-0.1157 to -0.1069) |
| Barbados | 1449 (1144 to 1811) | 14.21 (11.19 to 17.91) | 1295 (1022 to 1611) | 14.28 (11.1 to 18.23) | -0.0631 (-0.0971 to -0.0291) |
| Belarus | 662 (506 to 848) | 12.46 (9.5 to 15.99) | 610 (477 to 767) | 12.6 (9.62 to 16.1) | 0.0125 (0.0036 to 0.0214) |
| Belgium | 3275 (2509 to 4243) | 17.41 (13.6 to 22.19) | 6337 (4890 to 8006) | 17.69 (13.67 to 22.36) | -0.0056 (-0.0131 to 0.0019) |
| Belize | 735 (603 to 886) | 20.38 (16.74 to 24.57) | 991 (814 to 1196) | 20.58 (16.89 to 24.88) | -0.0404 (-0.0451 to -0.0358) |
| Benin | 54 (43 to 68) | 14.46 (11.15 to 18.22) | 56 (44 to 71) | 14.54 (11.22 to 18.64) | -0.0373 (-0.0468 to -0.0278) |
| Bermuda | 487 (364 to 623) | 14.58 (11.36 to 18.42) | 1219 (935 to 1538) | 14.47 (11.33 to 18.26) | 0.0105 (0.0002 to 0.0209) |
| Bhutan | 448 (339 to 577) | 12.4 (9.67 to 15.79) | 326 (250 to 417) | 12.49 (9.49 to 15.98) | -0.0629 (-0.0661 to -0.0597) |
| Bolivia (Plurinational State of) | 1182 (911 to 1501) | 14.45 (11.13 to 18.24) | 1185 (940 to 1496) | 14.46 (11.24 to 18.31) | -0.0046 (-0.0125 to 0.0033) |
| Bosnia and Herzegovina | 1746 (1281 to 2264) | 14.19 (10.99 to 18.01) | 1923 (1470 to 2522) | 13.9 (10.61 to 17.51) | 0.0434 (0.0343 to 0.0525) |
| Botswana | 82 (63 to 105) | 12.44 (9.57 to 15.85) | 71 (55 to 90) | 12.45 (9.47 to 15.92) | 0.0315 (0.0232 to 0.0397) |
| Brazil | 462 (355 to 599) | 12.59 (9.74 to 16.13) | 359 (278 to 455) | 12.59 (9.72 to 16.07) | 0.0005 (-0.0059 to 0.0069) |
| Brunei Darussalam | 2070 (1550 to 2660) | 14.6 (11.31 to 18.45) | 5230 (3990 to 6682) | 14.56 (11.3 to 18.25) | -0.0222 (-0.0271 to -0.0173) |
| Bulgaria | 0 (0 to 0) | 17.03 (13.44 to 21.71) | 0 (0 to 0) | 17.25 (13.45 to 21.85) | 0.0065 (0.0032 to 0.0099) |
| Burkina Faso | 4024 (3145 to 5051) | 17.42 (13.78 to 21.57) | 4078 (3224 to 4989) | 18.36 (14.34 to 23.01) | -0.0401 (-0.0473 to -0.0328) |
| Burundi | 3233 (2476 to 4114) | 17.12 (13.39 to 21.48) | 3798 (3002 to 4755) | 17.27 (13.49 to 21.85) | -0.014 (-0.0215 to -0.0066) |
| Cabo Verde | 9 (7 to 12) | 12.62 (9.81 to 15.99) | 9 (7 to 11) | 12.52 (9.68 to 15.88) | -0.0305 (-0.0367 to -0.0243) |
| Cambodia | 651 (546 to 782) | 15.09 (12.63 to 18.19) | 760 (635 to 919) | 15.09 (12.51 to 18.2) | 0.089 (0.0639 to 0.114) |
| Cameroon | 123 (113 to 137) | 9.72 (8.92 to 10.77) | 168 (134 to 210) | 12.59 (9.82 to 15.79) | -0.0407 (-0.0462 to -0.0352) |
| Canada | 7995 (5938 to 10208) | 14.15 (10.88 to 17.98) | 15385 (11810 to 19623) | 14.07 (10.89 to 17.89) | 0.1544 (0.1126 to 0.1963) |
| Central African Republic | 4785 (3602 to 6242) | 13.41 (10.27 to 17.19) | 7154 (5457 to 9256) | 13.43 (10.23 to 17.28) | -0.0149 (-0.0191 to -0.0106) |
| Chad | 1139 (855 to 1478) | 13.59 (10.54 to 17.07) | 4251 (3136 to 5559) | 13.48 (10.46 to 17.09) | -0.0151 (-0.0232 to -0.007) |
| Chile | 10 (7 to 12) | 14.75 (11.46 to 18.76) | 12 (9 to 15) | 14.66 (11.32 to 18.62) | 0.0019 (-0.0069 to 0.0107) |
| China | 317 (237 to 411) | 14.97 (11.36 to 19.18) | 801 (625 to 1025) | 14.79 (11.38 to 18.86) | -0.0398 (-0.0691 to -0.0105) |
| Colombia | 13 (10 to 17) | 16.65 (13.09 to 20.86) | 21 (16 to 27) | 16.58 (12.98 to 20.85) | -0.0029 (-0.0112 to 0.0054) |
| Comoros | 4902 (3699 to 6192) | 15.21 (11.55 to 19.25) | 7154 (5444 to 9081) | 15.23 (11.51 to 19.33) | -0.004 (-0.0102 to 0.0022) |
| Congo | 542 (411 to 706) | 13.99 (10.81 to 17.78) | 774 (600 to 987) | 14.07 (10.9 to 17.78) | 0.0018 (-0.0063 to 0.0099) |
| Cook Islands | 752 (569 to 961) | 14.59 (11.4 to 18.46) | 2226 (1671 to 2866) | 14.52 (11.45 to 18.27) | 0.0175 (0.0079 to 0.0272) |
| Costa Rica | 16 (12 to 21) | 17.2 (13.47 to 21.76) | 18 (13 to 22) | 17.27 (13.33 to 21.77) | -0.008 (-0.0139 to -0.0021) |
| Croatia | 9 (7 to 12) | 17.77 (13.72 to 22.28) | 8 (7 to 10) | 17.53 (13.72 to 22.16) | 0.0119 (0.0064 to 0.0173) |
| Cuba | 94 (71 to 122) | 14.8 (11.37 to 18.81) | 274 (208 to 348) | 14.79 (11.15 to 18.7) | -0.0173 (-0.0289 to -0.0058) |
| Cyprus | 136 (101 to 174) | 14.61 (11.47 to 18.53) | 354 (267 to 452) | 14.5 (11.27 to 18.24) | -0.0035 (-0.007 to 0.0001) |
| Czechia | 1092 (838 to 1386) | 14.62 (11.48 to 18.57) | 3170 (2366 to 4077) | 14.53 (11.29 to 18.39) | -0.0023 (-0.0096 to 0.005) |
| C么te d'Ivoire | 3070 (2472 to 3724) | 14.36 (11.99 to 17.25) | 7636 (6215 to 9182) | 14.27 (11.89 to 17.05) | -0.0169 (-0.0207 to -0.0131) |
| Democratic People's Republic of Korea | 1688 (1273 to 2189) | 14.47 (11.32 to 18.46) | 4024 (3053 to 5146) | 14.45 (11.15 to 18.22) | -0.0819 (-0.098 to -0.0658) |
| Democratic Republic of the Congo | 313 (236 to 406) | 12.58 (9.72 to 15.99) | 391 (301 to 500) | 12.52 (9.69 to 15.79) | -0.033 (-0.0398 to -0.0262) |
| Denmark | 1159 (883 to 1478) | 14.61 (11.48 to 18.25) | 3063 (2317 to 3951) | 14.49 (11.24 to 18.18) | 1.8456 (1.5062 to 2.1861) |
| Djibouti | 2698 (2012 to 3529) | 13.88 (10.75 to 17.76) | 6501 (4854 to 8410) | 13.78 (10.59 to 17.37) | -0.0079 (-0.0151 to -0.0006) |
| Dominica | 179 (140 to 226) | 11.72 (9.03 to 14.75) | 130 (103 to 165) | 11.85 (9.08 to 15.09) | -0.0354 (-0.0438 to -0.027) |
| Dominican Republic | 578 (438 to 739) | 14.64 (11.51 to 18.35) | 1323 (995 to 1698) | 14.46 (11.23 to 18.34) | 0.0394 (0.0347 to 0.0441) |
| Ecuador | 659 (484 to 845) | 12.41 (9.51 to 15.79) | 1317 (991 to 1689) | 12.29 (9.4 to 15.64) | 0.0003 (-0.0075 to 0.0081) |
| Egypt | 4 (3 to 5) | 14.65 (11.51 to 18.42) | 4 (3 to 5) | 14.55 (11.25 to 18.57) | -0.0311 (-0.0398 to -0.0224) |
| El Salvador | 983 (754 to 1251) | 14.06 (10.98 to 17.69) | 2775 (2106 to 3538) | 13.96 (11.03 to 17.6) | -0.0104 (-0.0158 to -0.0049) |
| Equatorial Guinea | 1621 (1204 to 2074) | 13.89 (10.68 to 17.78) | 4714 (3575 to 6005) | 13.61 (10.51 to 17.27) | 0.0786 (0.0599 to 0.0973) |
| Eritrea | 709 (534 to 910) | 16.95 (13.34 to 21.33) | 1869 (1444 to 2417) | 17.05 (13.4 to 21.71) | -0.0058 (-0.0123 to 0.0006) |
| Estonia | 23 (17 to 30) | 12.47 (9.65 to 15.82) | 60 (46 to 76) | 12.37 (9.62 to 15.59) | 0.0361 (0.0295 to 0.0427) |
| Eswatini | 47067 (38456 to 57258) | 17.93 (14.64 to 21.63) | 50622 (41824 to 60838) | 17.05 (13.9 to 20.52) | -0.0004 (-0.0074 to 0.0066) |
| Ethiopia | 44 (34 to 58) | 14.56 (11.24 to 18.67) | 77 (60 to 99) | 14.42 (11.17 to 18.25) | -0.0454 (-0.0534 to -0.0374) |
| Fiji | 25 (18 to 32) | 16.81 (12.99 to 21.2) | 54 (41 to 69) | 16.82 (13.06 to 21.36) | 0.0248 (0.0123 to 0.0372) |
| Finland | 728 (558 to 916) | 14.84 (11.51 to 18.71) | 1308 (1022 to 1640) | 14.53 (11.31 to 18.35) | -0.1911 (-0.2382 to -0.144) |
| France | 5572 (5038 to 6166) | 14.02 (12.66 to 15.5) | 5624 (4892 to 6345) | 14.37 (12.9 to 16) | -0.0193 (-0.0264 to -0.0122) |
| Gabon | 1213 (938 to 1561) | 11.64 (8.99 to 14.93) | 951 (739 to 1193) | 11.7 (8.78 to 14.84) | 0.0028 (-0.0007 to 0.0063) |
| Gambia | 650 (488 to 826) | 16.56 (12.72 to 20.91) | 1370 (1050 to 1736) | 16.77 (12.99 to 21.09) | -0.0208 (-0.0294 to -0.0123) |
| Georgia | 476 (368 to 603) | 12.77 (9.9 to 16.18) | 375 (293 to 469) | 12.79 (9.86 to 16.32) | -0.0087 (-0.0179 to 0.0005) |
| Germany | 461 (355 to 590) | 15.25 (11.7 to 19.6) | 499 (387 to 632) | 15.24 (11.74 to 19.38) | -0.0133 (-0.0274 to 0.0009) |
| Ghana | 8 (6 to 11) | 12.77 (9.76 to 16.16) | 12 (9 to 15) | 12.73 (9.74 to 16.22) | -0.002 (-0.0074 to 0.0034) |
| Greece | 52 (40 to 67) | 13.89 (10.88 to 17.65) | 239 (179 to 308) | 14.03 (10.89 to 17.78) | -0.0189 (-0.0234 to -0.0145) |
| Greenland | 104 (78 to 134) | 12.26 (9.6 to 15.52) | 101 (78 to 129) | 12.25 (9.54 to 15.28) | 0.0466 (0.01 to 0.0832) |
| Grenada | 3 (2 to 4) | 17.41 (13.62 to 22.02) | 3 (2 to 4) | 17.69 (13.93 to 22.32) | 0.018 (0.0148 to 0.0213) |
| Guam | 612 (475 to 777) | 12.53 (9.64 to 15.98) | 450 (351 to 565) | 12.61 (9.7 to 16.23) | -0.022 (-0.0337 to -0.0102) |
| Guatemala | 87 (66 to 114) | 15.22 (11.56 to 19.51) | 615 (459 to 809) | 15.3 (11.78 to 19.7) | -0.0232 (-0.0321 to -0.0144) |
| Guinea | 125 (95 to 158) | 15.64 (12.17 to 19.56) | 230 (171 to 297) | 15.6 (12.17 to 19.42) | -0.019 (-0.0261 to -0.0119) |
| Guinea-Bissau | 105 (78 to 135) | 15.11 (11.49 to 19.24) | 133 (100 to 171) | 14.85 (11.29 to 18.89) | -0.022 (-0.0302 to -0.0138) |
| Guyana | 1568 (1190 to 2009) | 14.16 (11.1 to 17.75) | 4091 (3101 to 5275) | 14.09 (10.89 to 17.85) | -0.0067 (-0.0118 to -0.0016) |
| Haiti | 436 (336 to 560) | 11.66 (8.96 to 14.92) | 273 (217 to 346) | 11.83 (9.09 to 15.18) | -0.0443 (-0.0518 to -0.0368) |
| Honduras | 8042 (6485 to 9688) | 14.43 (11.93 to 17.38) | 12729 (10397 to 15432) | 14.35 (11.87 to 17.24) | -0.0434 (-0.0498 to -0.0371) |
| Hungary | 1274 (943 to 1651) | 14.35 (10.84 to 18.28) | 1652 (1281 to 2089) | 14.22 (10.78 to 18.06) | 0.0205 (0.0129 to 0.0281) |
| Iceland | 696 (526 to 896) | 13.08 (10.13 to 16.43) | 894 (676 to 1145) | 13.04 (10.09 to 16.58) | -0.0018 (-0.0071 to 0.0035) |
| India | 574 (419 to 756) | 14.44 (11 to 18.61) | 2004 (1496 to 2550) | 14.3 (10.9 to 18.08) | 0.0472 (0.0169 to 0.0775) |
| Indonesia | 39 (30 to 49) | 14.56 (11.22 to 18.19) | 49 (38 to 61) | 14.56 (11.38 to 18.47) | 0.0697 (0.0413 to 0.0981) |
| Iran (Islamic Republic of) | 48 (35 to 62) | 14.83 (11.39 to 18.74) | 94 (72 to 120) | 14.75 (11.39 to 18.59) | -0.0275 (-0.0337 to -0.0214) |
| Iraq | 282 (206 to 370) | 14.31 (10.82 to 18.29) | 801 (594 to 1030) | 14.15 (10.86 to 17.87) | -0.027 (-0.0376 to -0.0165) |
| Ireland | 536 (405 to 681) | 13.45 (10.35 to 17.08) | 1064 (804 to 1367) | 13.48 (10.27 to 17.19) | 0.1597 (0.0831 to 0.2364) |
| Israel | 184 (141 to 232) | 13.57 (10.7 to 17.1) | 279 (214 to 357) | 13.53 (10.59 to 17.11) | -0.0463 (-0.0611 to -0.0315) |
| Italy | 4 (3 to 5) | 17.78 (13.68 to 22.42) | 3 (2 to 4) | 17.88 (13.94 to 22.6) | 0.0087 (0.0073 to 0.0102) |
| Jamaica | 251820 (215196 to 291261) | 18.19 (15.71 to 20.97) | 236175 (201684 to 275708) | 18.36 (15.86 to 21.18) | -0.0211 (-0.0257 to -0.0165) |
| Japan | 134 (101 to 174) | 14.06 (10.81 to 17.9) | 270 (208 to 346) | 14.08 (10.93 to 17.77) | 0.2235 (0.1092 to 0.3378) |
| Jordan | 712 (542 to 924) | 14.07 (10.9 to 17.97) | 1781 (1366 to 2281) | 13.98 (10.97 to 17.77) | -0.0476 (-0.0588 to -0.0363) |
| Kazakhstan | 9 (7 to 11) | 13.04 (9.91 to 16.59) | 7 (5 to 8) | 13.04 (10.04 to 16.67) | 0.0322 (0.0254 to 0.0389) |
| Kenya | 102 (77 to 132) | 13.79 (10.76 to 17.29) | 174 (131 to 222) | 13.72 (10.59 to 17.25) | -0.0159 (-0.0216 to -0.0103) |
| Kiribati | 2 (1 to 2) | 16.66 (13 to 21.15) | 2 (2 to 3) | 16.95 (13.1 to 21.22) | -0.0079 (-0.0195 to 0.0036) |
| Kuwait | 273 (204 to 350) | 12.27 (9.45 to 15.48) | 412 (325 to 525) | 12.35 (9.67 to 15.67) | -0.0605 (-0.0754 to -0.0456) |
| Kyrgyzstan | 61 (46 to 79) | 14.18 (11.12 to 17.8) | 195 (151 to 247) | 14.1 (11.02 to 17.73) | -0.0318 (-0.0374 to -0.0262) |
| Lao People's Democratic Republic | 1068 (826 to 1362) | 14.72 (11.36 to 18.6) | 1176 (936 to 1476) | 14.63 (11.43 to 18.64) | 0.0955 (0.07 to 0.121) |
| Latvia | 3024 (2272 to 3917) | 13.38 (10.28 to 17.04) | 5262 (4032 to 6794) | 13.45 (10.34 to 17.21) | 0.03 (0.0245 to 0.0356) |
| Lebanon | 2481 (1822 to 3178) | 14.62 (11.18 to 18.51) | 6850 (5283 to 8654) | 14.54 (11.19 to 18.29) | -0.0284 (-0.0371 to -0.0197) |
| Lesotho | 61 (46 to 78) | 14.26 (11.06 to 17.97) | 113 (87 to 145) | 14.18 (11.04 to 18.06) | 0.0048 (-0.0029 to 0.0125) |
| Liberia | 7063 (5939 to 8474) | 12.38 (10.41 to 14.71) | 6888 (5835 to 8316) | 11.23 (9.45 to 13.45) | -0.0496 (-0.0558 to -0.0435) |
| Libya | 1002 (750 to 1279) | 14.69 (11.41 to 18.59) | 2333 (1770 to 2977) | 14.58 (11.47 to 18.38) | -0.1057 (-0.1161 to -0.0953) |
| Lithuania | 5998 (4872 to 7292) | 11.8 (9.72 to 14.33) | 4479 (3699 to 5493) | 11.71 (9.62 to 14.2) | 0.0265 (0.0178 to 0.0352) |
| Luxembourg | 447 (337 to 570) | 14.1 (10.99 to 17.69) | 976 (742 to 1242) | 13.99 (10.99 to 17.62) | 0.0228 (0.0172 to 0.0283) |
| Madagascar | 437 (333 to 555) | 14.4 (11.13 to 18.04) | 853 (663 to 1094) | 14.34 (11.03 to 18.26) | -0.0188 (-0.0226 to -0.015) |
| Malawi | 30 (22 to 38) | 18.37 (14.21 to 23.18) | 28 (22 to 35) | 18.18 (14.14 to 22.8) | -0.0172 (-0.0252 to -0.0093) |
| Malaysia | 192 (144 to 249) | 13.87 (10.88 to 17.64) | 367 (279 to 472) | 13.87 (10.71 to 17.59) | 0.1023 (0.0764 to 0.1282) |
| Maldives | 329 (246 to 424) | 14.59 (11.26 to 18.51) | 807 (619 to 1024) | 14.46 (11.2 to 18.39) | 0.1828 (0.144 to 0.2217) |
| Mali | 45 (38 to 52) | 10.8 (9.3 to 12.5) | 63 (55 to 72) | 10.77 (9.37 to 12.46) | -0.0272 (-0.0339 to -0.0205) |
| Malta | 1381 (1046 to 1789) | 14.61 (11.33 to 18.6) | 4583 (3480 to 5893) | 14.47 (11.34 to 18.23) | 0.0104 (0.0031 to 0.0177) |
| Marshall Islands | 218 (167 to 279) | 17.34 (13.46 to 21.87) | 222 (175 to 277) | 17.46 (13.73 to 22.02) | 0.0004 (-0.0089 to 0.0097) |
| Mauritania | 29 (22 to 38) | 17.19 (13.44 to 21.69) | 36 (28 to 46) | 17.22 (13.44 to 21.75) | -0.0191 (-0.0264 to -0.0119) |
| Mauritius | 1200 (929 to 1520) | 12.47 (9.5 to 15.86) | 1023 (797 to 1289) | 12.44 (9.52 to 15.94) | 0.0746 (0.0508 to 0.0983) |
| Mexico | 33903 (27688 to 40733) | 17.03 (14.3 to 20.37) | 51986 (43248 to 62004) | 17.14 (14.32 to 20.43) | -0.0068 (-0.0141 to 0.0005) |
| Micronesia (Federated States of) | 827 (624 to 1056) | 13.21 (10.21 to 16.87) | 1685 (1293 to 2146) | 13.19 (10.22 to 16.67) | 0.0135 (0.0023 to 0.0247) |
| Monaco | 8098 (6775 to 9704) | 13.86 (11.61 to 16.61) | 6834 (5726 to 8204) | 13.9 (11.59 to 16.69) | -0.0027 (-0.0152 to 0.0098) |
| Mongolia | 1642 (1272 to 2055) | 14.02 (11.09 to 17.65) | 4089 (3073 to 5274) | 13.92 (10.94 to 17.65) | 0.0206 (0.0132 to 0.0281) |
| Montenegro | 443 (336 to 568) | 13.56 (10.47 to 17.22) | 682 (527 to 871) | 13.55 (10.46 to 17.39) | -0.0001 (-0.004 to 0.0038) |
| Morocco | 1004 (761 to 1277) | 14.58 (11.51 to 18.42) | 3121 (2309 to 3985) | 14.48 (11.35 to 18.24) | -0.0467 (-0.0576 to -0.0358) |
| Mozambique | 3755 (2923 to 4758) | 16.96 (13.34 to 21.12) | 4633 (3693 to 5731) | 16.67 (13.21 to 20.83) | -0.013 (-0.0175 to -0.0085) |
| Myanmar | 18 (13 to 24) | 12.52 (9.6 to 16.03) | 23 (18 to 29) | 12.57 (9.62 to 15.92) | 0.0992 (0.0709 to 0.1275) |
| Namibia | 1266 (1071 to 1514) | 15.03 (12.87 to 17.77) | 1440 (1209 to 1759) | 15.23 (12.93 to 18.46) | 0.0144 (0.008 to 0.0208) |
| Nauru | 0 (0 to 0) | 17.36 (13.46 to 21.63) | 0 (0 to 0) | 17.45 (13.66 to 22.08) | -0.0582 (-0.1022 to -0.0142) |
| Nepal | 11 (8 to 14) | 18.43 (14.27 to 23.62) | 8 (7 to 10) | 18.07 (14.04 to 22.72) | -0.1332 (-0.145 to -0.1213) |
| Netherlands | 1345 (1020 to 1713) | 13.91 (10.76 to 17.69) | 4222 (3195 to 5453) | 13.91 (10.91 to 17.67) | -0.1339 (-0.168 to -0.0997) |
| New Zealand | 4715 (3889 to 5687) | 12.6 (10.41 to 15.23) | 4288 (3538 to 5202) | 12.74 (10.48 to 15.39) | 0.024 (0.0138 to 0.0342) |
| Nicaragua | 17 (13 to 21) | 16.8 (13.1 to 21.16) | 19 (14 to 24) | 16.87 (13.05 to 21.28) | -0.0142 (-0.0239 to -0.0046) |
| Niger | 987 (770 to 1247) | 12.04 (9.29 to 15.36) | 664 (524 to 840) | 12.07 (9.21 to 15.43) | -0.0378 (-0.0462 to -0.0294) |
| Nigeria | 118 (91 to 149) | 14.47 (11.26 to 18.13) | 200 (156 to 253) | 14.48 (11.18 to 18.24) | 0.0124 (0.0083 to 0.0165) |
| Niue | 54 (40 to 69) | 16.67 (13.04 to 20.83) | 117 (88 to 147) | 16.65 (12.92 to 21) | 0.042 (0.0352 to 0.0488) |
| North Macedonia | 1611 (1211 to 2112) | 12.73 (9.73 to 16.27) | 1297 (1020 to 1614) | 12.65 (9.74 to 16.01) | -0.0027 (-0.0086 to 0.0032) |
| Northern Mariana Islands | 2846 (2207 to 3593) | 12.36 (9.47 to 15.71) | 1964 (1554 to 2471) | 12.46 (9.64 to 15.79) | -0.0677 (-0.0883 to -0.0471) |
| Norway | 761 (621 to 918) | 14.2 (11.62 to 17.31) | 1118 (1023 to 1171) | 21.28 (19.39 to 22.28) | 0.0038 (-0.0005 to 0.0082) |
| Oman | 562 (426 to 726) | 12.38 (9.49 to 15.77) | 870 (660 to 1113) | 12.27 (9.36 to 15.47) | -0.0295 (-0.0385 to -0.0205) |
| Pakistan | 350 (266 to 447) | 13.52 (10.47 to 17.12) | 602 (459 to 771) | 13.65 (10.44 to 17.42) | -0.1049 (-0.1137 to -0.096) |
| Palau | 13488 (10178 to 17097) | 18.7 (14.59 to 23.29) | 18898 (14923 to 23487) | 18.96 (14.88 to 23.53) | 0.0455 (0.03 to 0.061) |
| Palestine | 610 (466 to 779) | 12.31 (9.46 to 15.59) | 361 (281 to 453) | 12.49 (9.39 to 15.98) | -0.0692 (-0.0823 to -0.0562) |
| Panama | 1274 (987 to 1602) | 12.8 (9.69 to 16.32) | 1109 (878 to 1391) | 12.87 (9.98 to 16.47) | 0.0224 (0.0153 to 0.0295) |
| Papua New Guinea | 4883 (3686 to 6288) | 13.88 (10.76 to 17.65) | 12222 (9257 to 15785) | 13.74 (10.82 to 17.53) | 0.0073 (-0.0064 to 0.0211) |
| Paraguay | 1319 (984 to 1709) | 13.7 (10.59 to 17.32) | 2128 (1621 to 2724) | 13.52 (10.56 to 17.15) | -0.0129 (-0.0226 to -0.0033) |
| Peru | 7707 (5823 to 9876) | 14.41 (11.09 to 18.22) | 6909 (5429 to 8683) | 14.41 (11.13 to 18.48) | 0.0188 (0.0087 to 0.0289) |
| Philippines | 5523 (4507 to 6694) | 14.02 (11.63 to 16.9) | 8716 (7158 to 10523) | 13.95 (11.51 to 16.76) | 0.0427 (0.019 to 0.0665) |
| Poland | 133991 (108496 to 164227) | 15.16 (12.46 to 18.27) | 243679 (198452 to 295372) | 15.32 (12.55 to 18.6) | 0.0269 (0.0214 to 0.0323) |
| Portugal | 6630 (5368 to 7968) | 14.76 (12.3 to 17.62) | 16488 (13297 to 19790) | 14.55 (12.15 to 17.37) | -0.0021 (-0.0088 to 0.0046) |
| Puerto Rico | 16873 (13540 to 20453) | 15.89 (13 to 19.17) | 39703 (32125 to 48183) | 15.53 (12.76 to 18.78) | 0.0178 (0.0097 to 0.026) |
| Qatar | 1280 (962 to 1658) | 14.01 (11.1 to 17.78) | 2735 (2065 to 3529) | 13.89 (10.97 to 17.68) | 0.0387 (0.0257 to 0.0517) |
| Republic of Korea | 1487 (1159 to 1863) | 14.43 (11.23 to 18.19) | 1226 (972 to 1534) | 14.35 (11.03 to 18.15) | 0.0036 (-0.0012 to 0.0084) |
| Republic of Moldova | 13101 (10713 to 15812) | 15.37 (12.9 to 18.38) | 34527 (28212 to 41511) | 15.46 (12.94 to 18.49) | -0.0181 (-0.0235 to -0.0127) |
| Romania | 41 (33 to 49) | 14.93 (12.27 to 17.9) | 40 (31 to 49) | 14.04 (10.85 to 17.84) | 0.0272 (0.021 to 0.0333) |
| Russian Federation | 802 (605 to 1023) | 14.12 (11.08 to 17.73) | 1253 (950 to 1607) | 14.07 (10.98 to 17.85) | 0.3454 (0.2788 to 0.4121) |
| Rwanda | 3760 (2775 to 4844) | 14.12 (10.88 to 17.79) | 5439 (4133 to 6893) | 14.01 (10.64 to 17.76) | -0.0123 (-0.0177 to -0.0069) |
| Saint Kitts and Nevis | 323 (241 to 413) | 13.89 (10.87 to 17.33) | 781 (599 to 1001) | 13.83 (10.75 to 17.68) | 0.021 (0.0176 to 0.0244) |
| Saint Lucia | 3533 (3028 to 4143) | 21.76 (18.69 to 25.47) | 3233 (2712 to 3860) | 21.03 (17.37 to 25.27) | 0.006 (-0.0017 to 0.0138) |
| Saint Vincent and the Grenadines | 3518 (3135 to 3977) | 20.03 (17.95 to 22.5) | 4561 (4021 to 5203) | 20.1 (17.78 to 22.49) | -0.0123 (-0.0159 to -0.0086) |
| Samoa | 4 (3 to 5) | 15.02 (11.7 to 18.98) | 4 (3 to 5) | 15.07 (11.66 to 19.1) | 0.0057 (-0.002 to 0.0133) |
| San Marino | 2 (1 to 2) | 17.57 (13.69 to 22.16) | 2 (2 to 3) | 17.2 (13.21 to 21.85) | -0.0157 (-0.0196 to -0.0118) |
| Sao Tome and Principe | 573 (432 to 732) | 13.14 (10.19 to 16.57) | 1465 (1115 to 1884) | 13.01 (10.06 to 16.62) | -0.0238 (-0.0299 to -0.0177) |
| Saudi Arabia | 5 (4 to 7) | 12.58 (9.75 to 15.92) | 8 (6 to 10) | 12.73 (9.86 to 16.17) | -0.0341 (-0.0449 to -0.0233) |
| Senegal | 1459 (1144 to 1850) | 14.44 (11.28 to 18.38) | 1455 (1143 to 1821) | 14.37 (11.01 to 18.15) | -0.0203 (-0.026 to -0.0147) |
| Serbia | 8642 (7102 to 10505) | 14 (11.61 to 16.98) | 12007 (10019 to 14671) | 13.96 (11.61 to 17.06) | -0.0048 (-0.0102 to 0.0006) |
| Seychelles | 22094 (17891 to 26817) | 13.76 (11.33 to 16.6) | 31484 (26105 to 38044) | 13.76 (11.38 to 16.59) | 0.0804 (0.0572 to 0.1036) |
| Sierra Leone | 37 (28 to 48) | 17.39 (13.6 to 21.62) | 113 (88 to 142) | 18.02 (13.91 to 22.52) | -0.0516 (-0.0597 to -0.0435) |
| Singapore | 131 (99 to 168) | 14.49 (11.43 to 18.28) | 296 (225 to 382) | 14.44 (11.36 to 18.26) | -0.0002 (-0.0077 to 0.0072) |
| Slovakia | 7 (5 to 9) | 16.85 (13.16 to 21.19) | 10 (8 to 13) | 16.82 (13.02 to 21.32) | 0.0296 (0.0234 to 0.0358) |
| Slovenia | 497 (365 to 643) | 13.31 (10.17 to 16.91) | 977 (743 to 1263) | 13.3 (10.21 to 16.98) | 0.014 (0.0063 to 0.0217) |
| Solomon Islands | 1224 (943 to 1559) | 12.4 (9.47 to 15.9) | 1016 (793 to 1272) | 12.53 (9.51 to 16.04) | -0.0035 (-0.0171 to 0.01) |
| Somalia | 11 (8 to 14) | 15.3 (11.62 to 19.18) | 8 (6 to 10) | 15.18 (11.64 to 19.01) | -0.0206 (-0.0277 to -0.0134) |
| South Africa | 740 (569 to 946) | 12.07 (9.44 to 15.12) | 1656 (1286 to 2085) | 11.95 (9.35 to 15.03) | -0.0077 (-0.0115 to -0.0039) |
| South Sudan | 38 (28 to 49) | 12.73 (9.91 to 16.26) | 53 (41 to 67) | 12.59 (9.7 to 16.07) | -0.0015 (-0.0101 to 0.0071) |
| Spain | 256 (197 to 328) | 12.57 (9.67 to 16.16) | 210 (164 to 263) | 12.71 (9.67 to 16.36) | 0.0472 (0.0189 to 0.0754) |
| Sri Lanka | 2805 (2114 to 3599) | 14.81 (11.4 to 18.67) | 5018 (3743 to 6376) | 14.33 (10.85 to 18.08) | 0.0739 (0.0437 to 0.1042) |
| Sudan | 16 (12 to 20) | 14.76 (11.44 to 18.62) | 34 (26 to 43) | 14.7 (11.49 to 18.5) | -0.0488 (-0.0621 to -0.0355) |
| Suriname | 303 (233 to 381) | 11.71 (8.93 to 14.85) | 179 (142 to 226) | 11.77 (9.02 to 14.94) | -0.0186 (-0.0212 to -0.016) |
| Sweden | 2245 (1686 to 2876) | 15.25 (11.64 to 19.41) | 2932 (2255 to 3732) | 15.3 (11.69 to 19.49) | 0.0073 (-0.0138 to 0.0284) |
| Switzerland | 16601 (13670 to 20079) | 10.91 (9.01 to 13.12) | 14842 (12311 to 17922) | 11.75 (9.71 to 14.11) | -0.0075 (-0.02 to 0.005) |
| Syrian Arab Republic | 1607 (1212 to 2057) | 16.43 (12.88 to 20.74) | 3076 (2373 to 3875) | 16.68 (12.99 to 20.93) | -0.0827 (-0.0961 to -0.0694) |
| Taiwan (Province of China) | 174 (132 to 222) | 13.78 (10.73 to 17.41) | 375 (290 to 481) | 13.88 (10.82 to 17.68) | 0.1173 (0.0915 to 0.1431) |
| Tajikistan | 19211 (16012 to 22886) | 15.41 (12.71 to 18.46) | 15043 (12447 to 18174) | 15.58 (12.88 to 18.76) | -0.0425 (-0.0502 to -0.0348) |
| Thailand | 2642 (1965 to 3433) | 12.37 (9.54 to 15.7) | 4449 (3406 to 5699) | 12.36 (9.44 to 15.72) | 0.0782 (0.0525 to 0.1039) |
| Timor-Leste | 8385 (6550 to 10483) | 14.28 (11.06 to 17.73) | 8143 (6473 to 10167) | 14.23 (11.16 to 18.06) | 0.056 (0.0285 to 0.0835) |
| Togo | 279 (209 to 359) | 14.7 (11.47 to 18.73) | 626 (475 to 794) | 14.7 (11.53 to 18.42) | -0.0247 (-0.034 to -0.0153) |
| Tokelau | 1030 (767 to 1335) | 14.01 (10.87 to 17.88) | 2784 (2105 to 3568) | 13.97 (10.9 to 17.82) | 0.0534 (0.0442 to 0.0626) |
| Tonga | 3541 (3303 to 3869) | 12.57 (11.8 to 13.61) | 4185 (3890 to 4572) | 13.22 (12.4 to 14.29) | 0.0086 (-0.0026 to 0.0198) |
| Trinidad and Tobago | 958 (718 to 1251) | 12.41 (9.58 to 15.76) | 1490 (1136 to 1899) | 12.56 (9.62 to 15.88) | 0.5858 (0.3375 to 0.8346) |
| Tunisia | 2166 (1651 to 2814) | 12.47 (9.63 to 16.01) | 2323 (1799 to 2943) | 12.57 (9.63 to 15.97) | -0.0448 (-0.0533 to -0.0363) |
| Turkey | 11274 (8396 to 14344) | 17.05 (13.14 to 21.55) | 10658 (8369 to 13122) | 17.19 (13.35 to 21.64) | -0.0252 (-0.0312 to -0.0192) |
| Turkmenistan | 14 (11 to 17) | 12.71 (9.73 to 16.2) | 9 (7 to 11) | 12.85 (9.86 to 16.47) | 0.0271 (0.0148 to 0.0394) |
| Tuvalu | 2608 (1923 to 3351) | 14.2 (10.72 to 17.91) | 6422 (4898 to 8182) | 14.1 (10.84 to 17.76) | 0.0565 (0.0509 to 0.0622) |
| Uganda | 357 (266 to 462) | 15.27 (11.61 to 19.33) | 1483 (1118 to 1942) | 14.8 (11.22 to 18.89) | -0.0119 (-0.0162 to -0.0075) |
| Ukraine | 353 (269 to 455) | 13.72 (10.86 to 17.26) | 735 (557 to 934) | 13.64 (10.79 to 17.05) | -0.0255 (-0.0306 to -0.0205) |
| United Arab Emirates | 141 (107 to 179) | 17.25 (13.36 to 21.69) | 164 (129 to 206) | 17.35 (13.61 to 21.84) | -0.0973 (-0.1064 to -0.0883) |
| United Kingdom | 616 (465 to 790) | 14.69 (11.36 to 18.51) | 1889 (1431 to 2420) | 14.59 (11.39 to 18.54) | -0.2504 (-0.3531 to -0.1476) |
| United Republic of Tanzania | 7091 (5436 to 9027) | 16.45 (12.95 to 20.52) | 9937 (7750 to 12455) | 16.65 (13.06 to 20.8) | 0.0014 (-0.0062 to 0.009) |
| United States of America | 1397 (1050 to 1831) | 13.36 (10.25 to 17.13) | 2557 (1955 to 3292) | 13.3 (10.22 to 16.95) | 0.0333 (0.0254 to 0.0412) |
| United States Virgin Islands | 12591 (10117 to 15341) | 13.92 (11.49 to 16.73) | 19105 (15711 to 23000) | 13.93 (11.44 to 16.78) | -0.1135 (-0.17 to -0.057) |
| Uruguay | 982 (741 to 1261) | 13.06 (10.04 to 16.7) | 2276 (1738 to 2928) | 13.01 (10.12 to 16.57) | -0.0197 (-0.0291 to -0.0103) |
| Uzbekistan | 477 (356 to 620) | 12.44 (9.63 to 15.96) | 687 (522 to 872) | 12.58 (9.61 to 15.88) | -0.0112 (-0.0217 to -0.0007) |
| Vanuatu | 2729 (2040 to 3524) | 13.5 (10.38 to 17.26) | 3367 (2632 to 4209) | 13.3 (10.19 to 16.83) | 0.0081 (-0.0053 to 0.0214) |
| Venezuela (Bolivarian Republic of) | 736 (622 to 877) | 14.49 (12.14 to 17.19) | 654 (520 to 813) | 13.68 (10.69 to 17.2) | -0.0317 (-0.0403 to -0.0232) |
| Viet Nam | 3724 (2751 to 4801) | 14.33 (11.12 to 18.19) | 6299 (4841 to 8089) | 14.18 (10.82 to 18.18) | 0.0871 (0.0661 to 0.1081) |
| Yemen | 11427 (9020 to 14122) | 13.8 (10.97 to 17.12) | 10196 (8080 to 12574) | 13.8 (10.77 to 17.16) | -0.0751 (-0.0833 to -0.067) |
| Zambia | 16247 (12153 to 20897) | 14.97 (11.36 to 18.9) | 26140 (19829 to 33047) | 14.52 (11.1 to 18.35) | -0.0055 (-0.0108 to -0.0001) |
| Zimbabwe | 2165 (1628 to 2772) | 14 (11.01 to 17.76) | 5802 (4332 to 7443) | 13.93 (10.87 to 17.45) | -0.0403 (-0.0471 to -0.0335) |

**Supplementary Table S3** The cases of prevalence and ASPR of schizophrenia in 1990 and 2021, and its temporal trends from 1990-2021 among 204 countries and territories.

| Characteristics | 1990 | | 2021 | | 1990-2021  EAPC (95%CI) |
| --- | --- | --- | --- | --- | --- |
|  | **Cases of prevalence (95%UI)** | **ASPR per 100,000 (95%UI)** | **Cases of prevalence (95%UI)** | **ASPR per 100,000 (95%UI)** |  |
| Afghanistan | 15403 (11993 to 19137) | 221.31 (173.56 to 280.24) | 49199 (36931 to 64346) | 218.33 (168.88 to 278.4) | -0.0223 (-0.0576 to 0.013) |
| Albania | 6499 (4977 to 8438) | 216.51 (168.48 to 276.96) | 6849 (5396 to 8591) | 221.34 (173.4 to 281.9) | 0.0757 (0.068 to 0.0833) |
| Algeria | 47257 (35919 to 60349) | 248.48 (195.62 to 311.92) | 113233 (88123 to 144217) | 246.67 (193.01 to 312.21) | -0.0178 (-0.0227 to -0.013) |
| American Samoa | 121 (91 to 156) | 301.29 (230.44 to 378.27) | 145 (112 to 181) | 292.21 (223.19 to 367.78) | -0.0821 (-0.0939 to -0.0703) |
| Andorra | 179 (138 to 225) | 273.82 (214.92 to 340.96) | 311 (248 to 389) | 271.42 (212.91 to 343.81) | -0.0287 (-0.0361 to -0.0214) |
| Angola | 16374 (12168 to 21268) | 218.91 (170.22 to 276.26) | 52167 (39189 to 67970) | 223.96 (172.96 to 283.56) | 0.1166 (0.0958 to 0.1374) |
| Antigua and Barbuda | 132 (100 to 171) | 233.05 (179.67 to 297.62) | 247 (192 to 312) | 234.12 (180.95 to 295.93) | 0.0223 (0.0114 to 0.0332) |
| Argentina | 88383 (68830 to 111524) | 277.33 (215.92 to 350.51) | 138110 (107232 to 172805) | 277.59 (215.91 to 348.36) | -0.0052 (-0.0128 to 0.0025) |
| Armenia | 7355 (5641 to 9423) | 217.7 (168.26 to 277.18) | 7700 (6065 to 9693) | 220.29 (170.76 to 279.37) | 0.0606 (0.0496 to 0.0717) |
| Australia | 71416 (65728 to 76938) | 388.26 (357.6 to 418.49) | 112864 (103875 to 121735) | 388.26 (358.09 to 419.41) | 0.0065 (0.0046 to 0.0084) |
| Austria | 24009 (19041 to 29953) | 265.94 (211.22 to 335.58) | 29477 (23379 to 36375) | 266.03 (210.06 to 335.72) | -0.0025 (-0.0095 to 0.0044) |
| Azerbaijan | 14538 (11211 to 18909) | 217.23 (168.91 to 276.64) | 27071 (20931 to 34460) | 220.74 (170.56 to 279.96) | 0.0987 (0.062 to 0.1353) |
| Bahamas | 556 (424 to 726) | 233.08 (180.32 to 295.86) | 1018 (785 to 1281) | 230.51 (178.16 to 291.77) | -0.0226 (-0.0291 to -0.0161) |
| Bahrain | 1376 (1016 to 1820) | 261.89 (205 to 334.29) | 5084 (3831 to 6485) | 263 (202.71 to 332.55) | 0.0158 (0.0136 to 0.018) |
| Bangladesh | 229499 (173871 to 293277) | 290.91 (225.82 to 365.54) | 475978 (365130 to 600224) | 285.29 (220.53 to 358.33) | -0.0838 (-0.0902 to -0.0774) |
| Barbados | 677 (553 to 812) | 261.71 (214.71 to 313.41) | 888 (697 to 1101) | 248.17 (190.49 to 310.26) | -0.0568 (-0.0886 to -0.0249) |
| Belarus | 23205 (17880 to 29221) | 202.1 (154.91 to 255.59) | 23200 (18198 to 28956) | 205.11 (157.88 to 260.44) | 0.0691 (0.0541 to 0.0842) |
| Belgium | 30797 (24582 to 38293) | 265.03 (208.1 to 333.61) | 35844 (28869 to 44609) | 264.11 (210.54 to 336.65) | -0.0062 (-0.0132 to 0.0008) |
| Belize | 295 (224 to 385) | 220.74 (172.68 to 280.31) | 932 (725 to 1177) | 221.32 (173.02 to 277.39) | -0.0044 (-0.0112 to 0.0024) |
| Benin | 7347 (5527 to 9524) | 228.87 (178.14 to 287.16) | 22505 (17115 to 29239) | 229.89 (178.5 to 289.65) | 0.0104 (0.0041 to 0.0167) |
| Bermuda | 169 (130 to 217) | 242.84 (187.73 to 308.01) | 194 (152 to 244) | 245.34 (190.62 to 310.65) | 0.053 (0.043 to 0.063) |
| Bhutan | 1375 (1042 to 1758) | 293.41 (226.56 to 369.17) | 2409 (1831 to 3059) | 296.32 (229.94 to 372.69) | 0.0199 (0.0157 to 0.0242) |
| Bolivia (Plurinational State of) | 11382 (8768 to 14568) | 230.18 (182.52 to 290.37) | 27364 (21213 to 35289) | 233.83 (182.47 to 298.95) | 0.0426 (0.0329 to 0.0523) |
| Bosnia and Herzegovina | 10388 (8077 to 13253) | 212.37 (165.37 to 270.49) | 8825 (6958 to 10972) | 221.61 (171.21 to 280.54) | 0.1442 (0.124 to 0.1645) |
| Botswana | 2062 (1577 to 2664) | 219.15 (170.95 to 277.74) | 5638 (4351 to 7243) | 228.13 (179.36 to 288.15) | 0.1351 (0.1241 to 0.1462) |
| Brazil | 318174 (260160 to 382515) | 241 (199.86 to 286.74) | 607171 (504438 to 720997) | 243.37 (202.19 to 289.75) | 0.0383 (0.0301 to 0.0464) |
| Brunei Darussalam | 626 (471 to 821) | 265.08 (209.74 to 341.89) | 1394 (1099 to 1758) | 259.64 (206.53 to 324.95) | -0.0449 (-0.0511 to -0.0387) |
| Bulgaria | 21328 (16799 to 26586) | 215.35 (168.24 to 273.77) | 18057 (14491 to 22425) | 218.27 (170.37 to 276.79) | 0.0447 (0.0348 to 0.0546) |
| Burkina Faso | 14031 (10858 to 18026) | 222.34 (176.52 to 283.74) | 36564 (27871 to 47401) | 223.39 (176.9 to 284.44) | 0.0079 (0.0044 to 0.0115) |
| Burundi | 8304 (6272 to 10949) | 212.07 (166.93 to 272.57) | 20558 (15881 to 26626) | 206.6 (161.7 to 263.86) | -0.0733 (-0.0775 to -0.0692) |
| Cabo Verde | 576 (436 to 753) | 239.41 (185.96 to 306.32) | 1441 (1109 to 1850) | 240.85 (188.38 to 304.82) | 0.0263 (0.0217 to 0.031) |
| Cambodia | 19509 (14781 to 25016) | 256.02 (197.19 to 321.16) | 45820 (34575 to 58745) | 267.19 (202.81 to 338.15) | 0.1919 (0.1562 to 0.2277) |
| Cameroon | 16794 (12771 to 21896) | 228.55 (179.86 to 292.75) | 55715 (42427 to 72421) | 226.55 (176.71 to 286.17) | -0.0203 (-0.0256 to -0.0151) |
| Canada | 89321 (88195 to 90356) | 287.97 (284.41 to 291.36) | 125956 (124158 to 127804) | 288.17 (284.43 to 291.67) | 0.0017 (0.0004 to 0.0031) |
| Central African Republic | 4134 (3136 to 5400) | 203.85 (157.98 to 259.57) | 8638 (6559 to 11175) | 199.29 (154.55 to 254.65) | -0.0693 (-0.0748 to -0.0637) |
| Chad | 9001 (6949 to 11704) | 222.87 (174.65 to 282.02) | 25097 (19055 to 32502) | 224.75 (175.71 to 283.57) | 0.0446 (0.037 to 0.0522) |
| Chile | 35646 (27652 to 45415) | 278.16 (218.18 to 351.22) | 61024 (47993 to 77296) | 281.45 (219.5 to 357.28) | 0.0389 (0.0305 to 0.0473) |
| China | 3558619 (3076267 to 4080968) | 300.81 (260.98 to 343.19) | 5322430 (4637003 to 6043640) | 312.36 (271.69 to 356.39) | 0.0673 (0.0383 to 0.0964) |
| Colombia | 67545 (51868 to 86877) | 240.12 (187.67 to 302.78) | 131170 (102534 to 166333) | 242 (189.15 to 307.48) | 0.0282 (0.0169 to 0.0396) |
| Comoros | 730 (551 to 947) | 226.13 (176.33 to 285.82) | 1582 (1229 to 2053) | 224.19 (176.23 to 288.35) | -0.0155 (-0.0198 to -0.0113) |
| Congo | 3807 (2906 to 4903) | 218.91 (169.82 to 274.2) | 10740 (8367 to 13750) | 219.91 (173.85 to 279.04) | 0.0446 (0.0278 to 0.0614) |
| Cook Islands | 50 (38 to 63) | 300.78 (228.53 to 376.6) | 59 (46 to 73) | 306.12 (235.53 to 385.58) | 0.0518 (0.0406 to 0.063) |
| Costa Rica | 6360 (4882 to 8252) | 244.28 (193.17 to 308.11) | 13177 (10325 to 16660) | 246.45 (192.7 to 311.86) | 0.0318 (0.0249 to 0.0387) |
| Croatia | 12545 (9814 to 15859) | 222.7 (172.55 to 284.45) | 11357 (8940 to 14136) | 225.66 (174.53 to 285.33) | 0.0462 (0.0392 to 0.0532) |
| Cuba | 26019 (20059 to 33035) | 230.73 (178.59 to 290.55) | 31151 (24614 to 38800) | 228.78 (179.41 to 285.66) | 0.0012 (-0.0204 to 0.0228) |
| Cyprus | 2177 (1729 to 2723) | 265.24 (210.48 to 331.63) | 4533 (3577 to 5682) | 265.8 (209.55 to 332.88) | -0.0101 (-0.0155 to -0.0048) |
| Czechia | 25869 (20268 to 32776) | 225.99 (174.16 to 287.99) | 29112 (23256 to 36251) | 228.86 (176.9 to 292.22) | 0.0251 (0.0191 to 0.0311) |
| C么te d'Ivoire | 19598 (14849 to 25821) | 225.57 (174.34 to 287.44) | 51009 (38651 to 66048) | 226.54 (175.51 to 286.63) | -0.0096 (-0.0234 to 0.0043) |
| Democratic People's Republic of Korea | 56820 (43946 to 72014) | 273.55 (212.49 to 343.8) | 82645 (64323 to 103893) | 262.5 (204.38 to 330.74) | -0.1691 (-0.1834 to -0.1548) |
| Democratic Republic of the Congo | 57984 (44483 to 75328) | 215.14 (168.35 to 273.46) | 145659 (110905 to 187083) | 208.48 (161.09 to 260.7) | -0.0968 (-0.1296 to -0.064) |
| Denmark | 14949 (12510 to 17763) | 247.07 (205.26 to 295.2) | 24415 (21794 to 26762) | 362.11 (322.98 to 394.8) | 1.7412 (1.4207 to 2.0626) |
| Djibouti | 705 (529 to 916) | 224.36 (174.7 to 282.3) | 2760 (2118 to 3538) | 223.16 (174.25 to 280.99) | -0.0044 (-0.0144 to 0.0056) |
| Dominica | 139 (107 to 178) | 224.12 (174.36 to 283.46) | 170 (133 to 211) | 225.23 (175.82 to 282.8) | 0.0192 (0.0134 to 0.0251) |
| Dominican Republic | 12906 (9720 to 16742) | 221.18 (171.57 to 278.39) | 25873 (20061 to 32923) | 227.74 (178.23 to 288.08) | 0.1005 (0.0948 to 0.1062) |
| Ecuador | 19211 (14689 to 25081) | 237.39 (184.21 to 302.2) | 43346 (34250 to 55004) | 238.35 (189.57 to 301.18) | 0.0295 (0.0199 to 0.0392) |
| Egypt | 109645 (83881 to 140797) | 240.16 (187.62 to 304.94) | 238661 (182299 to 303933) | 242.74 (187.98 to 305.36) | 0.0333 (0.0256 to 0.0409) |
| El Salvador | 9320 (7142 to 11932) | 228.58 (179.62 to 287.14) | 14677 (11474 to 18572) | 230.92 (181.04 to 291.79) | 0.0368 (0.0303 to 0.0434) |
| Equatorial Guinea | 640 (495 to 830) | 213.91 (167.26 to 271.07) | 2844 (2157 to 3679) | 234.44 (182.37 to 297.44) | 0.4352 (0.3585 to 0.5119) |
| Eritrea | 5127 (3905 to 6596) | 212.99 (167.02 to 267.76) | 12141 (9197 to 15628) | 214.82 (166.39 to 272.08) | 0.0195 (-0.0031 to 0.0421) |
| Estonia | 3559 (2814 to 4477) | 204.51 (159.86 to 258.41) | 3231 (2577 to 4092) | 210.26 (163.64 to 269.16) | 0.0991 (0.0916 to 0.1065) |
| Eswatini | 1172 (883 to 1516) | 217.43 (167.93 to 277.92) | 2260 (1707 to 2936) | 220.88 (171.08 to 279.18) | 0.0598 (0.0503 to 0.0693) |
| Ethiopia | 76263 (62342 to 92089) | 218.9 (182.54 to 260.37) | 189550 (154158 to 229567) | 222.73 (184.02 to 265.41) | 0.0786 (0.063 to 0.0943) |
| Fiji | 1889 (1417 to 2403) | 283.39 (217.53 to 354.05) | 2693 (2096 to 3406) | 287.05 (223.84 to 362.22) | 0.0423 (0.0289 to 0.0556) |
| Finland | 16472 (13655 to 19503) | 277.6 (227.97 to 330.05) | 17415 (13919 to 21403) | 263.17 (208.82 to 326.82) | -0.1825 (-0.228 to -0.1368) |
| France | 168054 (133665 to 209082) | 260.27 (205.29 to 325.53) | 197666 (158868 to 247062) | 259.46 (202.48 to 330.58) | -0.021 (-0.028 to -0.014) |
| Gabon | 1746 (1308 to 2236) | 231.28 (177.78 to 290.73) | 3747 (2847 to 4754) | 231.71 (177.83 to 291.01) | 0.0095 (0.0045 to 0.0145) |
| Gambia | 1558 (1176 to 2006) | 226.6 (176.43 to 284.5) | 4162 (3150 to 5340) | 225.61 (175.7 to 282.71) | -0.0083 (-0.017 to 0.0004) |
| Georgia | 12809 (10048 to 16187) | 218.35 (170.46 to 277.15) | 8879 (6970 to 11211) | 218.04 (169.05 to 275.47) | 0.0311 (-0.0002 to 0.0624) |
| Germany | 235239 (188844 to 290968) | 243.43 (194.11 to 303.9) | 253730 (203539 to 312553) | 244.3 (192.46 to 309.41) | -0.014 (-0.0314 to 0.0034) |
| Ghana | 25137 (18877 to 32331) | 227.79 (175.87 to 287.27) | 68855 (51940 to 88753) | 233.13 (181.22 to 295.36) | 0.0907 (0.0828 to 0.0987) |
| Greece | 31213 (24935 to 38294) | 263.78 (210.23 to 328.67) | 32757 (26281 to 40765) | 262.73 (203.53 to 329.52) | -0.0216 (-0.027 to -0.0162) |
| Greenland | 205 (155 to 261) | 332.26 (256.6 to 416.05) | 215 (168 to 267) | 331.14 (257.22 to 412.81) | 0.0687 (0.0295 to 0.1079) |
| Grenada | 153 (118 to 197) | 222.24 (174.66 to 281.19) | 262 (204 to 326) | 227.87 (177.32 to 286.02) | 0.078 (0.0726 to 0.0834) |
| Guam | 427 (322 to 557) | 316.69 (244.19 to 403.18) | 534 (418 to 668) | 312.78 (241.27 to 398.78) | -0.028 (-0.038 to -0.018) |
| Guatemala | 12939 (9897 to 16740) | 227.55 (178.58 to 288.65) | 33429 (25909 to 42766) | 229.07 (180.83 to 288.28) | 0.0144 (0.0067 to 0.0221) |
| Guinea | 9949 (7669 to 12674) | 226.68 (176.78 to 284.11) | 22080 (16674 to 28247) | 226.01 (175.94 to 282.3) | -0.0155 (-0.0271 to -0.004) |
| Guinea-Bissau | 1519 (1152 to 1992) | 220.91 (172.37 to 279.86) | 3476 (2636 to 4472) | 221.07 (174.53 to 282.03) | -0.02 (-0.0262 to -0.0138) |
| Guyana | 1361 (1031 to 1768) | 211.92 (165.34 to 268.14) | 1677 (1324 to 2121) | 217.19 (172.2 to 273.53) | 0.068 (0.0616 to 0.0744) |
| Haiti | 10191 (7892 to 13155) | 205.78 (161.65 to 260.26) | 24157 (18525 to 30966) | 201.67 (158.59 to 254.15) | -0.0673 (-0.076 to -0.0587) |
| Honduras | 7453 (5673 to 9521) | 229.03 (180.57 to 287) | 21172 (16263 to 26932) | 228.12 (179.46 to 286.02) | -0.0158 (-0.0243 to -0.0073) |
| Hungary | 25769 (20225 to 32479) | 219.1 (170.24 to 279.03) | 25985 (20781 to 32282) | 223.76 (173.26 to 284.04) | 0.0557 (0.0488 to 0.0626) |
| Iceland | 707 (551 to 883) | 268.87 (208.97 to 334.67) | 1085 (870 to 1352) | 269.02 (212.94 to 338.99) | -0.0048 (-0.0107 to 0.0011) |
| India | 2083453 (1715455 to 2473528) | 286.49 (238 to 336.77) | 4373183 (3639071 to 5178753) | 296.22 (246.51 to 349.92) | 0.1301 (0.0951 to 0.1651) |
| Indonesia | 452063 (367908 to 540858) | 278.85 (231.14 to 331.58) | 888193 (736989 to 1054080) | 286.49 (238.1 to 339.27) | 0.1327 (0.0986 to 0.1669) |
| Iran (Islamic Republic of) | 104416 (85019 to 125708) | 248.75 (206.29 to 296.42) | 250269 (206659 to 296635) | 250.71 (207.03 to 297.11) | 0.0285 (0.0232 to 0.0337) |
| Iraq | 32397 (24107 to 41705) | 243.67 (188.79 to 308.36) | 95671 (72994 to 122570) | 244.12 (189.02 to 307.12) | 0.0383 (0.0185 to 0.0581) |
| Ireland | 11519 (9428 to 13788) | 323.25 (264.38 to 387.09) | 18283 (14180 to 22576) | 322.51 (246.67 to 403.06) | 0.1254 (0.0421 to 0.2087) |
| Israel | 12632 (10046 to 15759) | 271.97 (216.76 to 339.12) | 25804 (20397 to 32251) | 266.62 (210.03 to 335.44) | -0.0445 (-0.0608 to -0.0283) |
| Italy | 168049 (142985 to 195424) | 251.79 (212.09 to 295.32) | 186299 (160315 to 215636) | 251.9 (212.48 to 296.66) | -0.0038 (-0.0068 to -0.0009) |
| Jamaica | 4475 (3431 to 5702) | 226.43 (175.41 to 285.45) | 7043 (5456 to 8881) | 226.3 (175.34 to 284.91) | -0.011 (-0.0152 to -0.0067) |
| Japan | 416435 (348737 to 487572) | 283.33 (236.55 to 333.33) | 409399 (348029 to 474911) | 279.02 (232.22 to 332.33) | 0.1057 (0.009 to 0.2025) |
| Jordan | 6690 (5009 to 8785) | 249.47 (194.15 to 317.97) | 30680 (23552 to 39264) | 248.82 (194.86 to 314.85) | -0.0113 (-0.0216 to -0.0009) |
| Kazakhstan | 34018 (26325 to 43741) | 215.39 (166.94 to 274.21) | 44322 (34717 to 56439) | 220.75 (173.13 to 279.94) | 0.111 (0.089 to 0.1329) |
| Kenya | 33832 (27558 to 41005) | 222.05 (184.6 to 264.39) | 94216 (77156 to 113436) | 223.14 (185.7 to 265.81) | 0.015 (0.0088 to 0.0212) |
| Kiribati | 161 (121 to 209) | 260.8 (199.98 to 330.73) | 289 (221 to 367) | 256.57 (197.84 to 325.81) | -0.0485 (-0.0612 to -0.0358) |
| Kuwait | 4734 (3446 to 6229) | 268.32 (204.31 to 339.13) | 16561 (12597 to 21280) | 265.73 (206.19 to 337.41) | -0.0393 (-0.0535 to -0.0252) |
| Kyrgyzstan | 7939 (6158 to 10172) | 211.19 (164.13 to 266.44) | 13861 (10643 to 17768) | 208.81 (162.23 to 265.38) | -0.0312 (-0.053 to -0.0094) |
| Lao People's Democratic Republic | 8209 (6129 to 10527) | 260.13 (198.67 to 329.35) | 19871 (15203 to 24996) | 273.3 (212.26 to 340.14) | 0.2249 (0.1905 to 0.2594) |
| Latvia | 6053 (4715 to 7683) | 203.72 (158.8 to 260.39) | 4570 (3665 to 5643) | 207.79 (160.53 to 263.33) | 0.0925 (0.0815 to 0.1034) |
| Lebanon | 6667 (5232 to 8465) | 250.21 (195.11 to 316.08) | 15639 (12011 to 19917) | 250.49 (193.78 to 317.83) | 0.0048 (-0.001 to 0.0106) |
| Lesotho | 2312 (1795 to 2936) | 203.73 (160.68 to 256.54) | 3528 (2670 to 4553) | 209.16 (163.39 to 264.62) | 0.1017 (0.0923 to 0.1111) |
| Liberia | 3981 (2995 to 5210) | 224.06 (172.76 to 286.52) | 9906 (7583 to 12705) | 220.25 (171.04 to 278.02) | -0.022 (-0.0377 to -0.0063) |
| Libya | 7986 (5954 to 10346) | 256.4 (196.52 to 323.54) | 19836 (15176 to 25346) | 245.85 (188.86 to 312.4) | -0.146 (-0.1673 to -0.1247) |
| Lithuania | 8161 (6295 to 10249) | 203.35 (156.98 to 256.39) | 6748 (5410 to 8530) | 209.51 (164.07 to 264.29) | 0.0984 (0.0898 to 0.107) |
| Luxembourg | 1236 (974 to 1550) | 269.27 (211.31 to 336.47) | 2192 (1737 to 2745) | 271.58 (211.27 to 341.73) | 0.027 (0.0204 to 0.0335) |
| Madagascar | 18428 (14026 to 23842) | 218.32 (171.23 to 273.68) | 49224 (37278 to 63697) | 217.68 (169.13 to 274.6) | -0.0028 (-0.0104 to 0.0048) |
| Malawi | 14285 (10847 to 18669) | 207.72 (162.25 to 266.19) | 30257 (22786 to 39390) | 206.77 (161.15 to 264.04) | 0.0084 (-0.0019 to 0.0188) |
| Malaysia | 45291 (34353 to 58900) | 292.69 (226.99 to 370.59) | 105811 (81281 to 136144) | 302.99 (233.52 to 387.34) | 0.1608 (0.1329 to 0.1887) |
| Maldives | 451 (342 to 586) | 292.36 (228.23 to 366.88) | 2063 (1565 to 2632) | 308.46 (237.95 to 390.33) | 0.2291 (0.1901 to 0.2681) |
| Mali | 13562 (10403 to 17366) | 222.54 (173.06 to 280.92) | 36425 (27602 to 47064) | 226.36 (177.18 to 285.12) | 0.0498 (0.0453 to 0.0544) |
| Malta | 1091 (860 to 1372) | 264.42 (208.5 to 334.14) | 1443 (1148 to 1790) | 267.72 (210.46 to 335.98) | 0.0236 (0.0153 to 0.032) |
| Marshall Islands | 83 (63 to 107) | 267.47 (206.66 to 339.63) | 148 (113 to 190) | 266.62 (204.97 to 337.11) | -0.012 (-0.0229 to -0.0012) |
| Mauritania | 3470 (2637 to 4453) | 233.24 (182.26 to 294.45) | 7910 (6150 to 10167) | 235.69 (185.12 to 297.99) | 0.0314 (0.0255 to 0.0374) |
| Mauritius | 3163 (2398 to 4158) | 289.29 (223.65 to 370.27) | 4482 (3511 to 5532) | 297.12 (231.36 to 373.43) | 0.1335 (0.1091 to 0.1578) |
| Mexico | 167905 (136618 to 202960) | 247.28 (204.86 to 294.29) | 343771 (283935 to 410245) | 248.56 (205.58 to 296.67) | 0.014 (0.0066 to 0.0215) |
| Micronesia (Federated States of) | 203 (152 to 264) | 266.01 (205.21 to 337.33) | 266 (202 to 341) | 265.56 (202.96 to 337.94) | -0.0043 (-0.0128 to 0.0042) |
| Monaco | 110 (89 to 138) | 283.96 (224.63 to 362.7) | 134 (107 to 167) | 284.99 (223.29 to 364.26) | -0.0084 (-0.0213 to 0.0045) |
| Mongolia | 3381 (2533 to 4387) | 206.21 (160.16 to 260.36) | 7263 (5514 to 9196) | 213.46 (163.9 to 269.39) | 0.1313 (0.1174 to 0.1453) |
| Montenegro | 1445 (1124 to 1841) | 219.87 (171.08 to 279.75) | 1598 (1264 to 2000) | 221.14 (171.82 to 280.44) | 0.0368 (0.0285 to 0.0451) |
| Morocco | 50138 (38056 to 64727) | 239.06 (186.32 to 303.98) | 94370 (73274 to 118604) | 239.86 (186.41 to 301.4) | -0.0024 (-0.0135 to 0.0088) |
| Mozambique | 19422 (14856 to 24715) | 201.55 (160.16 to 254.94) | 45585 (34469 to 59183) | 208.21 (162.49 to 265.08) | 0.1429 (0.1299 to 0.1558) |
| Myanmar | 89132 (68290 to 114348) | 253.95 (197.56 to 319.8) | 155574 (120754 to 195859) | 269.19 (209.41 to 337.81) | 0.2806 (0.2383 to 0.3228) |
| Namibia | 2300 (1747 to 2954) | 221.89 (173.38 to 280.49) | 5048 (3810 to 6471) | 226.24 (174.11 to 287.11) | 0.0883 (0.077 to 0.0995) |
| Nauru | 25 (19 to 32) | 295.7 (228.74 to 375.64) | 28 (21 to 35) | 281.45 (215.73 to 353.99) | -0.1478 (-0.2349 to -0.0606) |
| Nepal | 42431 (32343 to 53638) | 284.05 (221.39 to 354.39) | 82908 (63527 to 104491) | 277.57 (215.13 to 345) | -0.0989 (-0.1106 to -0.0872) |
| Netherlands | 65767 (56913 to 75527) | 380.36 (329.43 to 437.87) | 74734 (62339 to 88904) | 364.86 (302.96 to 441.61) | -0.1677 (-0.2104 to -0.1249) |
| New Zealand | 13779 (11384 to 16306) | 378.71 (312.87 to 448.38) | 22083 (18695 to 25936) | 381.86 (319.64 to 450.34) | 0.0218 (0.0137 to 0.0298) |
| Nicaragua | 6258 (4689 to 8092) | 235.72 (185.89 to 297.43) | 15540 (11960 to 19975) | 235.76 (184.17 to 299.06) | 0.0096 (-0.0032 to 0.0223) |
| Niger | 11751 (8924 to 15257) | 222.51 (175.76 to 280.49) | 34151 (25799 to 43742) | 221.8 (171.24 to 278.39) | -0.0088 (-0.0155 to -0.0021) |
| Nigeria | 162126 (133406 to 194519) | 240.52 (200.53 to 286.28) | 422166 (345318 to 510658) | 248.73 (207.33 to 296.93) | 0.1506 (0.1325 to 0.1688) |
| Niue | 6 (4 to 7) | 286.67 (221.4 to 359.63) | 5 (4 to 6) | 290.41 (223.15 to 368.02) | 0.0753 (0.0654 to 0.0852) |
| North Macedonia | 4574 (3536 to 5846) | 218.99 (169.56 to 279.3) | 6037 (4763 to 7630) | 221.22 (172.8 to 281.33) | 0.0307 (0.0263 to 0.0351) |
| Northern Mariana Islands | 157 (117 to 205) | 322.26 (244.76 to 409.92) | 168 (131 to 209) | 308.9 (236.55 to 389.76) | -0.1592 (-0.1836 to -0.1348) |
| Norway | 13059 (10969 to 15327) | 273.88 (228.67 to 323.18) | 17558 (14915 to 20467) | 274.25 (230.81 to 325.38) | 0.0125 (0.0072 to 0.0178) |
| Oman | 4330 (3223 to 5624) | 256.75 (197.56 to 326.75) | 14659 (11098 to 19177) | 258 (199.45 to 329.4) | 0.0184 (0.0124 to 0.0243) |
| Pakistan | 238894 (196997 to 284422) | 295.01 (243.39 to 349.96) | 602480 (495122 to 724317) | 290.38 (240.87 to 344.82) | -0.0757 (-0.0835 to -0.0678) |
| Palau | 43 (32 to 54) | 288.81 (223.49 to 364.56) | 65 (51 to 82) | 293.72 (224.83 to 370.52) | 0.0448 (0.0279 to 0.0618) |
| Palestine | 3355 (2503 to 4408) | 243.27 (187.56 to 311.55) | 10984 (8282 to 14286) | 243.22 (188.56 to 310.07) | -0.0343 (-0.0497 to -0.019) |
| Panama | 5063 (3933 to 6504) | 243.08 (190.94 to 307.09) | 10939 (8469 to 13859) | 249.15 (192.77 to 316.16) | 0.0846 (0.0731 to 0.0962) |
| Papua New Guinea | 8877 (6707 to 11496) | 271.49 (210.59 to 342.23) | 25422 (19349 to 32869) | 273.43 (211.32 to 346.8) | 0.006 (-0.0108 to 0.0228) |
| Paraguay | 7643 (5855 to 9757) | 240.2 (187.5 to 299.61) | 17571 (13621 to 22249) | 242.72 (189.94 to 305.16) | 0.0181 (0.0071 to 0.0291) |
| Peru | 41991 (32203 to 54261) | 237.94 (185.35 to 302.17) | 92033 (71390 to 116004) | 242.5 (188.69 to 304.72) | 0.0669 (0.0554 to 0.0784) |
| Philippines | 144546 (117487 to 172695) | 280.65 (233.27 to 333.64) | 319558 (263009 to 380176) | 284.73 (236.89 to 337.52) | 0.0867 (0.0579 to 0.1154) |
| Poland | 90984 (75570 to 108351) | 220.31 (182.9 to 262.26) | 105994 (89000 to 124491) | 226.82 (188.66 to 270.46) | 0.0941 (0.089 to 0.0991) |
| Portugal | 28890 (22985 to 35628) | 258.38 (203.94 to 323.1) | 34178 (27378 to 42024) | 260.31 (205.42 to 327.74) | 0.0015 (-0.0058 to 0.0087) |
| Puerto Rico | 8468 (6625 to 10723) | 234.4 (183.66 to 296.18) | 9199 (7352 to 11487) | 236.73 (185.65 to 304.1) | 0.0385 (0.0282 to 0.0487) |
| Qatar | 1414 (1049 to 1860) | 273.24 (213.72 to 349.05) | 11814 (8815 to 15482) | 276.27 (212.05 to 354.74) | 0.0873 (0.0708 to 0.1038) |
| Republic of Korea | 118524 (90446 to 153356) | 257.35 (201.39 to 326.37) | 173644 (137978 to 214984) | 258.87 (203.3 to 324.92) | 0.0149 (0.0099 to 0.02) |
| Republic of Moldova | 9167 (7069 to 11623) | 197.86 (152.8 to 250.5) | 8987 (7077 to 11285) | 198.51 (154.4 to 249.5) | 0.0065 (-0.0112 to 0.0243) |
| Romania | 54245 (42085 to 68705) | 217.04 (167.13 to 276.78) | 49851 (39344 to 62264) | 221.61 (174.31 to 277.76) | 0.087 (0.0753 to 0.0986) |
| Russian Federation | 322860 (269208 to 383077) | 191.44 (158.97 to 228.1) | 358774 (301905 to 422537) | 207.01 (171.73 to 246.76) | 0.3768 (0.3001 to 0.4534) |
| Rwanda | 10751 (8145 to 14096) | 214.38 (166.35 to 271.57) | 24483 (18625 to 31582) | 216.56 (169.89 to 274.17) | 0.0782 (0.0597 to 0.0967) |
| Saint Kitts and Nevis | 80 (62 to 103) | 226.77 (177.46 to 286.46) | 169 (132 to 212) | 233.36 (181.48 to 295.52) | 0.0801 (0.0738 to 0.0864) |
| Saint Lucia | 246 (188 to 319) | 224.46 (176.07 to 286.83) | 488 (379 to 612) | 227.24 (176.51 to 288.47) | 0.0232 (0.0134 to 0.033) |
| Saint Vincent and the Grenadines | 193 (147 to 251) | 220.85 (172.94 to 280.24) | 284 (224 to 355) | 223.99 (176.47 to 281.73) | 0.0513 (0.0443 to 0.0583) |
| Samoa | 350 (265 to 450) | 278.92 (214.13 to 351.59) | 515 (395 to 652) | 281.06 (217.03 to 352.77) | 0.0379 (0.0316 to 0.0442) |
| San Marino | 75 (59 to 94) | 272.57 (214.44 to 344.89) | 108 (86 to 134) | 269.73 (210.46 to 339.49) | -0.0362 (-0.0436 to -0.0287) |
| Sao Tome and Principe | 191 (148 to 244) | 235.28 (185.97 to 294.64) | 450 (341 to 573) | 236.01 (182.76 to 295.68) | 0.0148 (0.0101 to 0.0194) |
| Saudi Arabia | 32839 (24669 to 42549) | 256.78 (198.37 to 323.56) | 123531 (94130 to 159015) | 256.72 (199.11 to 326.64) | -0.001 (-0.0136 to 0.0117) |
| Senegal | 11969 (9076 to 15393) | 231.63 (180.81 to 291.27) | 29281 (22338 to 37737) | 232.56 (182.72 to 294.1) | 0.0166 (0.0129 to 0.0203) |
| Serbia | 23605 (18239 to 29770) | 219.61 (169.04 to 278.35) | 23504 (18699 to 29096) | 220.34 (170.91 to 277.95) | 0.0336 (0.0195 to 0.0478) |
| Seychelles | 194 (147 to 250) | 293.73 (226.97 to 372.49) | 364 (284 to 463) | 301.89 (233.92 to 386.11) | 0.1147 (0.089 to 0.1405) |
| Sierra Leone | 7074 (5470 to 9188) | 224.79 (176.34 to 283.97) | 15685 (11667 to 20283) | 222.34 (170.16 to 278.07) | -0.0419 (-0.0538 to -0.0301) |
| Singapore | 8737 (6750 to 11260) | 252.41 (196.59 to 320.42) | 19472 (15246 to 24379) | 255.17 (200.39 to 322.59) | 0.0163 (0.0081 to 0.0246) |
| Slovakia | 12409 (9644 to 15762) | 220.93 (171.4 to 281.15) | 15099 (11931 to 19273) | 225.78 (176.81 to 290.63) | 0.0789 (0.0735 to 0.0843) |
| Slovenia | 5022 (3884 to 6389) | 224.42 (173.34 to 287.08) | 5693 (4512 to 7172) | 228.75 (177.97 to 292.74) | 0.0469 (0.0382 to 0.0557) |
| Solomon Islands | 623 (469 to 800) | 261.4 (203.01 to 330.5) | 1533 (1167 to 1976) | 259.51 (201.09 to 328.08) | -0.0385 (-0.0593 to -0.0178) |
| Somalia | 11077 (8416 to 14461) | 200.59 (157.3 to 256.1) | 29072 (21898 to 37419) | 196.06 (152.11 to 248.66) | -0.0722 (-0.0836 to -0.0608) |
| South Africa | 71308 (58309 to 86001) | 227.34 (187.98 to 270.92) | 138891 (113942 to 166760) | 228.35 (190.17 to 273.31) | 0.0441 (0.0353 to 0.0529) |
| South Sudan | 9324 (7059 to 12094) | 224.18 (177.02 to 284.5) | 15338 (11606 to 19631) | 218.19 (170.13 to 276.58) | -0.0576 (-0.0839 to -0.0313) |
| Spain | 112695 (106583 to 118017) | 264.13 (248.77 to 276.72) | 151433 (143138 to 159201) | 265.77 (249.9 to 278.89) | 0.0163 (0.0112 to 0.0213) |
| Sri Lanka | 45930 (35336 to 58553) | 281 (217.13 to 354.73) | 70049 (54736 to 87782) | 290.23 (225.3 to 366.69) | 0.1581 (0.1257 to 0.1905) |
| Sudan | 33863 (25593 to 43726) | 229.56 (175.49 to 290.8) | 85031 (64304 to 110412) | 231.44 (179.66 to 292.51) | 0.0199 (0.0107 to 0.0291) |
| Suriname | 675 (565 to 786) | 197.46 (167.18 to 231.54) | 1237 (1052 to 1454) | 198.51 (169 to 233.35) | 0.0304 (0.0215 to 0.0392) |
| Sweden | 28131 (24441 to 32092) | 278.14 (240.6 to 320.21) | 33889 (29160 to 39445) | 273.42 (231.48 to 321.08) | -0.0623 (-0.07 to -0.0546) |
| Switzerland | 22389 (17617 to 27981) | 272.73 (213.43 to 340.8) | 29838 (23779 to 37074) | 271.04 (212.87 to 339.94) | -0.0107 (-0.0236 to 0.0022) |
| Syrian Arab Republic | 21264 (15961 to 27553) | 241.95 (186.9 to 306.3) | 33171 (26211 to 41737) | 238.5 (185.97 to 302.7) | -0.0479 (-0.0603 to -0.0354) |
| Taiwan (Province of China) | 61578 (47699 to 77394) | 291.77 (230.54 to 362.27) | 93167 (73650 to 115077) | 310.3 (239.58 to 386.89) | 0.1363 (0.1086 to 0.164) |
| Tajikistan | 8417 (6374 to 10825) | 211.8 (165.17 to 268.57) | 19674 (14867 to 25060) | 208.44 (162.07 to 262.8) | -0.0388 (-0.064 to -0.0135) |
| Thailand | 156769 (118978 to 201591) | 280.28 (216.2 to 353.83) | 234879 (186113 to 292734) | 288.98 (223.94 to 366.57) | 0.1469 (0.1208 to 0.173) |
| Timor-Leste | 1568 (1192 to 2016) | 248.88 (194.97 to 311.39) | 2904 (2198 to 3718) | 253.26 (195.33 to 318.96) | 0.1552 (0.1183 to 0.1921) |
| Togo | 5520 (4123 to 7099) | 224.95 (176.74 to 281.05) | 15646 (11930 to 19965) | 223.86 (173.28 to 280.14) | -0.0178 (-0.0232 to -0.0125) |
| Tokelau | 4 (3 to 5) | 275.14 (212.88 to 344.95) | 4 (3 to 5) | 281.99 (216.14 to 361.56) | 0.0942 (0.0815 to 0.1069) |
| Tonga | 207 (160 to 266) | 282.09 (219.95 to 359.11) | 258 (196 to 324) | 283.93 (218.35 to 355.4) | 0.0185 (0.0085 to 0.0286) |
| Trinidad and Tobago | 1991 (1773 to 2242) | 182.5 (162.02 to 205.53) | 3812 (3053 to 4696) | 233.32 (183.83 to 286.77) | 0.607 (0.3945 to 0.8199) |
| Tunisia | 17388 (13227 to 22480) | 246.77 (191.15 to 312.57) | 32893 (25578 to 41930) | 247.31 (191.29 to 315.98) | -0.005 (-0.0114 to 0.0015) |
| Turkey | 123013 (101106 to 148654) | 243.88 (201.93 to 290.92) | 231008 (193585 to 273817) | 246.19 (205.15 to 293.8) | 0.0182 (0.0106 to 0.0257) |
| Turkmenistan | 6234 (4680 to 8135) | 213.59 (166.81 to 273.34) | 11473 (8786 to 14532) | 219.69 (169.44 to 275.59) | 0.1123 (0.083 to 0.1416) |
| Tuvalu | 22 (17 to 29) | 262.87 (201.79 to 332.63) | 32 (25 to 41) | 270.35 (209.58 to 338.88) | 0.0764 (0.068 to 0.0848) |
| Uganda | 23449 (17978 to 30250) | 207.4 (162.97 to 262.42) | 64061 (48498 to 83117) | 212.94 (167.85 to 269.55) | 0.1018 (0.094 to 0.1097) |
| Ukraine | 120057 (99867 to 143710) | 204.06 (168.74 to 244.24) | 107908 (90381 to 126858) | 202.22 (168.14 to 240.23) | -0.014 (-0.0315 to 0.0034) |
| United Arab Emirates | 5651 (4100 to 7440) | 275.86 (212.19 to 353.3) | 39413 (30202 to 50621) | 265.87 (207.02 to 337.08) | -0.1214 (-0.1329 to -0.1098) |
| United Kingdom | 158755 (136151 to 182990) | 244.89 (207.32 to 284.79) | 171821 (146894 to 199169) | 214.28 (180.74 to 250.43) | -0.3275 (-0.4629 to -0.1919) |
| United Republic of Tanzania | 37996 (28713 to 49326) | 214.52 (169.07 to 271.8) | 98830 (76249 to 125642) | 217.99 (169.67 to 273.18) | 0.0847 (0.0733 to 0.096) |
| United States of America | 249 (193 to 317) | 233.73 (181.84 to 296.64) | 243 (193 to 301) | 239.19 (186.95 to 302.96) | 0.0662 (0.0464 to 0.086) |
| United States Virgin Islands | 1042439 (869138 to 1231897) | 370.36 (308.78 to 437.53) | 1332389 (1126997 to 1559304) | 354.43 (298.14 to 415.84) | -0.097 (-0.147 to -0.0469) |
| Uruguay | 8855 (6991 to 11044) | 277.75 (217.83 to 349.27) | 10543 (8362 to 13155) | 278.69 (220.41 to 351.97) | -0.0003 (-0.0146 to 0.014) |
| Uzbekistan | 34447 (26524 to 44914) | 209.64 (163.45 to 265.56) | 76050 (58211 to 96818) | 212.7 (164.22 to 269) | 0.0553 (0.0382 to 0.0725) |
| Vanuatu | 305 (226 to 395) | 267.13 (203.71 to 337.12) | 726 (546 to 938) | 265.15 (204.94 to 336.15) | -0.0138 (-0.0265 to -0.0011) |
| Venezuela (Bolivarian Republic of) | 38327 (29274 to 49349) | 243.05 (191.06 to 306.7) | 69416 (54852 to 86712) | 239.03 (188.52 to 298.19) | -0.0147 (-0.0317 to 0.0024) |
| Viet Nam | 174128 (132610 to 218985) | 306.26 (234.24 to 379.05) | 356945 (278086 to 441848) | 319.48 (249.9 to 395.68) | 0.1889 (0.1666 to 0.2113) |
| Yemen | 20235 (15372 to 26257) | 230.5 (178.1 to 292.64) | 61792 (47221 to 80437) | 225.33 (176.32 to 287.01) | -0.0583 (-0.0739 to -0.0427) |
| Zambia | 11222 (8484 to 14444) | 213.41 (168.18 to 266.02) | 32640 (24655 to 42418) | 217.31 (171.4 to 274.77) | 0.0987 (0.079 to 0.1184) |
| Zimbabwe | 14946 (11105 to 19657) | 212.89 (164.77 to 272.73) | 26179 (19783 to 33484) | 207.61 (161.86 to 263.73) | -0.1201 (-0.1462 to -0.094) |

**Supplementary Table S4** The cases of DALYs and ASDR of schizophrenia in 1990 and 2021, and its temporal trends from 1990-2021 among 204 countries and territories.

| Characteristics | 1990 | | 2021 | | 1990-2021  EAPC (95%CI) |
| --- | --- | --- | --- | --- | --- |
|  | **Cases of DALYs (95%UI)** | **ASDR per 100,000 (95%UI)** | **Cases of DALYs (95%UI)** | **ASDR per 100,000 (95%UI)** |  |
| Afghanistan | 9623 (6842 to 12973) | 137.81 (97.87 to 185.29) | 31057 (21691 to 43493) | 135.78 (96.75 to 187.94) | -0.0025 (-0.045 to 0.0401) |
| Albania | 4192 (2915 to 5819) | 138.75 (97.13 to 189.22) | 4359 (3024 to 5911) | 142.1 (98.39 to 193.31) | 0.1011 (0.0891 to 0.1131) |
| Algeria | 30470 (21306 to 42410) | 158.86 (110.76 to 215.43) | 72513 (51598 to 100703) | 157.6 (112.69 to 217.68) | -0.0184 (-0.0231 to -0.0138) |
| American Samoa | 79 (55 to 111) | 194.2 (136.35 to 267.42) | 92 (65 to 125) | 186.35 (131.43 to 252.77) | -0.1059 (-0.1185 to -0.0933) |
| Andorra | 115 (81 to 156) | 175.71 (124.07 to 237.86) | 197 (141 to 264) | 173.11 (123.47 to 235.2) | -0.0368 (-0.0455 to -0.0282) |
| Angola | 10445 (7212 to 14458) | 138.4 (95.79 to 188.49) | 33231 (23005 to 46610) | 141.15 (99.23 to 193.86) | 0.1319 (0.1034 to 0.1605) |
| Antigua and Barbuda | 85 (59 to 119) | 149.66 (104.26 to 205.61) | 157 (109 to 215) | 149.48 (103.76 to 204.67) | 0.0109 (-0.0013 to 0.023) |
| Argentina | 56543 (39203 to 76069) | 177.48 (123.12 to 238.58) | 87785 (60579 to 118096) | 176.76 (121.75 to 238.35) | -0.007 (-0.0163 to 0.0024) |
| Armenia | 4749 (3374 to 6451) | 140.02 (99.04 to 187.93) | 4936 (3435 to 6644) | 141.98 (98.27 to 193.16) | 0.0795 (0.0679 to 0.0911) |
| Australia | 45472 (33577 to 55294) | 247.44 (182.6 to 301.43) | 71432 (53871 to 86605) | 247.58 (186.2 to 300.2) | 0.0103 (0.0045 to 0.0161) |
| Austria | 15277 (10767 to 20944) | 170.05 (119.82 to 235.04) | 18648 (13241 to 24826) | 169.98 (120.07 to 229.08) | 0.0044 (-0.0034 to 0.0121) |
| Azerbaijan | 9412 (6491 to 13004) | 139.88 (94.17 to 192.47) | 17449 (12196 to 24183) | 142.23 (100.17 to 196.67) | 0.1092 (0.0721 to 0.1463) |
| Bahamas | 360 (248 to 504) | 149.67 (104.52 to 205.66) | 649 (462 to 893) | 147.19 (104.83 to 202.11) | -0.0399 (-0.0477 to -0.0322) |
| Bahrain | 894 (610 to 1242) | 167.98 (117.65 to 231.73) | 3270 (2245 to 4584) | 167.8 (116.55 to 233.31) | 0.0142 (0.0094 to 0.019) |
| Bangladesh | 147376 (102281 to 201490) | 184.91 (129.66 to 248.65) | 303903 (213672 to 419010) | 181.55 (127.73 to 250.72) | -0.0674 (-0.0747 to -0.0601) |
| Barbados | 436 (313 to 580) | 168.63 (120.99 to 222.83) | 562 (390 to 752) | 158.62 (110.54 to 215.68) | -0.0674 (-0.1024 to -0.0324) |
| Belarus | 14746 (10502 to 20103) | 128.9 (91.11 to 177.24) | 14657 (10338 to 19442) | 130.94 (91.44 to 175.78) | 0.0835 (0.064 to 0.1029) |
| Belgium | 19602 (13910 to 26315) | 169.61 (120.45 to 228.95) | 22525 (15260 to 29633) | 167.87 (116.47 to 226.54) | -0.0132 (-0.0253 to -0.0011) |
| Belize | 192 (132 to 267) | 142.54 (99.34 to 198.9) | 600 (418 to 827) | 141.74 (98.97 to 195.99) | -0.0289 (-0.0363 to -0.0215) |
| Benin | 4674 (3357 to 6445) | 144.3 (102.83 to 195.01) | 14463 (10165 to 19926) | 146.24 (101.63 to 200.49) | 0.0432 (0.0341 to 0.0523) |
| Bermuda | 109 (75 to 148) | 156.65 (107.15 to 210.95) | 124 (85 to 167) | 158.38 (111.49 to 215.68) | 0.0535 (0.043 to 0.0639) |
| Bhutan | 888 (612 to 1231) | 187.43 (129.7 to 250.93) | 1556 (1082 to 2126) | 190.33 (134.9 to 257.92) | 0.0391 (0.0331 to 0.0452) |
| Bolivia (Plurinational State of) | 7326 (5093 to 10157) | 147.06 (105.25 to 200.53) | 17515 (12434 to 24116) | 149.05 (104.41 to 204.46) | 0.0528 (0.0424 to 0.0631) |
| Bosnia and Herzegovina | 6659 (4708 to 9056) | 135.94 (96.51 to 184.73) | 5575 (3928 to 7474) | 141.99 (100.21 to 194.76) | 0.1575 (0.1365 to 0.1786) |
| Botswana | 1322 (937 to 1826) | 139.26 (98.52 to 191.06) | 3549 (2439 to 4958) | 142.56 (98.75 to 197.82) | 0.1124 (0.095 to 0.1299) |
| Brazil | 202377 (150092 to 260203) | 152.12 (112.46 to 195.52) | 382072 (278550 to 489511) | 153.38 (112.55 to 197.29) | 0.0523 (0.0373 to 0.0673) |
| Brunei Darussalam | 409 (287 to 572) | 171.17 (122.55 to 236.2) | 905 (636 to 1252) | 167.77 (118.5 to 230.57) | -0.0442 (-0.0532 to -0.0351) |
| Bulgaria | 13567 (9397 to 18362) | 137.93 (96.2 to 189.86) | 11372 (7784 to 15231) | 139.66 (96.59 to 191.71) | 0.061 (0.0489 to 0.0731) |
| Burkina Faso | 8923 (6270 to 12291) | 140.49 (98.09 to 191.81) | 23662 (16618 to 32387) | 143.29 (100.35 to 195.07) | 0.0655 (0.0573 to 0.0736) |
| Burundi | 5322 (3767 to 7438) | 134.81 (94.86 to 185.56) | 13214 (9141 to 18263) | 131.53 (93.2 to 180.67) | -0.067 (-0.0772 to -0.0569) |
| Cabo Verde | 371 (266 to 512) | 153.36 (106.38 to 206.42) | 925 (654 to 1247) | 153.91 (109.85 to 206.31) | 0.0296 (0.0209 to 0.0382) |
| Cambodia | 12558 (8946 to 17351) | 163.61 (116.72 to 221.7) | 29744 (21048 to 40482) | 172.53 (123 to 235.1) | 0.241 (0.2025 to 0.2795) |
| Cameroon | 10678 (7419 to 14781) | 144.18 (101.09 to 198.62) | 35776 (24452 to 49152) | 144.04 (98.78 to 194.38) | 0.0108 (-0.0008 to 0.0225) |
| Canada | 57359 (43499 to 68429) | 185.07 (140.21 to 220.79) | 79715 (60208 to 95298) | 184.22 (139.65 to 220.83) | -0.0102 (-0.0147 to -0.0057) |
| Central African Republic | 2582 (1825 to 3611) | 126.24 (87.41 to 172.63) | 5461 (3751 to 7590) | 124.8 (85.12 to 171.28) | -0.0225 (-0.0336 to -0.0114) |
| Chad | 5737 (4075 to 7958) | 141.09 (99.27 to 191.76) | 16092 (11172 to 22295) | 142.48 (100.96 to 196.95) | 0.0613 (0.0492 to 0.0735) |
| Chile | 22761 (16119 to 31181) | 176.79 (124.81 to 240.53) | 38642 (27416 to 52524) | 178.89 (126.12 to 242.12) | 0.0429 (0.0332 to 0.0526) |
| China | 2329187 (1763156 to 2915190) | 195.67 (147.78 to 244.07) | 3445845 (2572495 to 4306768) | 203.88 (152.53 to 255.67) | 0.0837 (0.0575 to 0.1098) |
| Colombia | 43503 (29326 to 60238) | 153.42 (106.41 to 210.61) | 83986 (59215 to 116485) | 155 (108.87 to 215.38) | 0.0428 (0.0327 to 0.0529) |
| Comoros | 469 (323 to 654) | 144.2 (99.91 to 199.43) | 1018 (710 to 1400) | 143.62 (101.1 to 197.2) | 0.0161 (0.0063 to 0.026) |
| Congo | 2421 (1741 to 3341) | 137.99 (97.23 to 186.12) | 6814 (4758 to 9270) | 138.55 (97.7 to 187.63) | 0.059 (0.0359 to 0.082) |
| Cook Islands | 33 (23 to 46) | 194.67 (136.03 to 268.03) | 37 (27 to 50) | 195.57 (137.22 to 267.66) | 0.0238 (0.0117 to 0.0359) |
| Costa Rica | 4127 (2881 to 5723) | 157.27 (108.29 to 214.37) | 8413 (5862 to 11392) | 157.53 (110.09 to 213.39) | 0.0175 (0.0101 to 0.0248) |
| Croatia | 8029 (5747 to 10825) | 143.1 (101.93 to 195.37) | 7177 (5024 to 9611) | 144.85 (101.51 to 198.84) | 0.0575 (0.0505 to 0.0646) |
| Cuba | 16800 (11747 to 23394) | 148.62 (103.47 to 204.14) | 19797 (14079 to 26787) | 146.75 (104.23 to 199.36) | 0.0044 (-0.0168 to 0.0256) |
| Cyprus | 1395 (978 to 1883) | 170.07 (119.06 to 229.49) | 2896 (2019 to 3873) | 170.57 (119.34 to 230.09) | -0.008 (-0.0137 to -0.0023) |
| Czechia | 16408 (11512 to 22336) | 144.05 (100.66 to 198.42) | 18363 (12832 to 24690) | 146.44 (102.75 to 201.14) | 0.0358 (0.0271 to 0.0445) |
| C么te d'Ivoire | 12428 (8766 to 17589) | 141.4 (99.71 to 195.91) | 32776 (22466 to 45401) | 144.24 (101.47 to 197.91) | 0.0407 (0.0265 to 0.0549) |
| Democratic People's Republic of Korea | 37195 (25685 to 50953) | 178.53 (122.88 to 243.2) | 53903 (38030 to 74512) | 171.76 (121.75 to 239.1) | -0.1535 (-0.1667 to -0.1402) |
| Democratic Republic of the Congo | 36313 (24880 to 50183) | 133.52 (91.82 to 180.53) | 92651 (63824 to 127660) | 131.37 (92.48 to 177.14) | -0.0317 (-0.0695 to 0.006) |
| Denmark | 9470 (6854 to 12495) | 157.37 (114.23 to 207.52) | 15447 (11675 to 18874) | 231.39 (172.97 to 282.75) | 1.772 (1.4487 to 2.0963) |
| Djibouti | 458 (316 to 630) | 144.06 (99.93 to 196.07) | 1785 (1207 to 2460) | 143.34 (97.44 to 196.11) | 0.0043 (-0.0081 to 0.0168) |
| Dominica | 90 (62 to 123) | 144.05 (98.84 to 196.3) | 108 (74 to 145) | 143.9 (98.72 to 195.74) | -0.0006 (-0.0075 to 0.0062) |
| Dominican Republic | 8368 (5857 to 11588) | 142.18 (101.07 to 193.16) | 16573 (11446 to 22729) | 145.54 (100.67 to 198.11) | 0.0916 (0.0845 to 0.0987) |
| Ecuador | 12439 (8648 to 17266) | 152.42 (106.37 to 211.96) | 27766 (19614 to 38231) | 152.34 (107.39 to 208.75) | 0.0267 (0.0145 to 0.039) |
| Egypt | 70779 (49466 to 97895) | 153.97 (107.97 to 209.73) | 153469 (109672 to 211069) | 155.18 (110.19 to 211.67) | 0.0389 (0.0295 to 0.0483) |
| El Salvador | 5947 (4168 to 8231) | 145.03 (101.59 to 198.21) | 9364 (6746 to 12635) | 147.05 (104.52 to 198.07) | 0.0576 (0.0495 to 0.0656) |
| Equatorial Guinea | 401 (287 to 551) | 133.13 (95.15 to 181.14) | 1816 (1269 to 2534) | 147.52 (103.84 to 199.16) | 0.4788 (0.3952 to 0.5624) |
| Eritrea | 3245 (2271 to 4464) | 133.91 (94.03 to 183.73) | 7783 (5271 to 10682) | 136.19 (94.24 to 186.85) | 0.0665 (0.049 to 0.084) |
| Estonia | 2254 (1574 to 3078) | 130.13 (90.36 to 176.62) | 2042 (1410 to 2778) | 134.58 (93.55 to 185.01) | 0.1254 (0.1148 to 0.1359) |
| Eswatini | 757 (530 to 1054) | 139 (96.44 to 190.9) | 1415 (975 to 1942) | 136.74 (95.63 to 186.22) | -0.0399 (-0.0583 to -0.0216) |
| Ethiopia | 48687 (35973 to 63290) | 138.42 (103.23 to 176.34) | 121956 (89922 to 157646) | 141.78 (104.33 to 182.66) | 0.1219 (0.1049 to 0.1389) |
| Fiji | 1227 (860 to 1702) | 182.34 (127.68 to 246.88) | 1729 (1199 to 2371) | 183.94 (127.67 to 251.9) | 0.0479 (0.0378 to 0.058) |
| Finland | 10431 (7582 to 13572) | 176.59 (128.34 to 230.84) | 10936 (7663 to 14617) | 167.62 (115.54 to 226.57) | -0.1731 (-0.2134 to -0.1327) |
| France | 106480 (73897 to 141321) | 165.58 (114.48 to 221.15) | 124342 (86698 to 164639) | 165.09 (115.78 to 224.03) | -0.0169 (-0.0246 to -0.0091) |
| Gabon | 1111 (787 to 1535) | 146.2 (103.6 to 199.48) | 2370 (1644 to 3281) | 145.6 (100.82 to 200.12) | 0.0153 (0.0061 to 0.0245) |
| Gambia | 1003 (700 to 1407) | 144.48 (102.15 to 197.79) | 2666 (1843 to 3677) | 143.05 (99.64 to 196.42) | -0.022 (-0.031 to -0.0131) |
| Georgia | 8257 (5782 to 11404) | 141.01 (98.7 to 194.7) | 5622 (3906 to 7700) | 139.4 (97.95 to 192.95) | 0.0193 (-0.0091 to 0.0477) |
| Germany | 148843 (105716 to 199877) | 154.84 (109.68 to 208.6) | 159031 (113354 to 210122) | 155.11 (111.22 to 207.98) | -0.0161 (-0.034 to 0.0017) |
| Ghana | 16116 (11223 to 22255) | 144.89 (101.97 to 198.53) | 44261 (30266 to 61197) | 148.63 (102.67 to 201.84) | 0.1068 (0.0974 to 0.1162) |
| Greece | 19843 (13868 to 27013) | 168.59 (118.12 to 230.36) | 20599 (14486 to 27144) | 167.2 (117.55 to 227.51) | -0.0198 (-0.0274 to -0.0122) |
| Greenland | 131 (92 to 180) | 210.46 (148.77 to 284.5) | 136 (97 to 182) | 209.62 (149.26 to 281.77) | 0.0873 (0.0445 to 0.1302) |
| Grenada | 99 (69 to 135) | 142.59 (99.81 to 194.06) | 167 (118 to 224) | 145.34 (101.77 to 196.04) | 0.0689 (0.0617 to 0.076) |
| Guam | 280 (198 to 392) | 205.89 (145.55 to 283.58) | 343 (249 to 465) | 201.85 (146.46 to 273.9) | -0.0392 (-0.0501 to -0.0282) |
| Guatemala | 8205 (5821 to 11258) | 143.2 (101.84 to 194.29) | 21218 (15128 to 29097) | 144.41 (101.77 to 198) | 0.0273 (0.0179 to 0.0366) |
| Guinea | 6373 (4459 to 8770) | 144.49 (100.02 to 196.64) | 14182 (9952 to 19579) | 143.81 (101.76 to 196.72) | -0.0062 (-0.0202 to 0.0078) |
| Guinea-Bissau | 969 (673 to 1339) | 139.57 (97.14 to 188.05) | 2228 (1562 to 3076) | 140.13 (98.65 to 189.85) | -0.004 (-0.013 to 0.005) |
| Guyana | 868 (607 to 1224) | 133.69 (94.05 to 182.85) | 1054 (734 to 1452) | 136.12 (94.43 to 186.58) | 0.0509 (0.0447 to 0.0571) |
| Haiti | 6458 (4383 to 8850) | 129.53 (88.59 to 177.47) | 15357 (11021 to 21144) | 127.21 (90.21 to 173.49) | -0.0429 (-0.0497 to -0.0362) |
| Honduras | 4815 (3393 to 6657) | 146.69 (103.49 to 199.61) | 13542 (9444 to 18567) | 144.79 (101.35 to 195.13) | -0.0323 (-0.0437 to -0.021) |
| Hungary | 16288 (11206 to 22161) | 139.44 (95.82 to 190.79) | 16400 (11657 to 21947) | 143.28 (101.85 to 193.81) | 0.0889 (0.0781 to 0.0996) |
| Iceland | 453 (316 to 621) | 172.19 (120.04 to 235.92) | 692 (473 to 937) | 172.59 (118.3 to 236.8) | 0.0008 (-0.0057 to 0.0073) |
| India | 1325494 (982326 to 1705183) | 180.74 (133.57 to 231.61) | 2781659 (2073729 to 3593445) | 187.66 (140.05 to 241.67) | 0.1568 (0.1235 to 0.1901) |
| Indonesia | 294863 (217975 to 382498) | 180.41 (133.44 to 230.69) | 578068 (429388 to 731017) | 186.05 (138.8 to 235.11) | 0.1567 (0.1236 to 0.1898) |
| Iran (Islamic Republic of) | 66785 (49388 to 86252) | 157.9 (116.85 to 202.2) | 158909 (117439 to 203707) | 158.93 (118.37 to 202.63) | 0.0277 (0.0205 to 0.035) |
| Iraq | 20608 (14396 to 28125) | 153.76 (109.08 to 207.04) | 60599 (42306 to 82918) | 153.35 (108.1 to 209.01) | 0.0437 (0.0262 to 0.0612) |
| Ireland | 7337 (5308 to 9512) | 206.19 (149.22 to 267.34) | 11545 (8102 to 15454) | 205.04 (144.29 to 276.5) | 0.1232 (0.0386 to 0.2079) |
| Israel | 8129 (5785 to 10849) | 174.98 (124.3 to 233.37) | 16484 (11519 to 22133) | 170.95 (119.31 to 230.23) | -0.0422 (-0.0608 to -0.0235) |
| Italy | 106325 (78674 to 136009) | 160.31 (117.86 to 205.62) | 117386 (86079 to 149280) | 161.14 (119.53 to 206.72) | 0.0223 (0.0178 to 0.0268) |
| Jamaica | 2901 (2005 to 4023) | 146.12 (100.49 to 197.97) | 4524 (3141 to 6107) | 145.29 (100.14 to 196.15) | -0.0184 (-0.0235 to -0.0133) |
| Japan | 268804 (198622 to 344319) | 183.66 (136.65 to 236.57) | 261724 (192639 to 329975) | 181.14 (134.29 to 232.07) | 0.112 (0.0164 to 0.2077) |
| Jordan | 4338 (2936 to 6060) | 159.86 (113.19 to 216.04) | 19715 (13930 to 27659) | 158.8 (114.16 to 221.64) | -0.02 (-0.0305 to -0.0095) |
| Kazakhstan | 21874 (15359 to 30242) | 137.88 (96.31 to 188.81) | 28335 (19886 to 38932) | 141.15 (100.02 to 193.66) | 0.1188 (0.0894 to 0.1481) |
| Kenya | 21808 (15966 to 28289) | 141.55 (104.25 to 183.22) | 60604 (44504 to 77974) | 142.16 (104.68 to 182.99) | 0.0478 (0.0301 to 0.0656) |
| Kiribati | 104 (73 to 145) | 165.83 (117.16 to 226.18) | 186 (129 to 251) | 164.06 (113.87 to 219.56) | -0.0231 (-0.0321 to -0.014) |
| Kuwait | 3087 (2094 to 4353) | 172.81 (121.11 to 237.17) | 10616 (7273 to 14624) | 169.24 (116.74 to 231.15) | -0.0774 (-0.095 to -0.0598) |
| Kyrgyzstan | 5115 (3600 to 7115) | 135.35 (95.69 to 186.67) | 8957 (6236 to 12139) | 134.37 (94.22 to 184.15) | -0.0021 (-0.024 to 0.0197) |
| Lao People's Democratic Republic | 5322 (3829 to 7564) | 167.46 (117.89 to 234.47) | 12964 (9128 to 17745) | 177.15 (127.01 to 239.46) | 0.2576 (0.2239 to 0.2914) |
| Latvia | 3825 (2665 to 5187) | 129.49 (90.31 to 176.12) | 2873 (2002 to 3933) | 132.61 (92.86 to 182.85) | 0.1273 (0.113 to 0.1416) |
| Lebanon | 4242 (3011 to 5902) | 158.78 (112.28 to 218.31) | 9898 (6807 to 13572) | 158.25 (110.24 to 216.68) | 0.0075 (0.0001 to 0.0148) |
| Lesotho | 1480 (1047 to 2016) | 129.7 (91.22 to 175.08) | 2196 (1549 to 3047) | 129.04 (90.79 to 175.43) | 0.0003 (-0.01 to 0.0107) |
| Liberia | 2485 (1779 to 3410) | 138.94 (99.35 to 186.43) | 6241 (4312 to 8626) | 137.28 (95.08 to 185.85) | 0.0091 (-0.0118 to 0.0299) |
| Libya | 5175 (3567 to 7286) | 164.76 (114.43 to 225.47) | 12645 (8762 to 17305) | 156.09 (108.45 to 212.39) | -0.1771 (-0.1999 to -0.1543) |
| Lithuania | 5185 (3680 to 7107) | 129.62 (91.55 to 177) | 4224 (2994 to 5772) | 133.11 (93.28 to 183.8) | 0.1141 (0.1016 to 0.1266) |
| Luxembourg | 785 (543 to 1053) | 171.59 (119.73 to 231.38) | 1393 (978 to 1877) | 173.53 (122.91 to 234.53) | 0.0441 (0.0365 to 0.0516) |
| Madagascar | 11815 (8075 to 16443) | 138.89 (95.83 to 192) | 31652 (22208 to 44354) | 138.79 (97.28 to 189.41) | 0.033 (0.0222 to 0.0439) |
| Malawi | 9078 (6235 to 12413) | 130.9 (91.33 to 179.25) | 19441 (13523 to 26614) | 131.54 (93.41 to 182.2) | 0.0664 (0.0486 to 0.0842) |
| Malaysia | 29498 (20790 to 42196) | 189.04 (133.04 to 266.36) | 68763 (49315 to 96459) | 196.25 (141.23 to 272.62) | 0.1702 (0.1426 to 0.1978) |
| Maldives | 293 (206 to 411) | 188.52 (134.05 to 258.58) | 1352 (947 to 1900) | 200.74 (143.69 to 277.15) | 0.2667 (0.226 to 0.3074) |
| Mali | 8678 (6083 to 11871) | 141.49 (98.35 to 192.43) | 23382 (15972 to 32469) | 143.82 (100.29 to 196.6) | 0.07 (0.0628 to 0.0772) |
| Malta | 699 (480 to 942) | 169.72 (118.2 to 229.1) | 913 (645 to 1227) | 171.16 (121.21 to 229.76) | 0.0186 (0.0093 to 0.0279) |
| Marshall Islands | 54 (37 to 75) | 171.93 (122.63 to 232.91) | 95 (67 to 132) | 170.25 (120.1 to 233.71) | -0.0341 (-0.047 to -0.0211) |
| Mauritania | 2236 (1557 to 3104) | 149.14 (103.9 to 202.78) | 5109 (3624 to 7028) | 151.1 (106.24 to 203.74) | 0.0477 (0.0408 to 0.0547) |
| Mauritius | 2051 (1396 to 2859) | 186.19 (128.45 to 256.05) | 2856 (2058 to 3884) | 190.69 (137.12 to 262.44) | 0.1254 (0.0999 to 0.1509) |
| Mexico | 107876 (80611 to 139851) | 157.22 (116.59 to 202.94) | 218631 (162671 to 281593) | 157.99 (117.62 to 203.56) | 0.007 (-0.0014 to 0.0155) |
| Micronesia (Federated States of) | 132 (93 to 185) | 171.17 (120.04 to 234.7) | 172 (121 to 237) | 170.52 (119.84 to 233.15) | -0.0071 (-0.0163 to 0.0021) |
| Monaco | 70 (48 to 94) | 181.96 (123.68 to 247.84) | 84 (59 to 110) | 181.74 (127.11 to 243.38) | -0.0176 (-0.0311 to -0.0041) |
| Mongolia | 2190 (1509 to 3047) | 132.31 (91.58 to 179.36) | 4667 (3311 to 6385) | 136.66 (97.88 to 187.25) | 0.1286 (0.1125 to 0.1448) |
| Montenegro | 931 (640 to 1276) | 141.67 (97.18 to 193.7) | 1014 (712 to 1361) | 141.57 (100.23 to 193.51) | 0.0373 (0.0276 to 0.047) |
| Morocco | 32164 (22450 to 44480) | 152.31 (108.19 to 207.92) | 59745 (41789 to 82109) | 151.73 (106.42 to 208.61) | -0.0133 (-0.025 to -0.0016) |
| Mozambique | 12250 (8558 to 16618) | 126.32 (87.64 to 171.47) | 28639 (20179 to 39795) | 129.44 (90.19 to 175.42) | 0.1429 (0.1216 to 0.1643) |
| Myanmar | 57713 (39904 to 79913) | 163.24 (113.76 to 222.31) | 100678 (70931 to 138999) | 173.89 (122.71 to 239.61) | 0.3119 (0.2684 to 0.3555) |
| Namibia | 1475 (1019 to 2046) | 141.24 (97.49 to 191.07) | 3199 (2243 to 4400) | 142.34 (100.73 to 194.21) | 0.0705 (0.0535 to 0.0875) |
| Nauru | 16 (11 to 22) | 190.51 (133.85 to 260.17) | 18 (13 to 25) | 179.92 (127.86 to 245.42) | -0.1626 (-0.2505 to -0.0746) |
| Nepal | 26930 (18609 to 36641) | 178.8 (125.22 to 240.43) | 52664 (37321 to 72239) | 175.31 (123.7 to 240.1) | -0.0807 (-0.0957 to -0.0656) |
| Netherlands | 42175 (30859 to 53489) | 244.44 (179.12 to 309.94) | 47329 (34644 to 61356) | 233.55 (169.99 to 304.99) | -0.1698 (-0.2157 to -0.1238) |
| New Zealand | 8713 (6505 to 11203) | 239.65 (178.85 to 307.93) | 13988 (10516 to 17838) | 243.41 (181.92 to 311.89) | 0.043 (0.0336 to 0.0524) |
| Nicaragua | 4019 (2840 to 5565) | 150.35 (106.64 to 205.33) | 9941 (7065 to 13710) | 149.95 (107.5 to 204.34) | 0.0129 (-0.0032 to 0.029) |
| Niger | 7535 (5227 to 10401) | 141.45 (98.32 to 191.65) | 22112 (15588 to 30498) | 142 (101.51 to 192.7) | 0.0208 (0.0109 to 0.0307) |
| Nigeria | 103176 (75311 to 133026) | 151.94 (111.79 to 196.24) | 270855 (200286 to 347619) | 158.09 (117.46 to 203.47) | 0.1851 (0.1634 to 0.2069) |
| Niue | 4 (3 to 5) | 184.68 (130.7 to 255.17) | 3 (2 to 5) | 185.95 (131.65 to 256.02) | 0.0592 (0.0481 to 0.0704) |
| North Macedonia | 2950 (2026 to 4086) | 141.08 (97.08 to 194.9) | 3831 (2661 to 5158) | 141.42 (97.64 to 191.21) | 0.0318 (0.023 to 0.0406) |
| Northern Mariana Islands | 103 (72 to 146) | 208.91 (146.71 to 289.65) | 108 (77 to 147) | 199.2 (140.5 to 274.5) | -0.158 (-0.1814 to -0.1346) |
| Norway | 8269 (6134 to 10468) | 174.41 (129.23 to 222.02) | 11128 (8267 to 14059) | 175.28 (130.43 to 223.41) | 0.0397 (0.0312 to 0.0482) |
| Oman | 2810 (1937 to 3896) | 164.55 (115.57 to 225.43) | 9503 (6572 to 12962) | 165.36 (115.57 to 223) | 0.0201 (0.0121 to 0.0281) |
| Pakistan | 153536 (114269 to 198832) | 188.12 (139.32 to 242.62) | 385932 (287150 to 501493) | 184.26 (137.44 to 238.69) | -0.0827 (-0.0902 to -0.0752) |
| Palau | 28 (20 to 39) | 186.33 (131.77 to 256.52) | 41 (29 to 56) | 188.14 (132.81 to 257.17) | 0.0226 (0.0013 to 0.0439) |
| Palestine | 2155 (1516 to 2984) | 154.95 (109.03 to 210.02) | 6999 (4973 to 9734) | 153.58 (109.15 to 210.65) | -0.0518 (-0.0685 to -0.0351) |
| Panama | 3278 (2288 to 4510) | 156.37 (109.38 to 211.38) | 6998 (4949 to 9574) | 159.41 (112.62 to 218.07) | 0.0651 (0.0543 to 0.0759) |
| Papua New Guinea | 5717 (3971 to 7961) | 173.27 (121.27 to 237.99) | 16420 (11616 to 22717) | 175.07 (123.35 to 240.82) | 0.0219 (0.0046 to 0.0392) |
| Paraguay | 4920 (3414 to 6783) | 153.51 (107.66 to 208.75) | 11185 (7805 to 15416) | 153.93 (108.31 to 209.84) | 0.0052 (-0.004 to 0.0143) |
| Peru | 27284 (18940 to 37717) | 153.57 (106.24 to 207.56) | 59290 (41804 to 81120) | 155.92 (109.18 to 213.29) | 0.0793 (0.065 to 0.0937) |
| Philippines | 93782 (69446 to 121585) | 180.41 (134.03 to 230.88) | 207177 (153152 to 265765) | 183.73 (136.85 to 234.82) | 0.1103 (0.0833 to 0.1373) |
| Poland | 57765 (42777 to 74186) | 140.17 (103.78 to 179.92) | 67086 (49305 to 85750) | 145.23 (108.24 to 187.34) | 0.124 (0.1155 to 0.1325) |
| Portugal | 18217 (12919 to 24760) | 163.67 (115.93 to 224.63) | 21445 (15080 to 28000) | 165.37 (116.55 to 220.81) | 0.0151 (0.0053 to 0.0249) |
| Puerto Rico | 5446 (3855 to 7449) | 150.68 (106.4 to 206.59) | 5796 (4009 to 7718) | 151.38 (105.49 to 205.06) | 0.0355 (0.025 to 0.046) |
| Qatar | 922 (623 to 1303) | 175.51 (123.17 to 244.39) | 7665 (5100 to 10897) | 176.52 (119.92 to 247.32) | 0.0854 (0.0656 to 0.1052) |
| Republic of Korea | 76586 (54116 to 105995) | 165.19 (117.01 to 227.06) | 111195 (77617 to 151722) | 167.37 (114.46 to 230.94) | 0.0307 (0.0228 to 0.0386) |
| Republic of Moldova | 5857 (4154 to 8064) | 126.43 (89.69 to 173.83) | 5718 (3997 to 7693) | 127.35 (88.41 to 173.14) | 0.0257 (0.0082 to 0.0432) |
| Romania | 34475 (23793 to 46655) | 138.48 (94.84 to 189.41) | 31579 (22152 to 42709) | 142.2 (99.78 to 195.6) | 0.1239 (0.1119 to 0.1358) |
| Russian Federation | 203951 (150343 to 263148) | 121.27 (89.5 to 156.28) | 225240 (165472 to 287814) | 131.29 (97.23 to 168.51) | 0.4034 (0.3205 to 0.4864) |
| Rwanda | 6905 (4776 to 9616) | 136.44 (96.89 to 188.24) | 15726 (10948 to 21707) | 137.9 (95.05 to 185.58) | 0.104 (0.0692 to 0.1388) |
| Saint Kitts and Nevis | 51 (36 to 71) | 144.7 (99.39 to 198.24) | 107 (74 to 146) | 148.56 (104.82 to 203.5) | 0.0838 (0.0747 to 0.0928) |
| Saint Lucia | 158 (109 to 220) | 143.22 (99.79 to 194.52) | 310 (217 to 427) | 144.95 (102.82 to 201.03) | 0.0262 (0.0164 to 0.0359) |
| Saint Vincent and the Grenadines | 124 (84 to 173) | 141.39 (98.14 to 191.78) | 180 (124 to 241) | 142.69 (98.17 to 193.02) | 0.036 (0.0271 to 0.0449) |
| Samoa | 228 (161 to 323) | 179.85 (129.41 to 248.83) | 332 (236 to 463) | 180.43 (126.93 to 248.4) | 0.0204 (0.0142 to 0.0266) |
| San Marino | 48 (33 to 64) | 174.98 (119.08 to 234.76) | 68 (48 to 92) | 172.11 (118.32 to 235.76) | -0.0507 (-0.0603 to -0.0411) |
| Sao Tome and Principe | 123 (86 to 170) | 151.23 (102.79 to 205.61) | 292 (206 to 399) | 151.84 (107.65 to 207.02) | 0.0216 (0.0142 to 0.0291) |
| Saudi Arabia | 21257 (14541 to 29473) | 164.03 (114.77 to 224.6) | 79283 (54248 to 110828) | 163.17 (114.14 to 224.14) | -0.0115 (-0.0257 to 0.0027) |
| Senegal | 7670 (5465 to 10712) | 147.28 (104.04 to 201.63) | 18772 (13318 to 25894) | 147.89 (105.59 to 202.92) | 0.032 (0.0243 to 0.0398) |
| Serbia | 15139 (10478 to 20670) | 141.33 (97.62 to 194.36) | 14945 (10537 to 20198) | 141.66 (99.51 to 193.55) | 0.0417 (0.0245 to 0.0589) |
| Seychelles | 127 (90 to 177) | 191 (135.87 to 263.98) | 235 (168 to 325) | 195.2 (140.99 to 271.3) | 0.1092 (0.0827 to 0.1357) |
| Sierra Leone | 4506 (3123 to 6191) | 142.05 (99.99 to 193.69) | 10080 (6965 to 14073) | 141.44 (97.29 to 191.95) | -0.0153 (-0.0332 to 0.0026) |
| Singapore | 5704 (3975 to 7836) | 163.8 (113.84 to 223.34) | 12604 (8752 to 17009) | 165.87 (116.34 to 224.03) | 0.0323 (0.0229 to 0.0417) |
| Slovakia | 7929 (5603 to 10927) | 141.36 (99.89 to 195.87) | 9563 (6729 to 13022) | 144.59 (100.24 to 197.45) | 0.0968 (0.0888 to 0.1048) |
| Slovenia | 3192 (2241 to 4390) | 143.05 (99.8 to 197.91) | 3595 (2482 to 4902) | 146.68 (102.25 to 200.95) | 0.0653 (0.0564 to 0.0743) |
| Solomon Islands | 406 (289 to 570) | 168.54 (117.81 to 232.01) | 995 (686 to 1386) | 167.08 (116.62 to 230.51) | -0.0382 (-0.058 to -0.0183) |
| Somalia | 7094 (4986 to 9930) | 127.27 (88.82 to 173.65) | 18720 (13179 to 25883) | 124.77 (88.99 to 168.39) | -0.0532 (-0.0644 to -0.0419) |
| South Africa | 45409 (33237 to 58393) | 143.6 (106.85 to 184.97) | 86535 (64068 to 111295) | 141.77 (105.17 to 181.78) | -0.0125 (-0.0251 to 0.0002) |
| South Sudan | 5957 (4173 to 8425) | 141.61 (101.14 to 192.86) | 9742 (6825 to 13201) | 137.38 (97.42 to 183.3) | -0.0518 (-0.0822 to -0.0215) |
| Spain | 71804 (54028 to 86113) | 169.05 (127.53 to 203.1) | 95958 (72852 to 116033) | 170.29 (130.39 to 207.74) | 0.0225 (0.0144 to 0.0307) |
| Sri Lanka | 29802 (21030 to 41748) | 181.14 (128.2 to 250.82) | 44841 (31442 to 61027) | 186.59 (131.49 to 254.84) | 0.1665 (0.1325 to 0.2006) |
| Sudan | 21714 (15066 to 30175) | 146.02 (102.37 to 197.09) | 54668 (38507 to 75577) | 147.4 (105.3 to 200.56) | 0.0294 (0.0195 to 0.0392) |
| Suriname | 435 (315 to 565) | 126.49 (91.16 to 164.02) | 780 (572 to 989) | 125.54 (92.05 to 159.5) | 0.0047 (-0.0071 to 0.0166) |
| Sweden | 17827 (13011 to 22461) | 177.69 (129.13 to 223.98) | 21413 (15446 to 27384) | 174.7 (129.3 to 225.04) | -0.0607 (-0.0733 to -0.0481) |
| Switzerland | 14142 (10093 to 19019) | 172.93 (123.77 to 232.72) | 18794 (12892 to 24928) | 172.47 (118.52 to 232.61) | 0.0104 (-0.0026 to 0.0234) |
| Syrian Arab Republic | 13712 (9374 to 19048) | 154.7 (108.61 to 208.11) | 20955 (14766 to 28670) | 150.63 (106.88 to 204.91) | -0.0774 (-0.0991 to -0.0557) |
| Taiwan (Province of China) | 40369 (28432 to 55366) | 190.27 (135.34 to 257.68) | 60048 (42675 to 80386) | 202.09 (143.27 to 277.9) | 0.1335 (0.1059 to 0.1611) |
| Tajikistan | 5478 (3828 to 7566) | 136.75 (96.57 to 187.96) | 12751 (8885 to 17687) | 134.25 (94.11 to 184.46) | -0.0269 (-0.055 to 0.0012) |
| Thailand | 101595 (70169 to 141257) | 180.28 (125.62 to 245.94) | 150733 (106024 to 206305) | 187.09 (131.99 to 256.12) | 0.1715 (0.1471 to 0.1959) |
| Timor-Leste | 1009 (705 to 1419) | 158.8 (112.08 to 219.27) | 1878 (1326 to 2625) | 162.49 (114 to 222.89) | 0.2006 (0.1505 to 0.2506) |
| Togo | 3537 (2475 to 4933) | 142.72 (101.17 to 197.04) | 10066 (7056 to 13802) | 142.95 (99.97 to 193.28) | 0.0074 (-0.0017 to 0.0165) |
| Tokelau | 2 (2 to 3) | 177.81 (127.59 to 241.92) | 2 (2 to 3) | 181.07 (129.8 to 246.07) | 0.0856 (0.0724 to 0.0988) |
| Tonga | 135 (95 to 188) | 182.81 (127.58 to 250.14) | 166 (121 to 225) | 182.66 (131.96 to 245.66) | 0.0034 (-0.0067 to 0.0134) |
| Trinidad and Tobago | 1281 (950 to 1642) | 116.72 (85.06 to 149.61) | 2407 (1676 to 3219) | 148.18 (104.07 to 200.51) | 0.5881 (0.3741 to 0.8026) |
| Tunisia | 11217 (7823 to 15479) | 158.17 (109.82 to 215.5) | 20791 (14619 to 28467) | 156.68 (111.97 to 216.91) | -0.0226 (-0.0326 to -0.0126) |
| Turkey | 79588 (58712 to 103792) | 156.82 (115.86 to 203.59) | 147441 (109038 to 191910) | 157.39 (116.54 to 204.67) | 0.0116 (0.0054 to 0.0179) |
| Turkmenistan | 4033 (2807 to 5582) | 137.04 (95.88 to 185.14) | 7397 (5235 to 9993) | 141.23 (100.26 to 190.5) | 0.1265 (0.0972 to 0.1557) |
| Tuvalu | 15 (10 to 20) | 169.63 (120.27 to 233.1) | 21 (15 to 28) | 174.61 (121.92 to 236.23) | 0.1067 (0.0994 to 0.1139) |
| Uganda | 14829 (10448 to 20470) | 130.03 (90.01 to 177.79) | 41092 (28550 to 57473) | 134.98 (95.67 to 185.7) | 0.158 (0.1444 to 0.1717) |
| Ukraine | 75860 (55058 to 99109) | 129.77 (94.14 to 170.6) | 67672 (49055 to 86448) | 128.25 (93.89 to 166.01) | 0.0059 (-0.0146 to 0.0264) |
| United Arab Emirates | 3690 (2523 to 5206) | 177.21 (125.73 to 242.07) | 25401 (17134 to 34835) | 170.29 (118.91 to 235.62) | -0.1178 (-0.1288 to -0.1068) |
| United Kingdom | 100550 (74668 to 126989) | 155.91 (115.27 to 198.07) | 107987 (79459 to 136407) | 135.94 (100.05 to 173.08) | -0.3352 (-0.4728 to -0.1974) |
| United Republic of Tanzania | 24016 (16682 to 33556) | 134.65 (95.21 to 183.8) | 63340 (44723 to 87449) | 138.53 (97.36 to 188.59) | 0.1487 (0.1344 to 0.1631) |
| United States of America | 160 (114 to 220) | 149.88 (107.17 to 206.45) | 152 (108 to 205) | 152.23 (107.28 to 210.14) | 0.0437 (0.0229 to 0.0646) |
| United States Virgin Islands | 660739 (489984 to 841532) | 235.03 (173.66 to 299.91) | 824552 (617697 to 1040824) | 221.4 (164.64 to 281.52) | -0.133 (-0.1912 to -0.0748) |
| Uruguay | 5660 (3877 to 7666) | 178.12 (122.19 to 242.43) | 6676 (4661 to 8966) | 177.53 (124.9 to 240.31) | -0.0132 (-0.0285 to 0.002) |
| Uzbekistan | 22287 (15565 to 30783) | 134.61 (94.41 to 183.29) | 49045 (34531 to 67184) | 136.71 (96.23 to 185.63) | 0.0702 (0.0521 to 0.0883) |
| Vanuatu | 198 (137 to 273) | 171.86 (120.89 to 234.53) | 471 (331 to 656) | 170.61 (119.51 to 235.26) | -0.0107 (-0.0208 to -0.0005) |
| Venezuela (Bolivarian Republic of) | 24753 (17142 to 33975) | 155.65 (107.89 to 211.37) | 44276 (30411 to 59980) | 152.92 (106.22 to 207.16) | -0.0162 (-0.0354 to 0.003) |
| Viet Nam | 113960 (78978 to 155540) | 199.16 (138.3 to 270.05) | 233506 (163605 to 318209) | 209 (148.09 to 283.33) | 0.2079 (0.188 to 0.2278) |
| Yemen | 12852 (8833 to 17802) | 145.22 (103.15 to 197.6) | 39429 (28252 to 54373) | 142.58 (102.34 to 195.51) | -0.0414 (-0.0608 to -0.0221) |
| Zambia | 7161 (5012 to 9954) | 134.99 (94.74 to 183.21) | 20850 (14810 to 28987) | 137.25 (97.67 to 187.61) | 0.1216 (0.0935 to 0.1496) |
| Zimbabwe | 9582 (6620 to 13328) | 135.36 (92.41 to 184.48) | 16711 (11664 to 23310) | 131.21 (91.44 to 183.22) | -0.1152 (-0.1417 to -0.0887) |

**Supplementary Table S5** Joinpoint regression analysis: trends in age-standardized incidence, prevalence, DALYs (per 100,000 persons) among both sexes, males, and females, 1990–2021

| Gender | ASIR | | | ASPR | | | ASDR | | |
| --- | --- | --- | --- | --- | --- | --- | --- | --- | --- |
|  | Period | APC (95%CI) | AAPC (95%CI) | Period | APC (95%CI) | AAPC (95%CI) | Period | APC (95%CI) | AAPC (95%CI) |
| Both | 1990-2000 | -0.09 (-0.09 - -0.08) | -0.04 (-0.05 - -0.04) | 1990-1993 | -0.00 (-0.06 - 0.05) | 0.02 (0.01 - 0.03) | 1990-1994 | 0.02 (-0.00 - 0.04) | 0.02 (0.01 - 0.03) |
|  | 2000-2005 | -0.01 (-0.03 - 0.00) |  | 1993-2005 | -0.04 (-0.05 - -0.03) |  | 1994-2002 | -0.05 (-0.06 - -0.04) |  |
|  | 2005-2009 | 0.05 (0.02 - 0.08) |  | 2005-2009 | 0.17 (0.12 - 0.23) |  | 2002-2006 | 0.05 (0.01 - 0.08) |  |
|  | 2009-2015 | -0.06 (-0.07 - -0.04) |  | 2009-2014 | 0.08 (0.05 - 0.11) |  | 2006-2009 | 0.22 (0.16 - 0.29) |  |
|  | 2015-2018 | -0.10 (-0.16 - -0.04) |  | 2014-2018 | -0.03 (-0.09 - 0.02) |  | 2009-2014 | 0.07 (0.05 - 0.09) |  |
|  | 2018-2021 | 0.02 (-0.01 - 0.05) |  | 2018-2021 | 0.08 (0.03 - 0.13) |  | 2014-2021 | -0.03 (-0.04 - -0.02) |  |
| Female | 1990-1997 | -0.11 (-0.12 - -0.11) | -0.04 (-0.04 - -0.03) | 1990-2005 | -0.04 (-0.04 - -0.04) | 0.02 (0.01 - 0.02) | 1990-1992 | 0.02 (-0.05 - 0.09) | 0.01 (0.00 - 0.02) |
|  | 1997-2000 | -0.06 (-0.11 - -0.01) |  | 2005-2008 | 0.11 (0.08 - 0.14) |  | 1992-2005 | -0.03 (-0.04 - -0.03) |  |
|  | 2000-2007 | 0.03 (0.02 - 0.04) |  | 2008-2011 | 0.07 (0.04 - 0.10) |  | 2005-2009 | 0.13 (0.10 - 0.17) |  |
|  | 2007-2014 | -0.03 (-0.04 - -0.03) |  | 2011-2014 | 0.14 (0.11 - 0.18) |  | 2009-2015 | 0.09 (0.07 - 0.11) |  |
|  | 2014-2019 | -0.04 (-0.06 - -0.03) |  | 2014-2019 | 0.00 (-0.01 - 0.02) |  | 2015-2019 | -0.02 (-0.06 - 0.01) |  |
|  | 2019-2021 | 0.06 (0.01 - 0.11) |  | 2019-2021 | 0.11 (0.08 - 0.14) |  | 2019-2021 | -0.10 (-0.17 - -0.03) |  |
| Male | 1990-1995 | -0.04 (-0.06 - -0.03) | -0.05 (-0.06 - -0.04) | 1990-1995 | 0.02 (-0.02 - 0.06) | 0.03 (0.01 - 0.04) | 1990-1995 | 0.03 (-0.00 - 0.06) | 0.03 (0.01 - 0.05) |
|  | 1995-1999 | -0.11 (-0.15 - -0.07) |  | 1995-2000 | -0.08 (-0.13 - -0.03) |  | 1995-2000 | -0.07 (-0.11 - -0.02) |  |
|  | 1999-2005 | -0.05 (-0.07 - -0.04) |  | 2000-2005 | 0.02 (-0.04 - 0.07) |  | 2000-2005 | 0.03 (-0.01 - 0.08) |  |
|  | 2005-2010 | 0.07 (0.04 - 0.09) |  | 2005-2011 | 0.19 (0.15 - 0.23) |  | 2005-2010 | 0.23 (0.19 - 0.28) |  |
|  | 2010-2019 | -0.10 (-0.11 - -0.09) |  | 2011-2018 | -0.03 (-0.06 - -0.00) |  | 2010-2013 | 0.03 (-0.11 - 0.17) |  |
|  | 2019-2021 | 0.02 (-0.06 - 0.10) |  | 2018-2021 | 0.06 (-0.02 - 0.14) |  | 2013-2021 | -0.04 (-0.05 - -0.02) |  |
